# Gene Expression Profiles Associated with Pediatric Relapsed AML

**DOI:** 10.1371/journal.pone.0121730

**Published:** 2015-04-07

**Authors:** Costa Bachas, Gerrit Jan Schuurhuis, C. Michel Zwaan, Marry M. van den Heuvel-Eibrink, Monique L. den Boer, Eveline S. J. M. de Bont, Zinia J. Kwidama, Dirk Reinhardt, Ursula Creutzig, Valérie de Haas, Gertjan J. L. Kaspers, Jacqueline Cloos

**Affiliations:** 1 Department of Pediatric Oncology/Hematology, VU University Medical Center, Amsterdam, The Netherlands; 2 Department of Hematology, VU University Medical Center, Amsterdam, The Netherlands; 3 Department of Pediatric Oncology/Hematology, Erasmus MC/Sophia Children’s Hospital, Rotterdam, The Netherlands; 4 Division of Pediatric Oncology/Hematology, Department of Pediatrics, University of Groningen, University Medical Center Groningen, Groningen, The Netherlands; 5 AML-BFM Study Group, Department of Pediatric Hematology/ Oncology, Medical School Hannover, Hannover, Germany; 6 Dutch Childhood Oncology Group (DCOG), The Hague, The Netherlands; Emory University, UNITED STATES

## Abstract

Development of relapse remains a problem for further improvements in the survival of pediatric AML patients. While virtually all patients show a good response to initial treatment, more patients respond poorly when treated at relapse. The cellular characteristics of leukemic blast cells that allow survival of initial treatment, relapse development and subsequent resistance to salvage treatment remain largely elusive. Therefore, we studied if leukemic blasts at relapse biologically resemble their initial diagnosis counterparts. We performed microarray gene expression profiling on paired initial and relapse samples of 23 pediatric AML patients. In 11 out of 23 patients, gene expression profiles of initial and corresponding relapse samples end up in different clusters in unsupervised analysis, indicating altered gene expression profiles. In addition, shifts in type I/II mutational status were found in 5 of these 11 patients, while shifts were found in 3 of the remaining 12 patients. Although differentially expressed genes varied between patients, they were commonly related to hematopoietic differentiation, encompassed genes involved in chromatin remodeling and showed associations with similar transcription factors. The top five were *CEBPA*, *GFI1*, *SATB1*, *KLF2* and *TBP*. In conclusion, the leukemic blasts at relapse are biologically different from their diagnosis counterparts. These differences may be exploited for further development of novel treatment strategies.

## Introduction

In high income countries, the vast majority (over 90%) of pediatric acute myeloid leukemia (AML) patients achieve complete remission (CR) with current intensive chemotherapy protocols [1]. However, even with optimal therapies, 30–40% of patients relapse and face a dismal prognosis [2–4]. Long term survival rates have only marginally increased over recent decades and stabilized at approximately 65% [5].

Approximately 85% of the patients show a good early response to the initial induction treatment in terms of blast reduction [6,7]. However, a poor response at initial diagnosis [6,7] and/or later at relapse [4] is among the strongest adverse prognostic factors for outcome in pediatric AML [8]. Apparently, therapy is insufficient to relieve over a quarter of the patients from a burden of persistent leukemia that causes relapses and fatal outcome.

A number of cell-biological factors determined at initial diagnosis are known to be associated with an increased risk of relapse, including genetic characteristics such as abnormal karyotype (e.g. certain 11q23 translocations or monosomy 7 [9,10]) or gene mutations such *FLT3/ITD* and *WT1* [11]. Moreover, aberrant gene expression (e.g. *EVI-1*, *BAALC*, *WT1)* has also been reported to be associated with the risk of relapse [12,13]. The mechanisms by which these factors act in relapse development are largely unclear.

The mainstay of salvage regimens in relapsed AML is, as in initial treatment, cytarabine and anthracycline based [2,14], hence drug resistance is likely to play a role in relapse development. Although pharmacological factors may be involved in resistance to therapy [15], cellular drug resistance is thought to contribute to poor response to therapy as well [16,17].

Previous studies with paired initial and relapsed AML samples showed that the mutational status [18], cytogenetics [19,20] and cell surface protein expression [21,22] of AML cells may change during treatment in a large portion of patients (>40%). This has been attributed to the large heterogeneity of initial AML in which many different subclones may reside with different biological properties and mutational profiles [23]. During therapy, chemoresistant clones are selected and this clonal evolution results in a relapse consisting of cells with a common founder but for the remainder is divergent from the initial AML [24]. The cells that are selected to survive therapy should also be capable to re-initiate the leukemia, hence these cells have by definition a immature stem cell like phenotype. That is in line with our own findings that the relapse initiating cells present at diagnosis commonly resided in the CD34+/CD38- subpopulations [25]. So, many biological differences are crucial in the development of relapse, however, the precise biological background of AML relapse remains largely elusive. More detailed knowledge on the specific characteristics of the relapsed AML cells is required warranting further investigation.

In this exploratory study, we determined differences in genome wide gene expression of corresponding initial and relapse AML samples to find genes and gene expression profiles that play a role in development of relapse. The contribution of mutational shifts to differential gene expression was evaluated and molecules and pathways related to relapse development that were commonly affected in patients were identified.

## Material and Methods

### Patients

We studied initial and corresponding first relapse samples (N = 46) of 23 pediatric AML patients. Viably frozen bone marrow or peripheral blood samples from pediatric AML patients were provided by the Dutch Childhood Oncology Group (DCOG) and the ‘Berlin-Frankfurt-Münster’ AML Study Group (BFM-AML SG). Patients who suffered from recurrent disease within 2 years after initial diagnosis were selected. Clinical patient characteristics are summarized in Table 1.

**Table 1:** Clinical characteristics of the 23 childhood AML patients in this study at presentation and first relapse.

**Pair**	**Sex**	**FAB type**	**Cytogenetics**	**Blast% diagnosis**	**Blast% relapse**	**Time to relapse**	**Follow-up time**	**Dead**
1	M	M5	MLL t(10;11)	90	86	9.7	10.1	Yes
2	M	M2	Normal	93	82	3.7	9.2	Yes
3	M	M2	Loss Y	86	90	10.4	18	Yes
4	M	M4	t(6;9)	89	88	8.5	14.8	Yes
5	M	M5	Complex	88	84	8.9	9.2	Yes
6	M	M1	Unknown	91	86	14.8	19	Yes
7	M	M5	t(6;9)	93	89	5.7	12.3	Yes
8	M	M5	t(6;21)	96	82	15.8	26.3	Yes
9	F	M5	MLL t(9;11)	99	90	6.2	NA	No
10	M	M1	Unknown	82	93	11.4	14.9	Yes
11	M	M2	AML-ETO	91	94	58.4^1^	164.7	No
12	M	M2	AML-ETO	91	91	12.6	101.2	No
13	M	M2	AML-ETO	88	83	8.9	15.5	Yes
14	M	M0	Complex	93	91	2.6	20.8	No
15	M	M2	AML-ETO	95	97	7.7	18.5	Yes
16	F	M4	t(11;20)	90	89	9.8	19	Yes
17	M	M4	MLL t(10;11)	93	83	5.9	9.1	No
18	F	M2	Normal	84	81	15.3	16.3	Yes
19	M	M2	AML-ETO	95	93	14.1	53.7	No
20	M	M4	Del 9	89	96	8.7	20.6	No
21	M	M4	Normal	86	88	6.9	9.9	Yes
22	F	M4	MLL t(9;11)	90	95	8.7	21.5	Yes
23	M	M5	Del 7	93	92	14.4	215.7	Yes

Time to relapse and follow-up time are given in months

^1^patient 11 has follow-up time of > 2 years; Blast% after enrichment.

### Ethics statement

The initial diagnosis samples and their gene expression data were part of a previously published data set [26]. The study was approved by the individual Institutional Review Boards in the Netherlands (DCOG patients) and the Hannover Medical School Ethical Board (BFM patients) according to national law and regulations and written informed consent was obtained for all patients.

### Blast enrichment

Leukemic cells were isolated by sucrose density centrifugation and non-leukemic cells were eliminated as previously described [27]. All processed samples contained more than 80% leukemic cells, as determined morphologically using cytospins stained with May-Grünwald-Giemsa (Merck, Darmstadt, Germany). Subsequently, a minimum of 5*10^6^ leukemic cells were lysed in Trizol reagent (Invitrogen, Life Technologies, Breda, The Netherlands). Genomic DNA and total RNA were isolated according to manufacturer’s protocol.

### Cytogenetics

Cytogenetic aberrations were detected by standard chromosome-banding analysis, and screened for recurrent non-random genetic aberrations characteristic for AML, including MLL-rearrangements, inv(16), t(8;21) and t(15;17), using either RT-PCR and/or fluorescent in-situ hybridization (FISH).

### Mutation analyses

Samples were screened for hotspot mutations in *NPM1*, *CEPBA*, *FLT3*, *NRAS*, *KRAS*, *PTPN11*, *KIT* and *WT1* as previously described [18].

### Gene expression profiling and quality control

Integrity of total RNA was checked using the Agilent 2100 Bio-analyzer (Agilent, Santa Clara, USA). cDNA and biotinylated cRNA was synthesized and hybridized to the Affymetrix Human Genome U133 Plus 2.0 Array (Affymetrix, Santa Clara, USA) according to the manufacturer’s guidelines. Arrays with poor quality according to the manufacturer’s recommendations were excluded from further analysis.

### Data preprocessing

We applied the variance stabilization normalization procedure (VSN)[28] to remove background signal and normalize raw data across arrays. Log_2_ transformed expression values were calculated from perfect match (PM) probes only and summarized using a median polish method. The original and processed data from diagnosis and relapse samples have been deposited in the NCBI Gene Expression Omnibus (GEO; http://www.ncbi.nlm.nih.gov/geo) under GEO Series accession number GSE17855 [26] and GSE52891 respectively. (reviewer URL http://www.ncbi.nlm.nih.gov/geo/query/acc.cgi?token=qbszcmwwrdstxgv&acc=GSE52891).

### Statistical analysis

Probes with expression intensity below 30 were excluded from further analysis for previously mentioned reasons [29]. To identify differentially expressed probes in the VSN normalized expression values, we performed significance analysis of microarrays (SAM) [30]. We accepted a maximal false discovery rate (FDR) of 30% of cases with a confidence interval (CI) of 80%. Fold change expression differences of individual probe-sets between two classes (e.g. diagnosis and relapse) were calculated as ratios of geometric means, i.e. the anti-log of the arithmetic mean of the logs of probe-set intensities in each class. Differences between gene expression levels as measured by TaqMan were determined using Wilcoxon Signed Rank test. For survival analysis we used Kaplan-Meier analysis with log rank testing and Cox regression analysis to calculate the Hazard Ratio (HR) using SPSS version 20. Two sided p values below 0.05 were considered statistically significant.

### Software

R (version 2.10.1) and the Bioconductor packages affy, affyQC, simpleaffy, affyPLM, and VSN were used for quality control and preprocessing of raw data. Hierarchical clustering analysis with average linkage was performed using Cluster 3.0 [31] and visualized using Gene-E (http://www.broadinstitute.org/cancer/software/GENE-E/). Class comparison tests and class prediction tests were performed using the Biometric Research Branch Arraytools 4.2.1. Pathway analysis was performed using Ingenuity Pathway Analysis 7.5 software (Ingenuity Systems, Redwood City, CA, USA) based on the Ingenuity Pathways Knowledge Base. For details on our bio-informatical analyses, we refer to S1 Supplementary Information.

### Real-time quantitative PCR

The expression data for 5 selected genes (*TLE4*, *MALAT1*, *NUMB*, *EIF4E3* and *HIST1H1C*) were validated using real-time quantitative PCR in 7 independent AML patients of whom we had both initial and relapse samples available for these additional experiments. The GUS gene was used to normalize for differences in input cDNA. Pre-developed TaqMan Assays were used (Applied Biosystems, Foster City, CA, USA) and reactions were run on an ABI 7500 (Roche Diagnostics, Almere, The Netherlands) according to manufacturer’s description. Each sample was run in triplicate and the expression ratios were calculated using the ΔΔCT method after prior validation of the method for each target. Bone marrow from 2 healthy individuals was used as a reference for relative gene expression levels.

## Results

Genome wide gene expression profiling was performed for initial leukemia and corresponding relapse samples (N = 46) of 23 pediatric AML patients. The majority (19 out of 23 patients, 79%) of the patient group was male, the median age at presentation was 13.2 years and patients belonged to standard, intermediate and poor cytogenetic risk groups. Seventeen patients (74%) died after relapse. Overall patient characteristics are summarized in Table 1 and described more in detail in S1 Table.

We screened for mutations in a selected panel of genes (*NPM1*, *CEPBA*, *FLT3*, *NRAS*, *KRAS*, *PTPN11*, *KIT* and *WT1*) that were previously shown to associate with outcome in AML [11,18] in both the initial and corresponding relapse AML samples; results are shown in S1 Table. Analogous to our previous observations [18] both gains and losses of mutations between initial and relapse AML samples occurred in 8 out of 23 (35%) patients. In 22 out of 23 patients cytogenetic analysis was successfully performed on initial diagnosis samples according to standard clinical practice. Cytogenetic analysis at relapse is not part of routine diagnostics at the time of relapse treatment and was therefore not available for all patients. Cytogenetic data was available for both initial and relapsed samples in 10 cases. In these paired samples, the major chromosomal rearrangements remained stable compared to diagnosis, although additional cytogenetic aberrations were observed (S1 Table).

### Comparison of initial with relapse expression profiles

**Fig 1:**
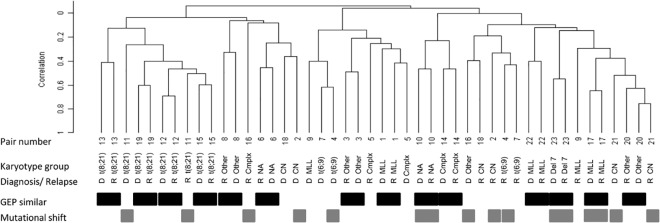
Hierarchical clustering dendrogram of paired initial and relapse AML samples. Similarity of GEP: Black bars indicate paired samples with GEP that correlate according to hierarchical cluster analysis. Grey bars indicate paired samples with mutational shifts between initial and relapse AML samples.

To evaluate how well the relapse samples of individual patients resembled the corresponding initial sample, unsupervised hierarchically clustering was performed using a ‘one minus’ centered correlation and average linkage. For this analysis GEP data of all 46 patient samples were preprocessed and probe sets were selected with a 1.5 fold variation in at least 2 samples. Fig 1 depicts how samples cluster together. The correlation between the initial and relapse samples with a patient varied between r_s_ = 0.15 and r_s_ = 0.75 (as illustrated by the difference in length of the branches in Fig 1). In 11 out of 23 patients (48%) the GEP of the relapse sample did not cluster together with the GEP of the initial sample. In 9 out of these 11 patients, the GEP of the initial sample was observed to cluster more than one branch away from the corresponding relapse GEP, indicating dissimilar GEP between the two disease stages for these individual patients. Remarkably, 5 out of these 9 paired samples (55%) also showed a shift in mutational status between the initial leukemia and relapse sample (Fig 1). Four out of the 9 patients showed a lack of correlation between initial leukemia and relapse GEP, despite a stable mutational status. It cannot be excluded that mutations in other genes than the currently investigated genes are involved and have changed, which was not detected here.

In 12 out of the 23 patients, initial and relapse samples clustered together, indicating similar GEP at both disease stages. In 3 out of these 12 patients (25%) mutational shifts of the analyzed genes were observed. In our previous studies we found a relation between mutational shifts and a shorter time to relapse (TTR) [18,32]. Interestingly, this was corroborated in the current study in which patients with a GEP shift at relapse had a shorter TTR (median 8.7 vs 10.4 months), although not significant (*P* = 0.43), which may be due to the outlier relapse time of patient 11 (58.4 months). This mutational shift might imply a second malignancy but that cannot be concluded based on our current data but would require deep sequencing analysis. Despite the small patient number, we did also confirm our previous finding of a significant worse OS in patients that show mutational shifts (log-rank: *P* = 0.04, Hazard ratio: 2.8, *P* = 0.05).

### Differentially expressed genes in initial and relapse pediatric AML samples

**Fig 2:**
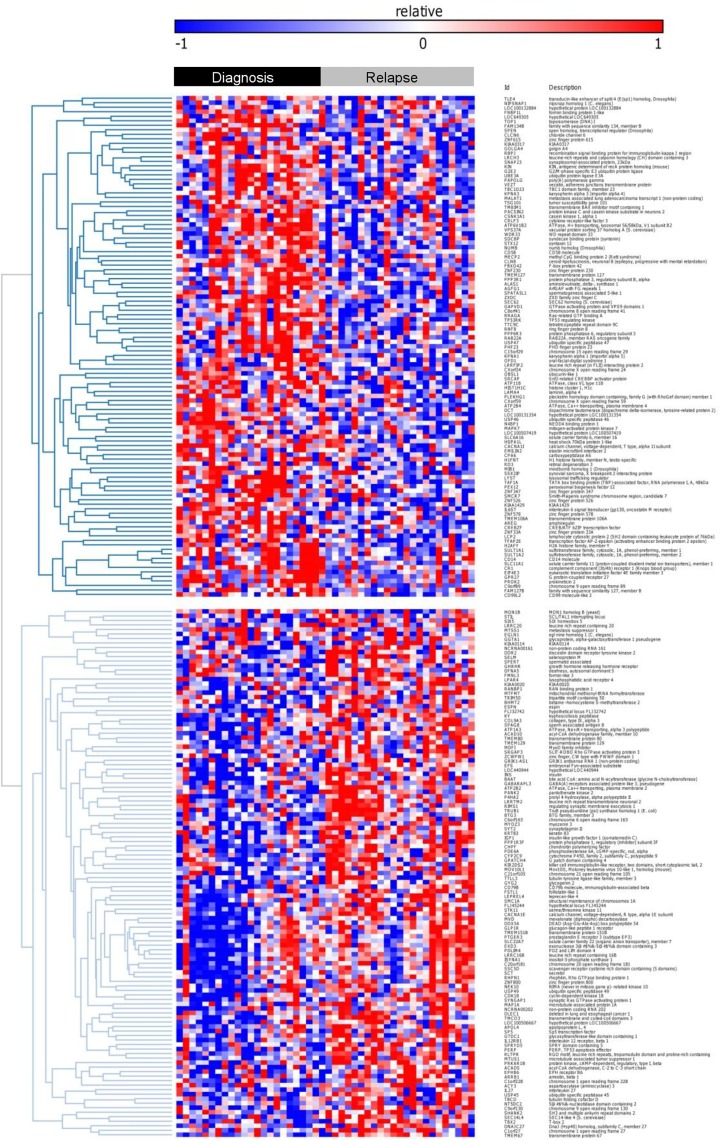
Heat map of probe-sets that distinguish initial from relapse AML samples. The black top bar indicates initial AML samples and the top gray bar represents relapse AML samples.

To first assess differential expression of individual genes that discriminate between initial and relapse samples in general, irrespective of patient specific characteristics, we compared GEP of the 23 initial with the 23 relapse AML samples (comparing the class of diagnosis to the class of relapse expression profiles). Even with a low stringency false discovery rate of 0.3, SAM analysis yielded only few individual genes that were discriminative between initial and relapse samples (S1 Fig). Stratification according to cytogenetic subgroups did not improve these results (data not shown). With the aim to find relapse specific gene expression signature rather than individual genes that discriminate relapse from initial samples, we developed a multi-gene classifier using BRB Arraytools. The classifier consisted of 306 probes (228 known genes) that were discriminative between initial and relapse AML samples with a high sensitivity (70–83% depending on the cross validation method applied, *P*_306th probe_ = 0.005, S3 Table, Fig 2). Hence, there seems to be a set of genes that defines relapsed AML.

**Fig 3:**
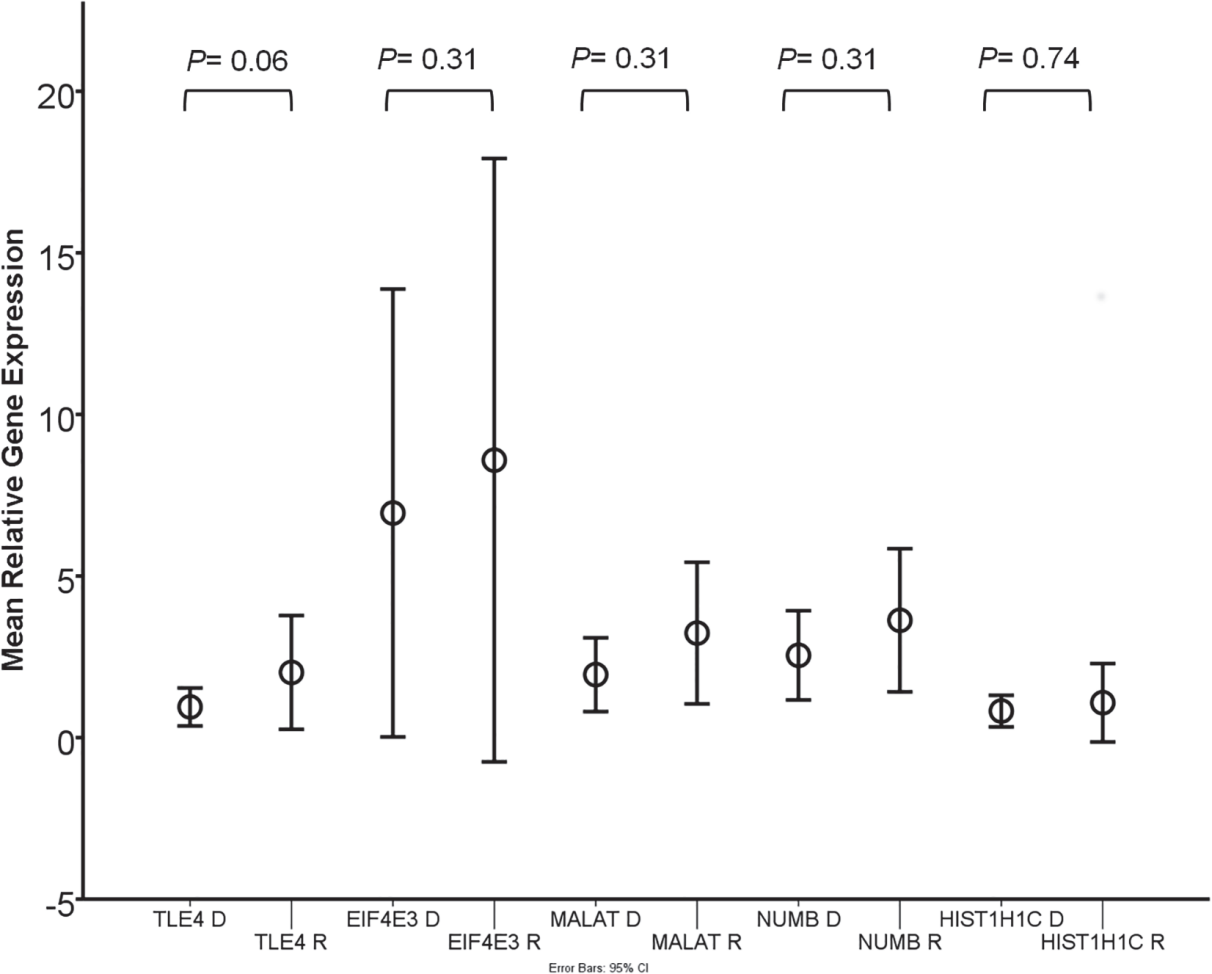
TLE4, MALAT1, NUMB, EIF4E3 and HIST1H1C relative mRNA gene expression levels in an independent set of 7 paired diagnosis and relapse samples.

To confirm these findings, we validated the differential expression of *TLE4*, *MALAT1*, *NUMB*, *EIF4E3 HIST1H1C* by Taqman RT-PCR in 7 independent sets of paired initial and relapse AML samples (n = 14, Fig 3). In these samples, the expression of *TLE4* was increased in 5 out of 7 cases (*P* = 0.06) and in *MALAT1*, *NUMB* and EIF4E3 in 4 out 7 cases (*P* = 0.31). The expected relative downregulation of the expression of *HIST1H1C* in relapse samples compared to diagnosis samples was found in 4 out of 7 cases (but not statistically significant: *P = 0*.*74)*.

Since AML patients are genetically heterogeneous [8,33] we hypothesized that relevant differences are better studied by focusing on individual patients and their patient specific differences between diagnosis and relapse. To this end, we individually compared gene expression profiles of the initial samples with the corresponding relapse sample of each patient (n = 23). This allows taking patient specific differences between diagnosis and relapse samples into account. Differential gene expression was determined for each individual patient by selecting genes that minimally had a two-fold change in expression level between diagnosis and relapse. In all 23 patients, more genes were up-regulated (median 422, range 40–1743 genes) than down-regulated (median 277, range 53–1326 genes) at relapse. Ingenuity pathway analysis was performed for these differentially expressed genes for each patient. The three most commonly affected pathways involved inflammatory disease related networks (21/23 patients), cell movement and proliferation networks (15/23 patients) and chromatin disorder networks (13/23 patients).

In addition, Inqenuity pathway analysis for each individual patient predicted which transcription factors (TF) could be responsible for the observed gene expression patterns based on experimentally observed relationships between TF and gene expression. Prediction of TF involvement in gene expression patterns was performed based on a p-value of overlap <0.05 between our probe lists and the Ingenuity TF related gene expression database. From the 23 lists of TF for each patient, we determined which TF genes were commonly involved between the 23 patients (S4 Table). The top 5 TF and their associated target genes that were differentially expressed with robust p-values of overlap in our data in the majority of patients are listed in Table 2. The expression changes between diagnosis and relapse of these TF target genes were both mixed in up- and down-regulated among the different pairs, except for CDKN1A, which was up-regulated in relapse in all 6 pairs.

**Table 2:** Top list of transcription factors that regulate differentially expressed genes in paired diagnosis and relapsed samples, ranked by incidence.

**Transcription Factor**	**Median** ***P*** **value (range)**	**Percentage of patients**	**Top target molecules in data set (portion of patients) **
*CEBPA*	2.6 x10^-5^	87	MPO(12/20), S100A9(12/20), ID2 (11/20), SOD2 (11/20), ANXA1 (11/20), CXCR4 (10/20), BTG1 (10/20), H1FX (10/20)
	(2.0E-03–1.5E-09)		
*GFI1*	8.2E-03	69	IL8 (14/16), ELANE(10/16), AZU1(9/16), RB1 (8/16), CDKN1A (6/16), SERPINA1(6/16)
	(4.4E-02–1.1E-05)		
*SATB1*	4.33E-03	69	HBB (12/16), HSPAA90 (11/16), RGS1 (11/16), CLEC2B (8/16)
	(2.7E-03–4.6E-05)		
*KLF2*	7.1E-03	65	IL8 (13/15), CCL23(12/15), CCL4(8/15),IL1B(7/15),PTGS2(7/15),SELL(7/15)
	(2.3E-02–1.2E-05)		
*TBP*	3.1E-03	61	TNFAIP3 (14/14), NFKBIA (10/14), IER3 (9/14), BCL2 (5/14), HLA-A (5/14)
	(4.7E-02–5.9E-05)		

**Fig 4:**
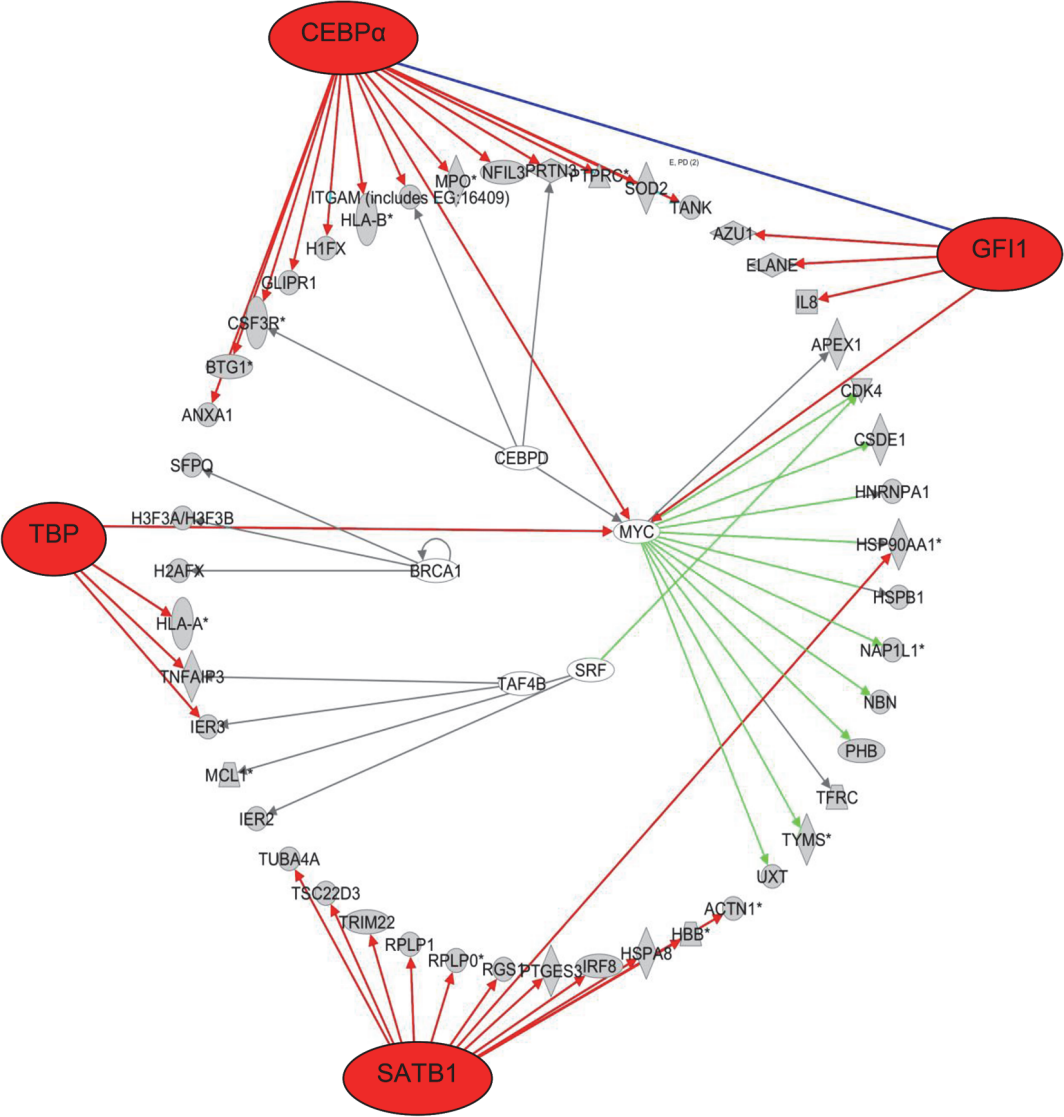
Transcription network visualization plot for patient 3. Transcription network plot showing transcription factors (outer ring/ inner ring) that are predicted responsible for differential expression of shown target molecules (middle ring) between diagnosis and relapse of patient 3. A few transcription factors (*CEBPA*, *GFI1*, *SATB1* and *TBP*) are responsible for the major changes in the differentially expressed target molecules.

In particular, *CEBPA*, *GFI1* and *SATB1* that were affected in 20, 16 and 15 out of 23 patients, respectively. Either one of these transcription factors was predicted to be involved in the differential gene expression profiles of all 23 patients. Network visualization plots show which transcription factors were involved and their target molecules that were at least 2 fold differentially expressed for individual patients (S2 Fig). For example, in patient 3, differential gene expression of the target molecules was predicted to result primarily from *CEBPA*, *GFI1*, *SATB1* and *TBP1* activation/ inhibition (Fig 4). In addition, other transcription factors, such as *CEBPD*, *BCRA1*, *MYC*, SRF, and *TAF4B* were also significantly involved in this patient.

The above described results implicate molecules and pathways that are involved in epigenetics. In concordance with these observations, differential expression of histone variant genes was observed in all analyses (Fig 2, S1 Fig) and in the differential gene expression profiles of individual patients. In addition, regression analysis showed that the expression of individual histones variants correlated strongly with the expression of other cell cycle independent histone variants (S2 Table).

## Discussion

Improvements in OS for AML are likely to come from personalized targeted treatment approaches that aim to eradicate persistent leukemia. With the aim to elucidate factors that characterize pediatric relapsed AML, we performed a genome wide gene expression study on both initial and relapsed AML samples from 23 patients.

In 11 out of 23 (~48%) initial and relapsed AML samples the GEP did not culminate in the same cluster (Fig 1) and hence differed significantly. Notwithstanding this fact, also in cases where the GEP of initial and relapse samples did cluster together, differentially expressed genes could be identified. Consequently, genes were identified in several biological pathways that are relevant for relapse development. In addition, their underlying transcription factors could be recognized that were changed between initial diagnosis and relapse within each patient. In the majority of patients (86%), *CEBPA* transcription factor related gene expression had changed at relapse. *CEBP* transcription factors play a crucial role in hematopoiesis [34]; it is indispensable for differentiation of myeloid progenitors along the granulocytic lineage [35,36]. Regulation of lineage-specific gene expression is known to occur via direct interaction with the basal transcriptional apparatus (*TBF*/*TFIIB*), but also via interaction with the *SWI*/*SNF* chromatin remodeling complex that modulates gene expression in an epigenetic manner [37]. Different types of *CEBPA* mutations are implicated in leukemogenesis [38,39] and confer a good prognosis subgroup in both adult and pediatric cytogenetically normal AML [40,41]. In our selected relapsed AML patient group, only one patient showed a single *CEBPA* mutation. It has also been shown that the normal function of *CEBPA* in hematopoietic differentiation can be altered for example via oncogenic lesions [42], fusion proteins [43,44] or epigenetic alterations [45]. This may explain why in our study, genes that are regulated by *CEBPA* often showed an altered expression, while *CEBPA* expression itself was not significantly changed. For example, the cell surface glycoprotein CD9 is down regulated in bi-allelic mutated *CEBPA*[46] and its expression was also down regulated in our relapsed AML samples. Commonly, CD9 has a low expression on CD33 positive myeloid progenitors and plays a role in the regulation of cell differentiation, growth and motility [47]. Likewise, other genes are suggestive of a more primitive phenotype of the relapse cells such as the lower expression in relapse samples when compared to their individual initial AML samples of differentiation markers *CD14*, *CD58* and also *CXCR4*, which is usually low in immature cells. (S3 Table). In addition, these results indicate a *CEBPA* transcription factor related change in differentiation status of leukemic cell populations during disease progression. This may result in more primitive characteristics of the leukemic blast compartment at relapse when compared to presentation. Moreover, this implies a potential benefit for relapsed AML patients to receive therapy, that restores *CEBPA* related gene expression or normal differentiation, e.g. by gene therapy, nanoparticle based delivery of functional *CEBPA* or small molecule modulators of *CEBPA* or its downstream signaling [48].

Another transcription factor of which the target gene expression levels were commonly (22 out of 23 patients) deregulated between initial and relapsed AML samples, was *SATB1*. This transcription factor is thought to be a key epigenetic modifier that links higher-order chromatin organization with gene regulation [49]. It regulates for example the expression of globin gene cluster [50], many cytokines [51], plays a role in hematopoietic differentiation [50,52–54] and is implicated in a variety of cancers and cancer progression [55,56].

We validated the differential expression of five genes (*TLE4*, *MALAT1*, *NUMB*, *EIF4E3* and *HIST1H1C)* of our 306 probe set classifier and class comparison tests. The Groucho corepressor *TLE4* may have a role as a tumor suppressor gene in the subgroup of AML-ETO patients, where its loss may promote survival and proliferation of leukemic cells [57]. Knockout studies in mice suggest an essential role for *TLE4* in hematopoiesis [58]. A causal role of increased *TLE4* expression at relapse as observed in our study remains to be elucidated. High expression levels of the long non coding RNA *MALAT1* are associated with aggressive proliferation, metastasis and recurrence in a variety of cancers [59–61]. The observed down regulation of histone variant gene expression levels in a subset of patients (e.g. *HIST1H1C)* may point at altered epigenetics in relapsed AML [62]. Histone variants may play a role in hematopoietic differentiation [63] and the expression of specific variants may be associated with leukemia [64]. Their functions are various, e.g. in the response to growth factors [65] or DNA damage response related apoptosis [66]. The expression of histone variants may be influenced by drug exposure [67] and may play a role in the tolerance to toxins [68]. These properties of histones have been exploited in the development on recombinant human histone 1.3 (rhH1.3) as a cancer therapeutic agent, which was shown to induce apoptosis in leukemia cells by rupture of the plasma membrane. This drug was applied in a phase II trial for adult relapsed AML [69].

The observed changes in gene expression from diagnosis to relapse and the patterns of clustering may be explained by the emergence at relapse of minor clones with different genetic make-up when compared to bulk of AML cells at diagnosis [24]. We have shown earlier that such minor clonal populations can be isolated retrospectively from diagnosis samples by FACS sorting. Besides a relapse specific mutational status, these cells expressed immunophenotypic markers that were specific for the bulk of leukemic cells at relapse [25]. Our current study is limited in the number of genes in which the presence of mutations was tested. In addition to changes that are causal in relapse development, also circumstantial differences may occur during the selection of a specific clone at relapse. Therefore, common relapse specific mutations and relapse specific gene expression profiles should be assessed in depth by next generation RNA sequencing of minimal residual disease cells that are likely to harbor the cells that initiate relapse.

In conclusion, we show a variable differential gene expression between initial and relapsed AML sample of individual patients. One group of patients shows a tumor evolution by which resemblance between initial diagnosis and relapse gene expression profiles is lost (11 out 23 patients). The remainder of patients showed a more similar initial diagnosis and relapse gene expression profile, however, relapsed AML samples still have specific GEP that discriminate it from initial AML. The multiple pathways that were affected in individual patients may result from an epigenetic deregulation as suggested by observed the *CEBP* and *SATB1* transcription factor related differential gene expression and the diminished expression of e.g. histone variants at relapse. Our findings are in line with the current notion that the eradication of cells with stem cell like properties is essential for the prevention and probably also the treatment of relapse [70]. Therefore, the currently available biological characteristics of relapsed AML should be exploited in the application and development of novel strategies that may prevent relapse or improve salvage therapies.

## Supporting Information

S1 Fig Heatmap depicting SAM analysis results. Twenty-three probe sets were discriminative between initial (bleu bar) and relapse (yellow bar) samples.
(PDF)

S2 Fig Transcription network visualization plot for all 23 patients separately. Transcription network plot showing transcription factors (outer ring/ inner ring) that are predicted responsible for differential expression of shown target molecules (middle ring) between diagnosis and relapse. A few transcription factors (*CEBPA*, *GFI1*, *SATB1* and *TBP*) are responsible for the major changes in the differentially expressed target molecules.
(PDF)

S1 Supplementary Information Bioinformatical analysis of paired diagnosis and relapse pediatric AML gene expression micro array data. (DOC)

S1 Table Mutation analysis of paired primary and relapse AML samples. (PDF)

S2 Table List of probesets that are co-expressed with HIST1H1C expression. (PDF)

S3 Table List of 306 probe-sets of the classifier that discrminates between initial and relapse AML samples, ranked according to P-value. (PDF)

S4 Table Ingenuity transcription factor analysis output. (PDF)
