# Supplementary figures and images for: Gene Expression Profiles Associated with Pediatric Relapsed AML
Source: PLoS One. 2015 Apr 7;10(4):e0121730. doi: 10.1371/journal.pone.0121730 (PMC4388534; doi:10.1371/journal.pone.0121730)

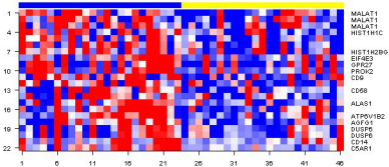

Supplement: S1 Fig — Twenty-three probe sets were discriminative between initial (bleu bar) and relapse (yellow bar) samples. (PDF) [file pone.0121730.s001.pdf]
