# Supplementary material for: Gene Expression Profiles Associated with Pediatric Relapsed AML
Source: PLoS One. 2015 Apr 7;10(4):e0121730. doi: 10.1371/journal.pone.0121730 (PMC4388534; doi:10.1371/journal.pone.0121730)

Transcription factor analysis visualization plot of patient 1

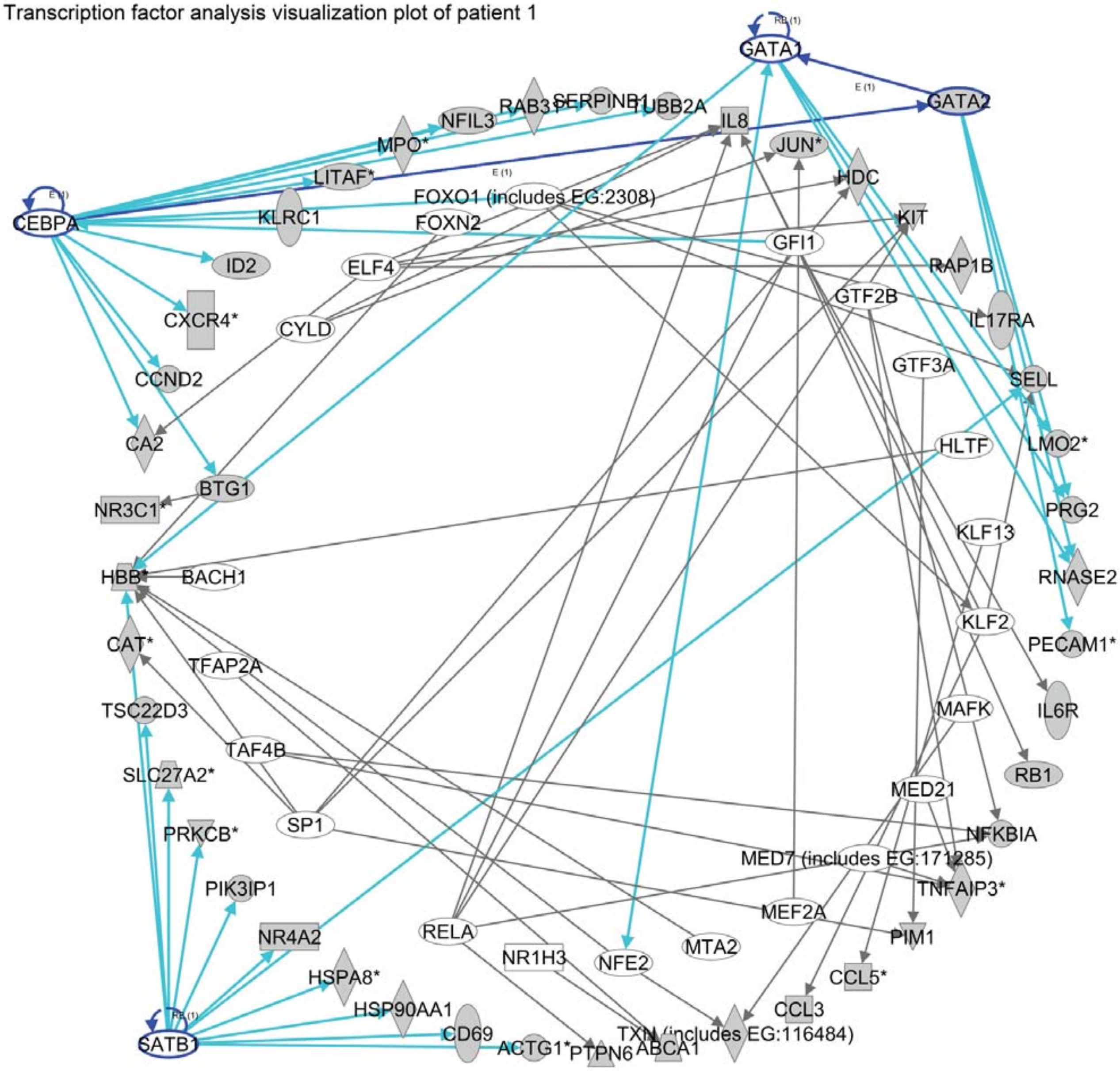

Transcription factor analysis visualization plot of patient 2

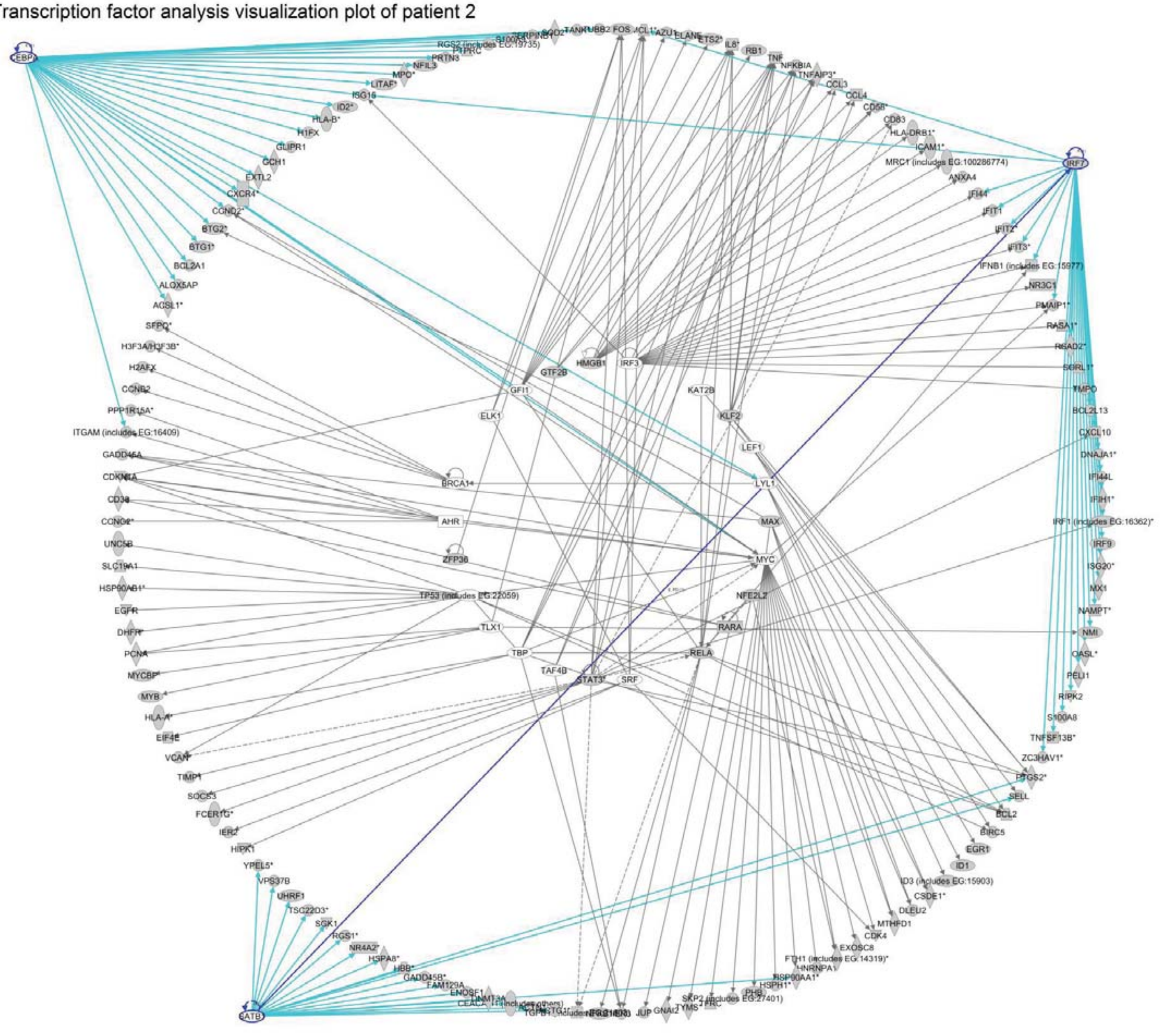

Transcription factor analysis visualization plot of patient 3

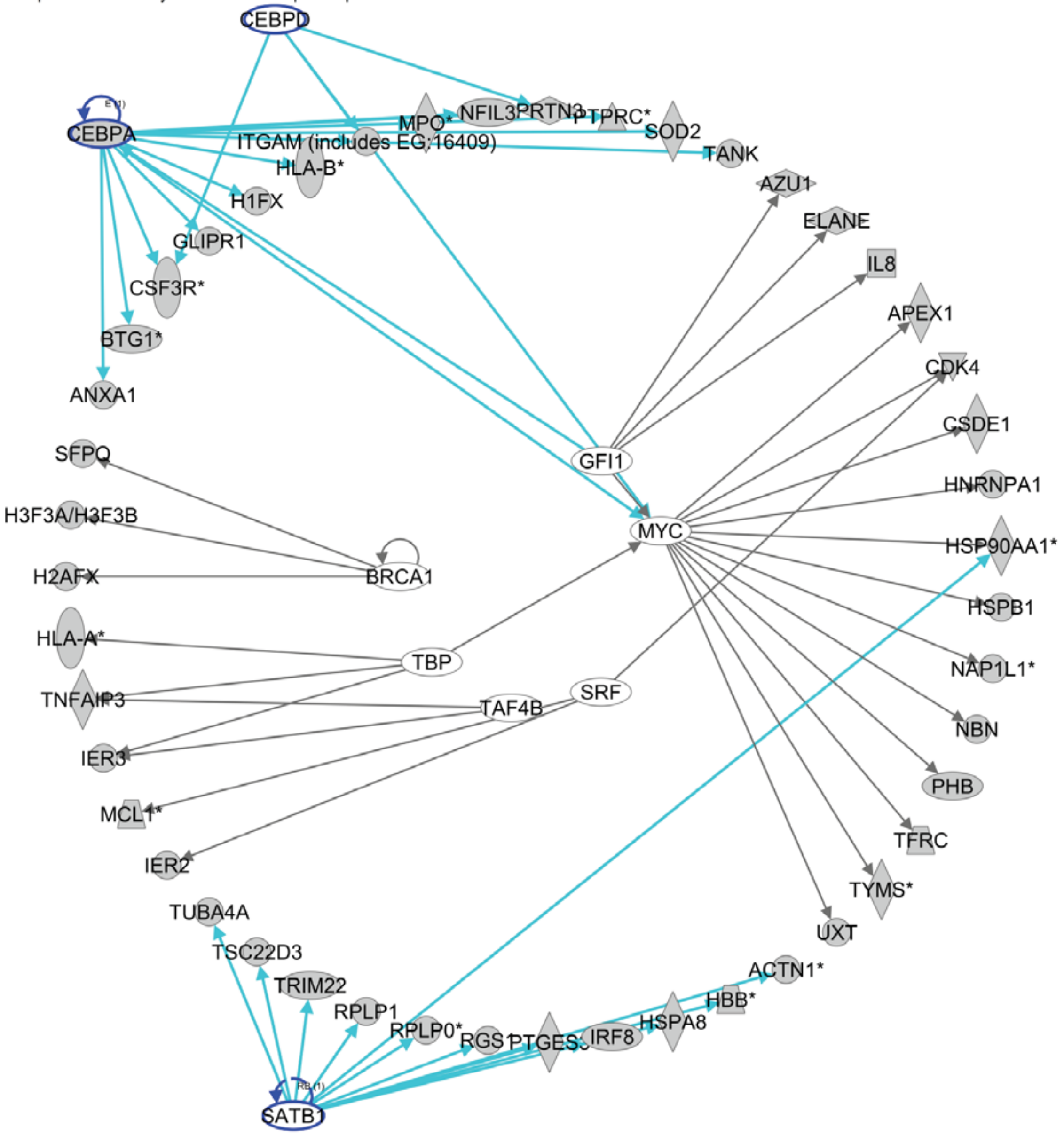

Transcription factor analysis visualization plot of patient 4

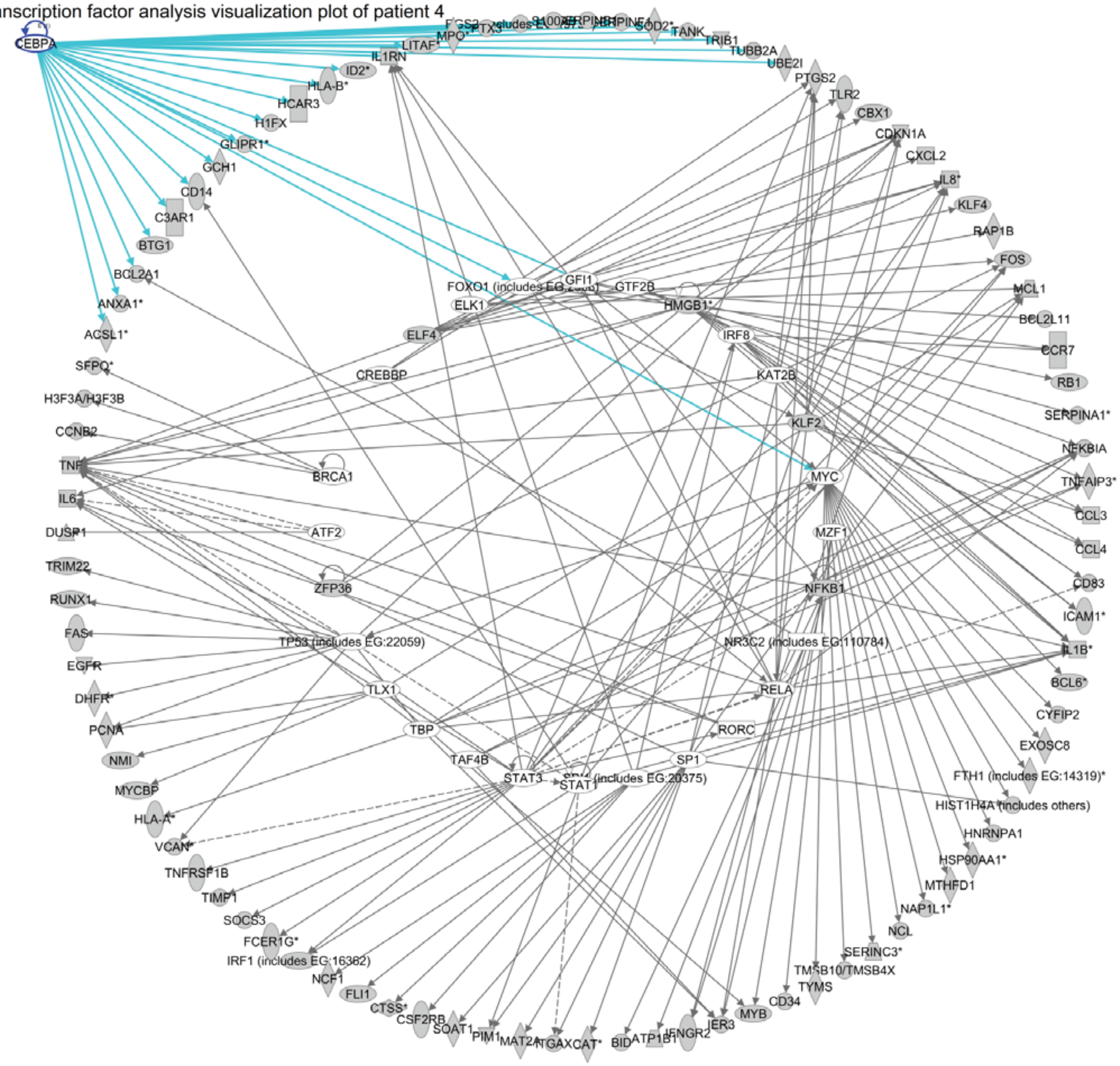

Transcription factor analysis visualization plot of patient 5

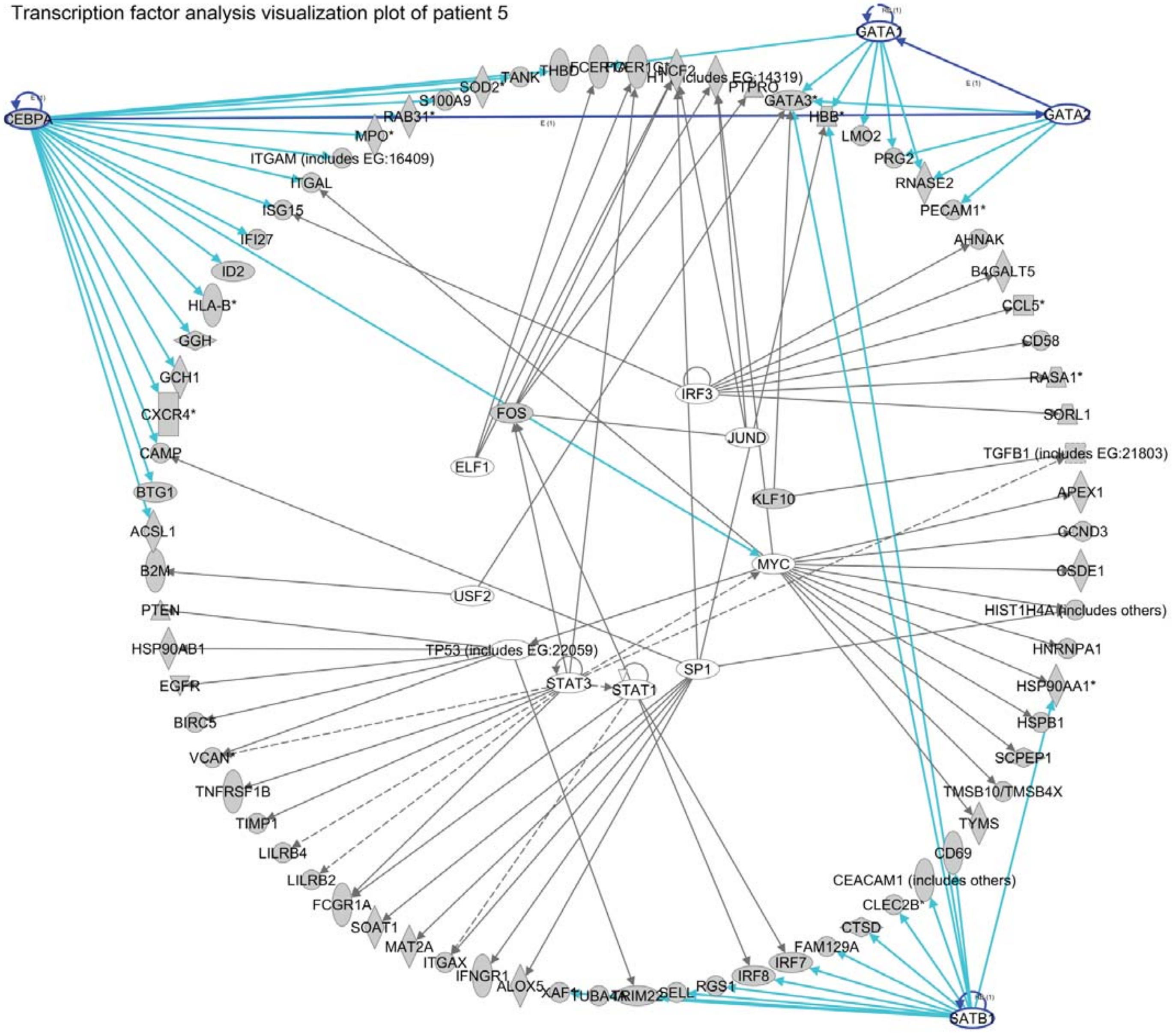

Transcription factor analysis visualization plot of patient 6

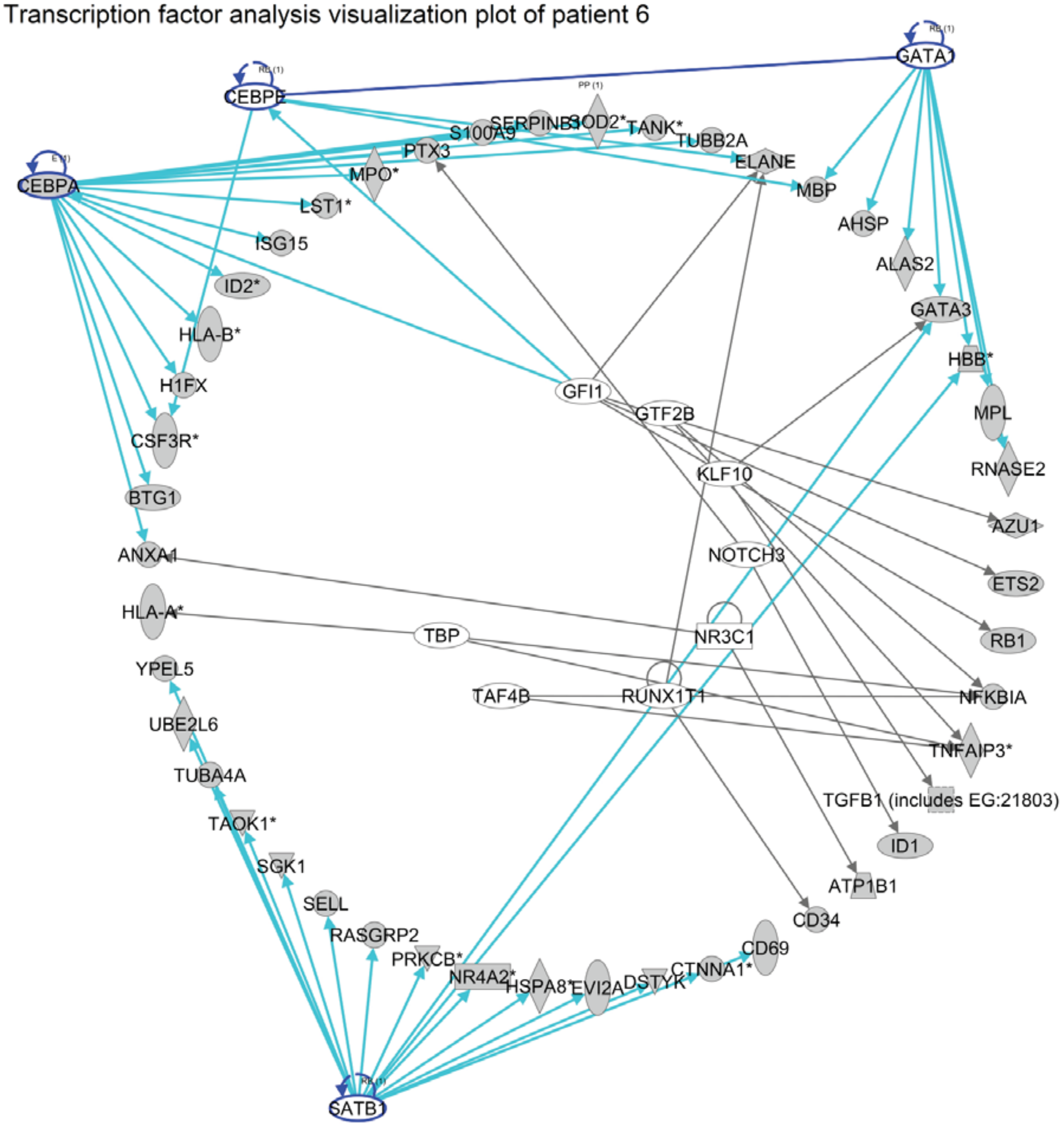

Transcription factor analysis visualization plot of patient 7

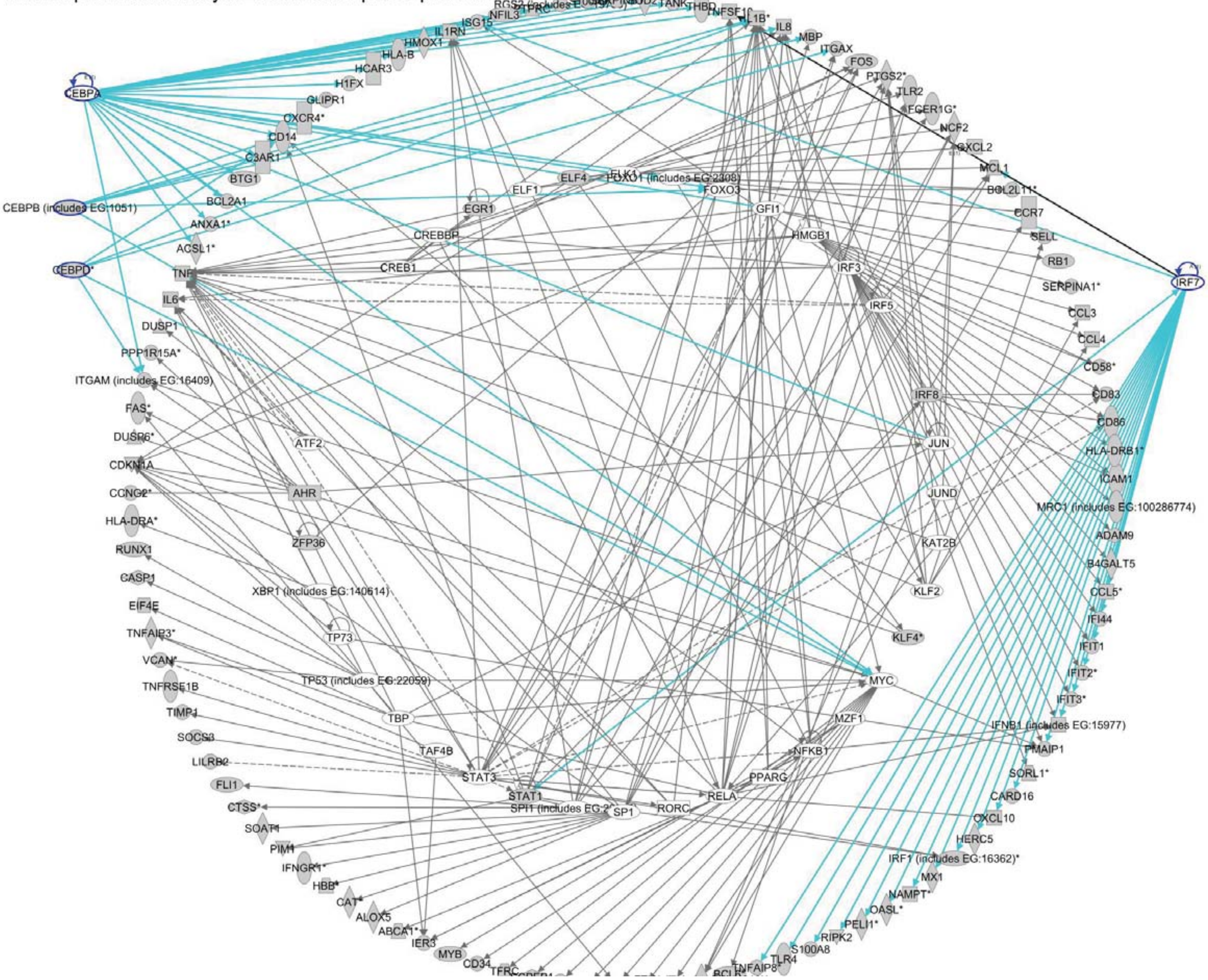

Transcription factor analysis visualization plot of patient 8

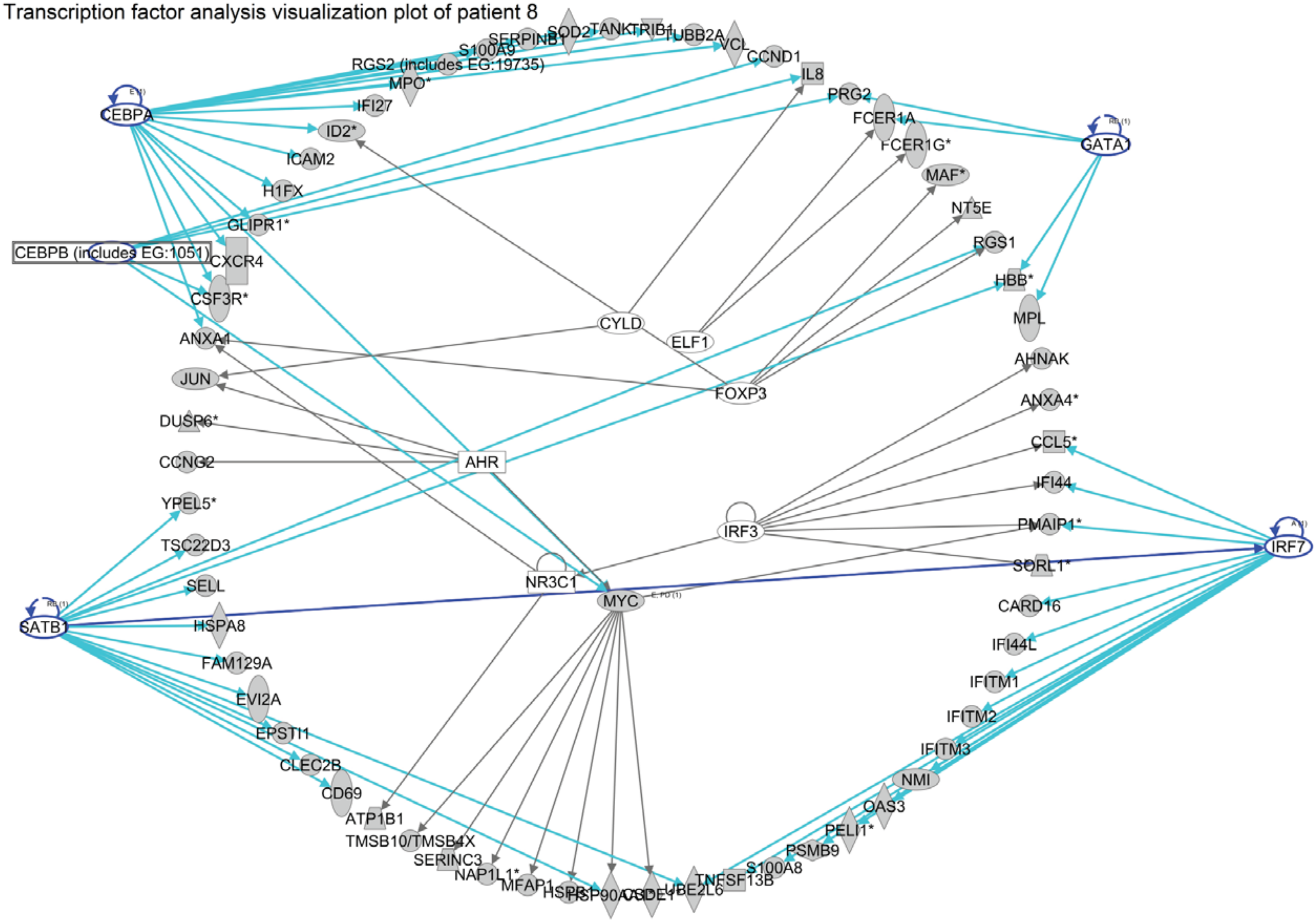

Transcription factor analysis visualization plot of patient 9

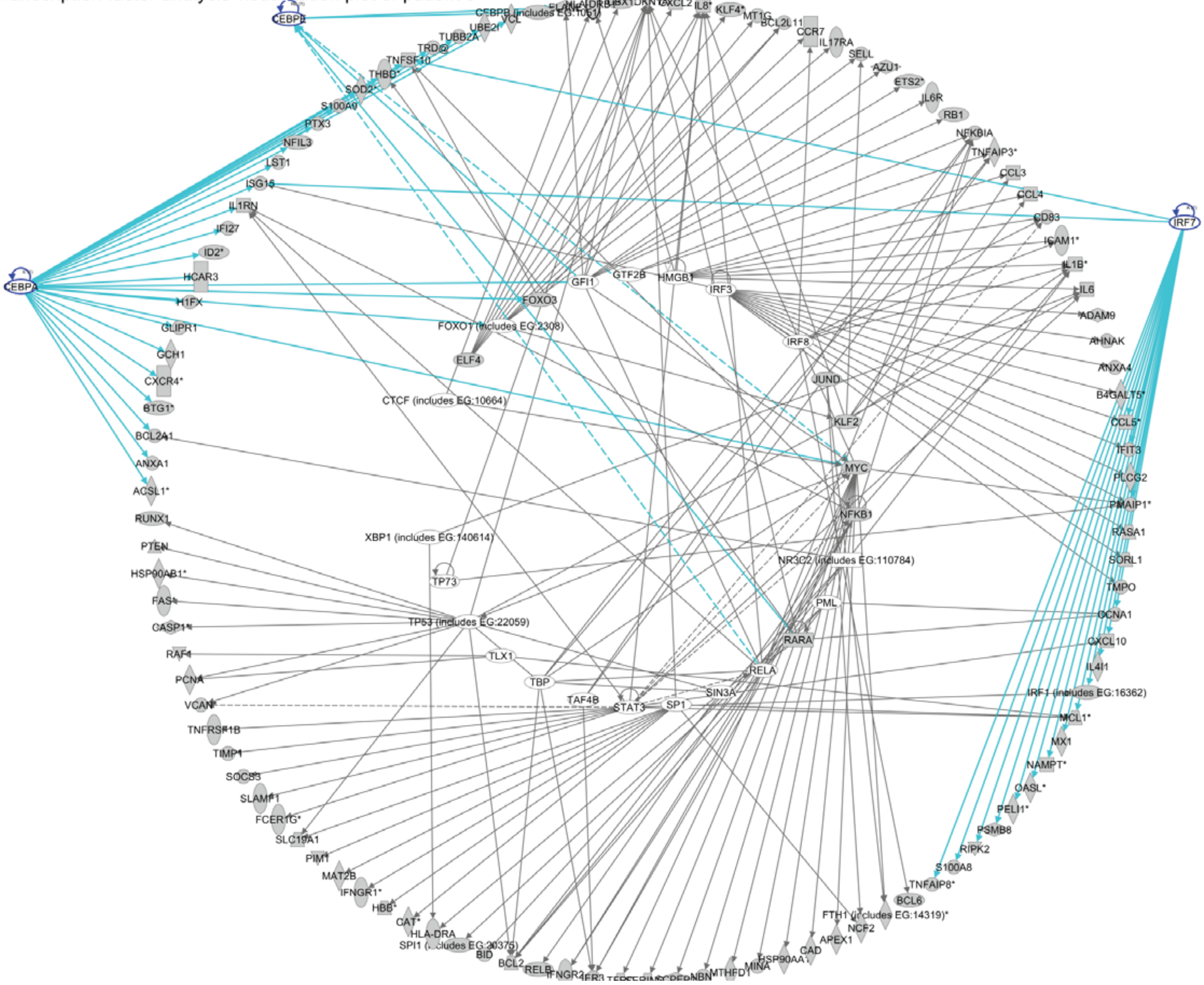

Transcription factor analysis visualization plot of patient 10

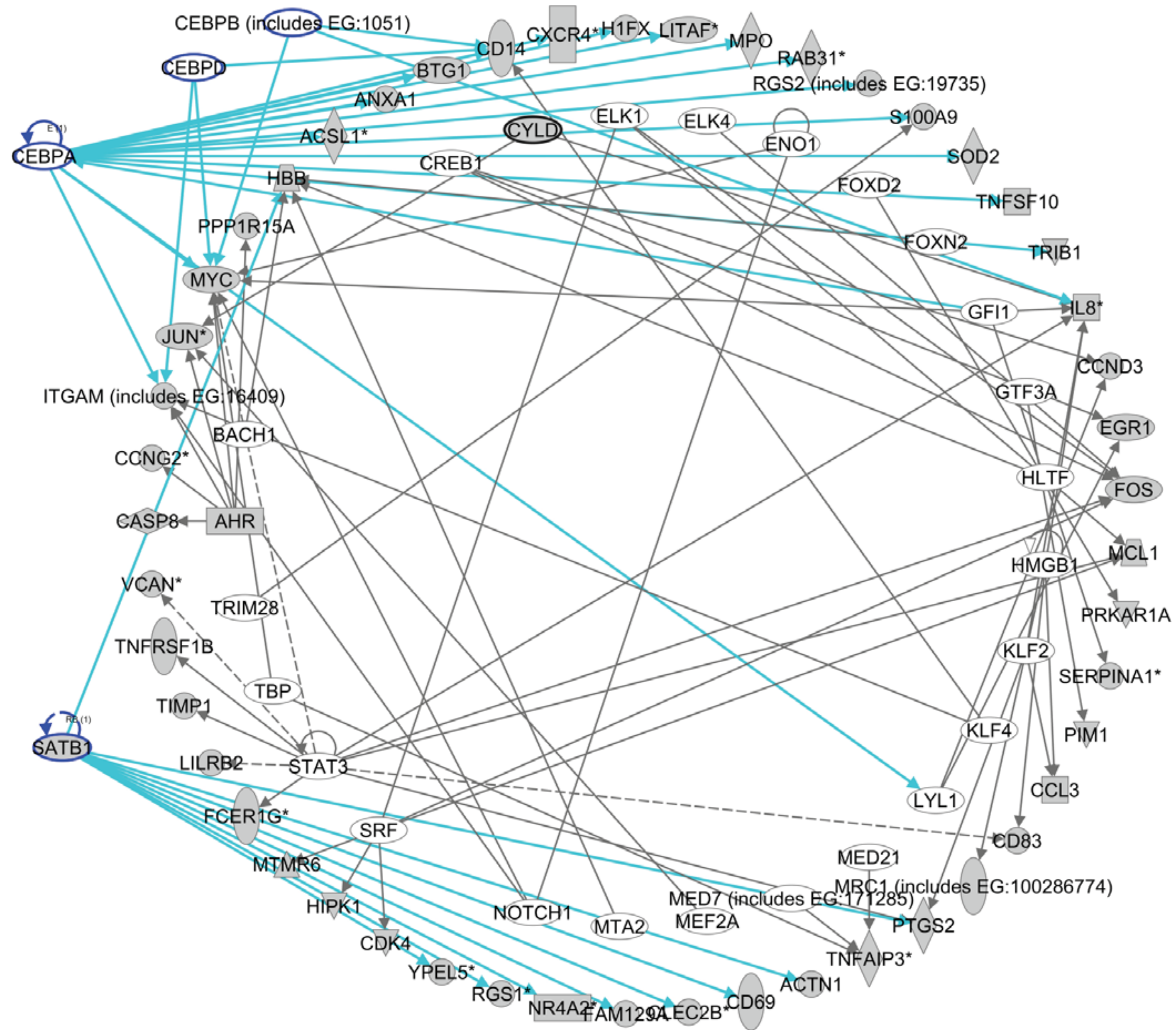

Transcription factor analysis visualization plot of patient 11

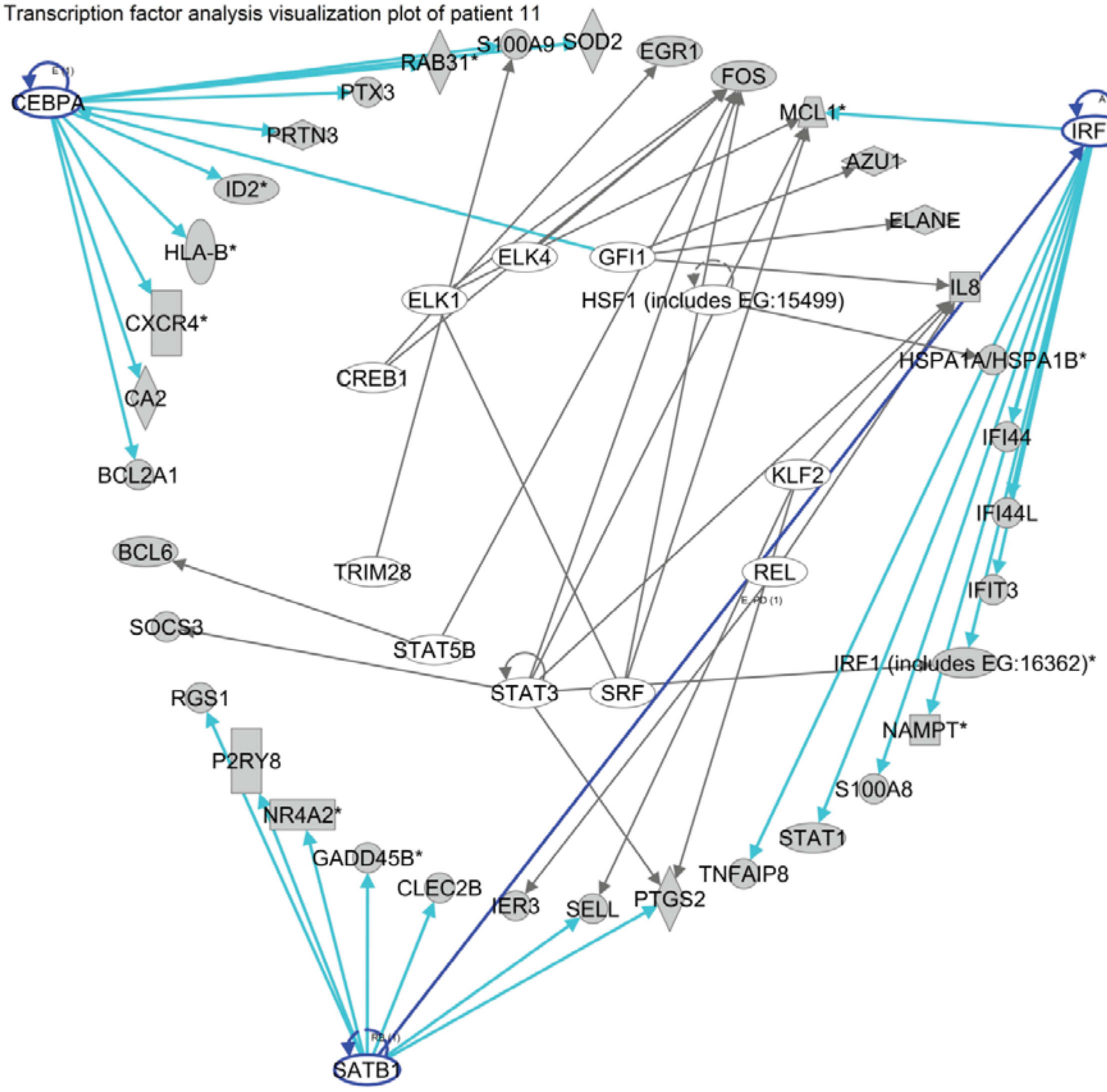

Transcription factor analysis visualization plot of patient 12

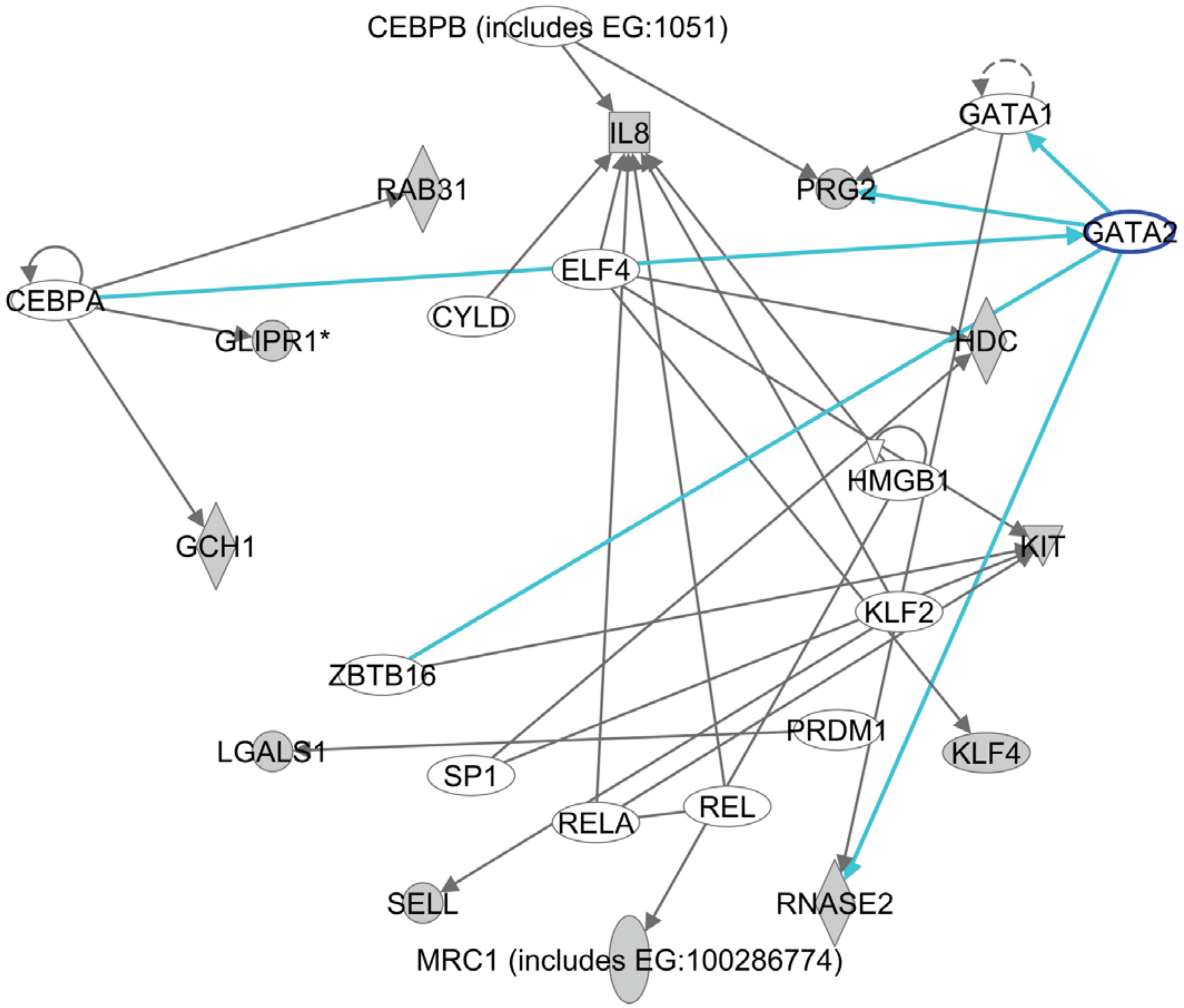

Transcription factor analysis visualization plot of patient 13

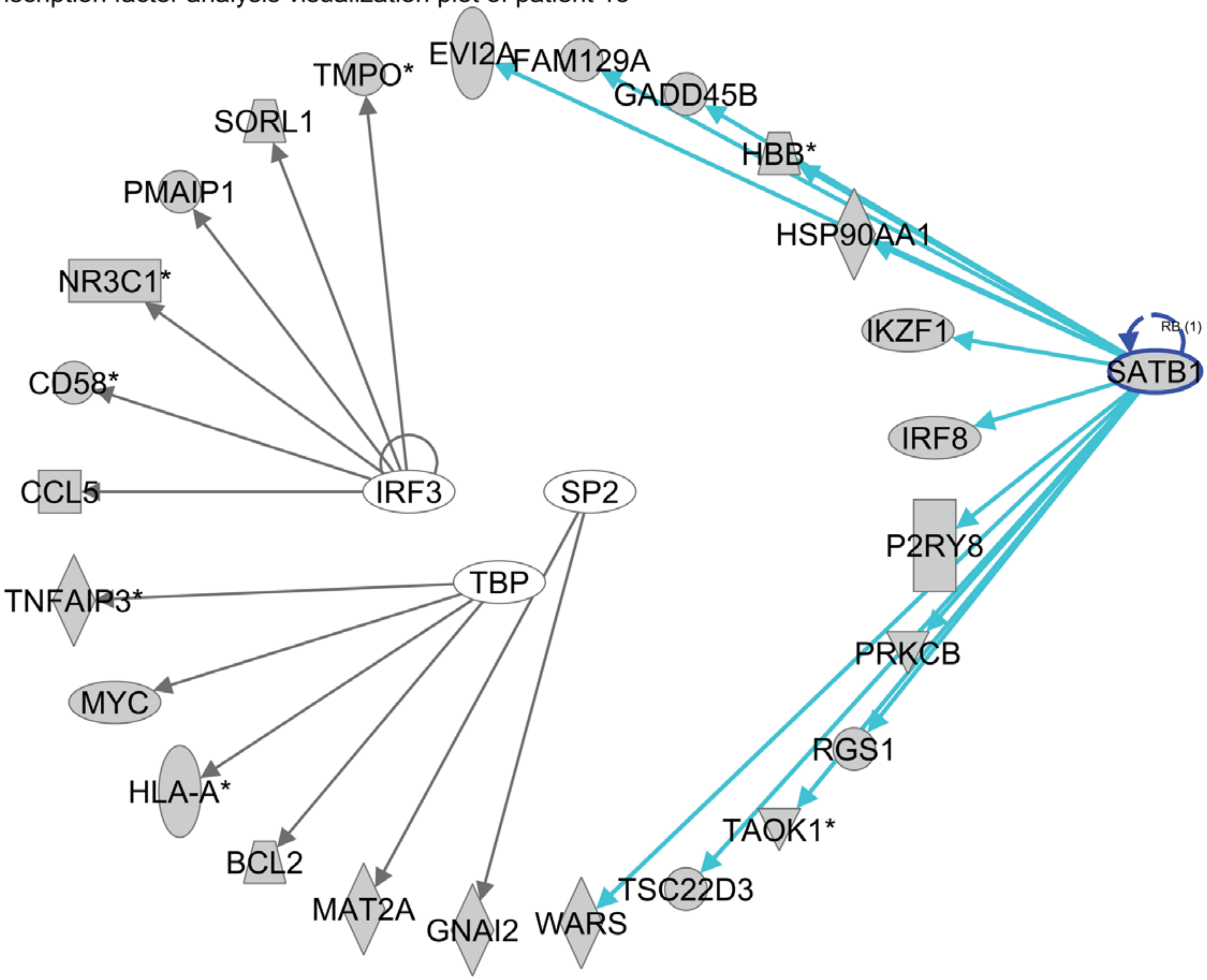

Transcription factor analysis visualization plot of patient 14

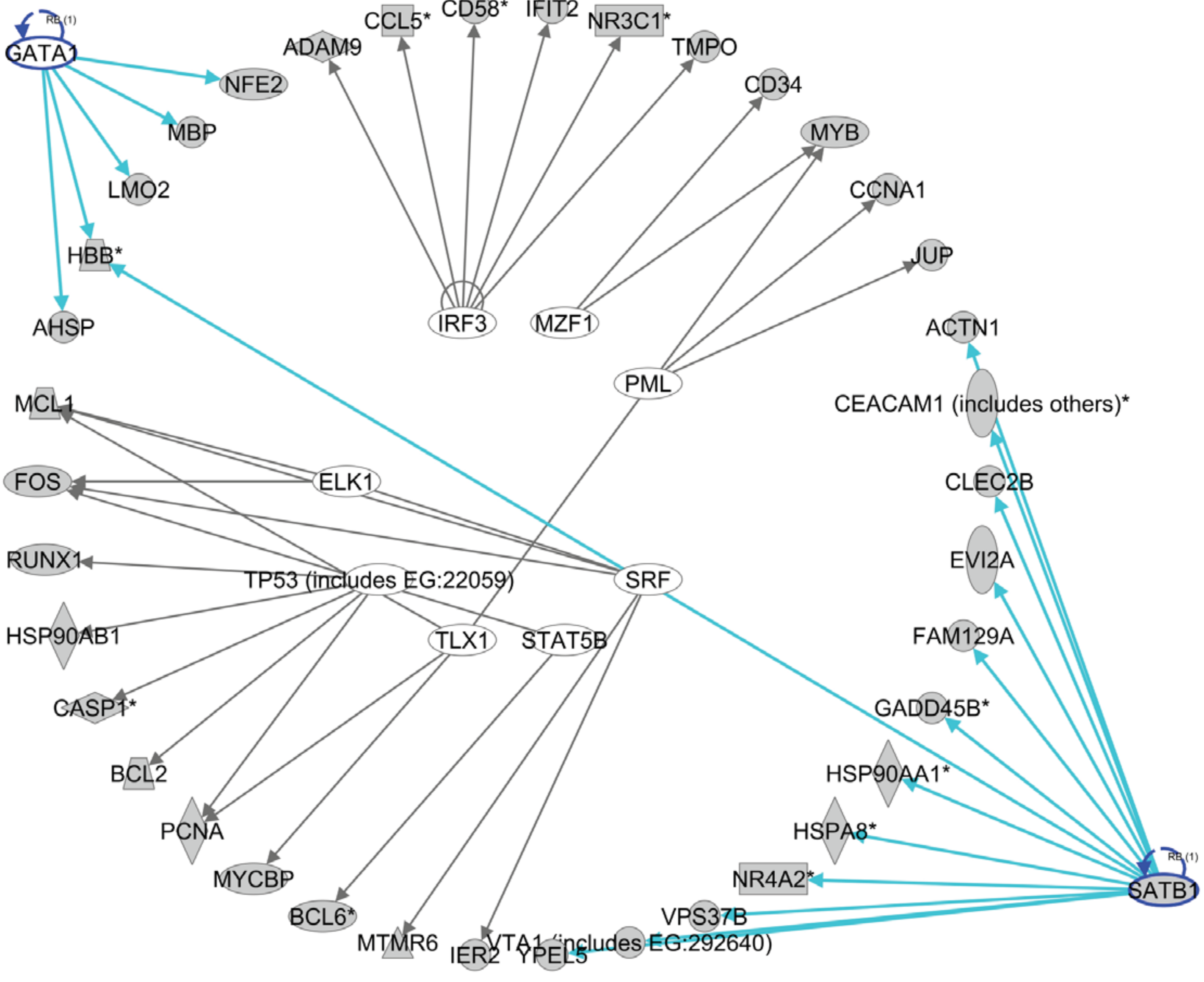

Transcription factor analysis visualization plot of patient 15

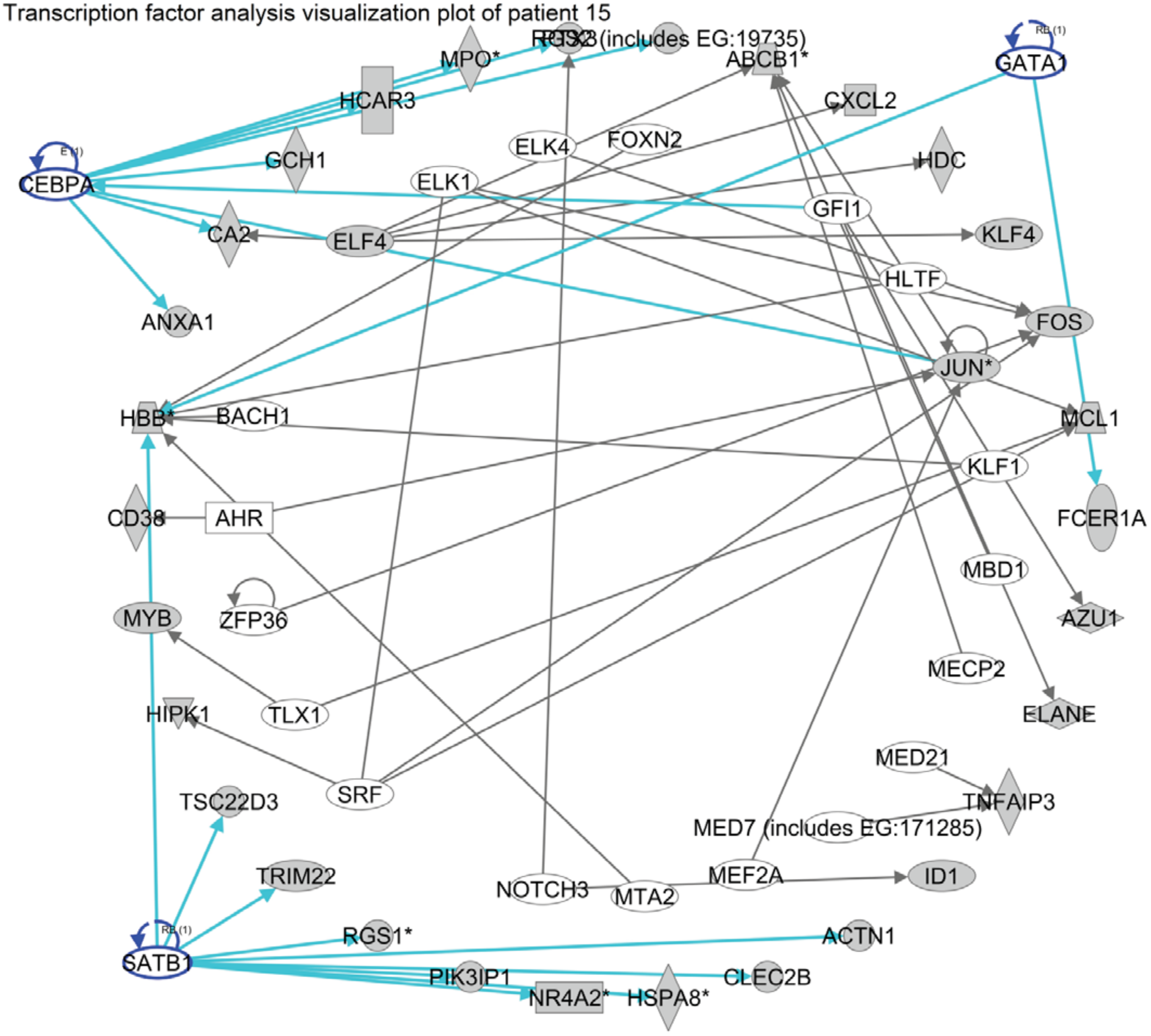

Transcription factor analysis visualization plot of patient 16

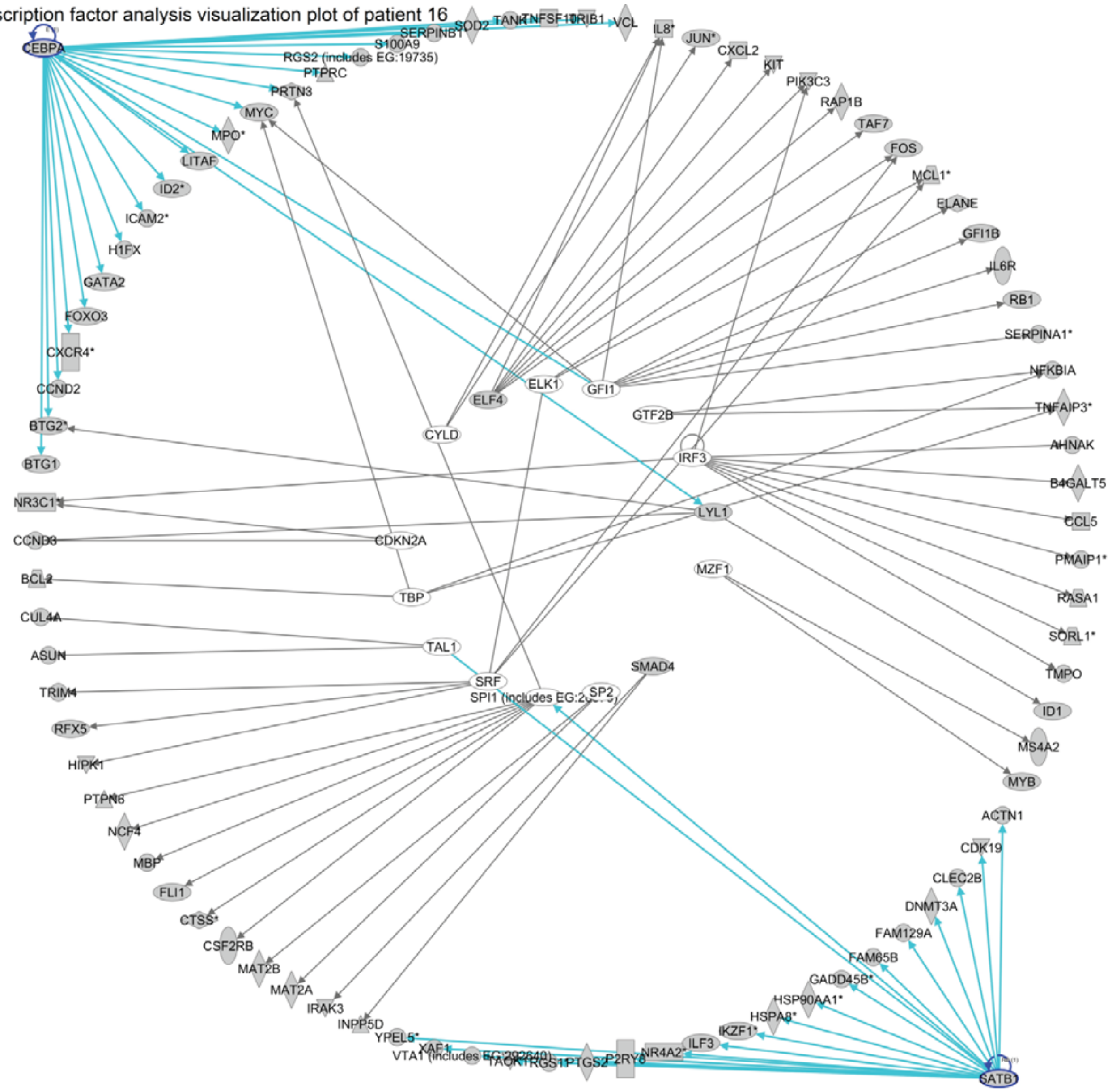

Transcription factor analysis visualization plot of patient 17

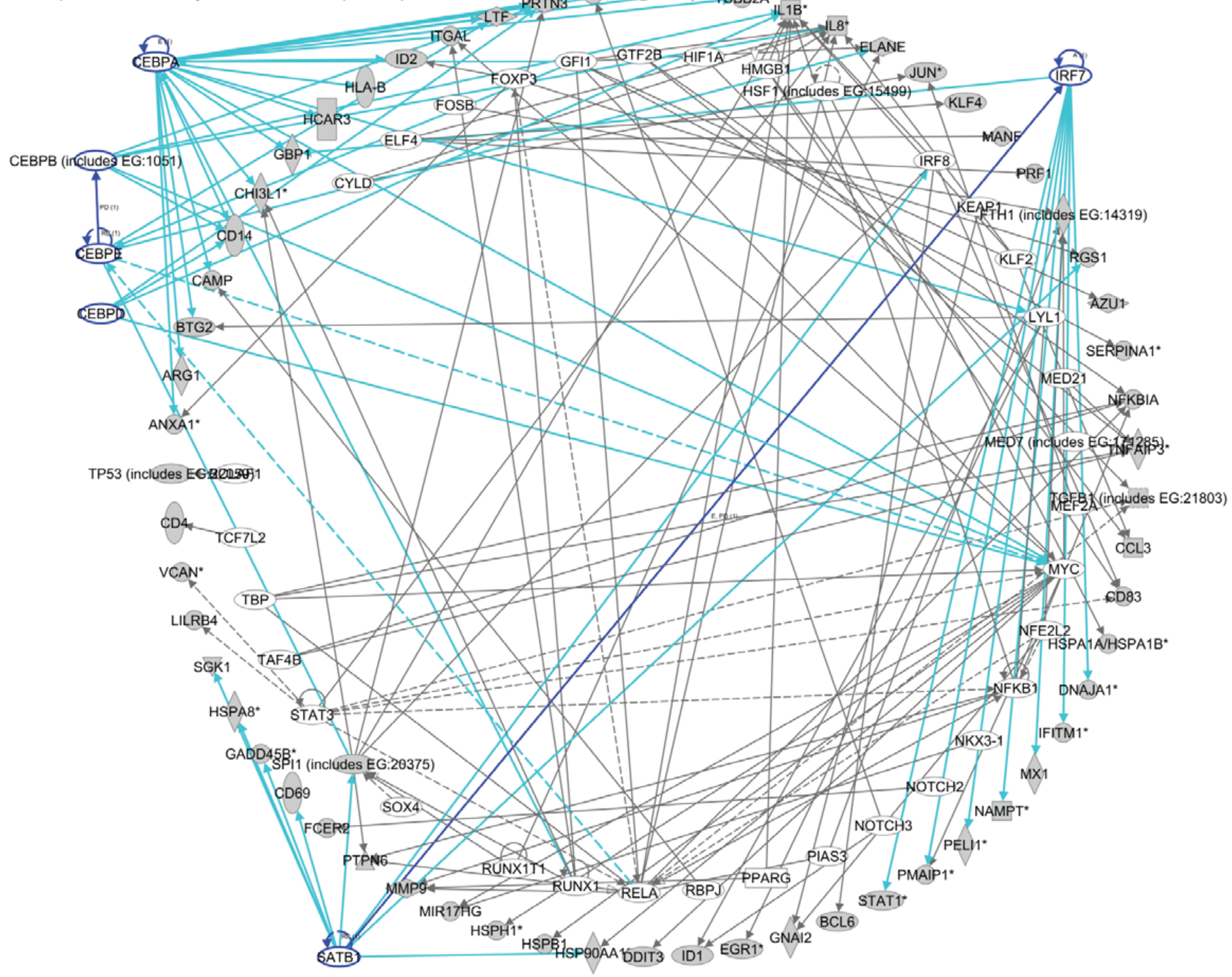

Transcription factor analysis visualization plot of patient 18

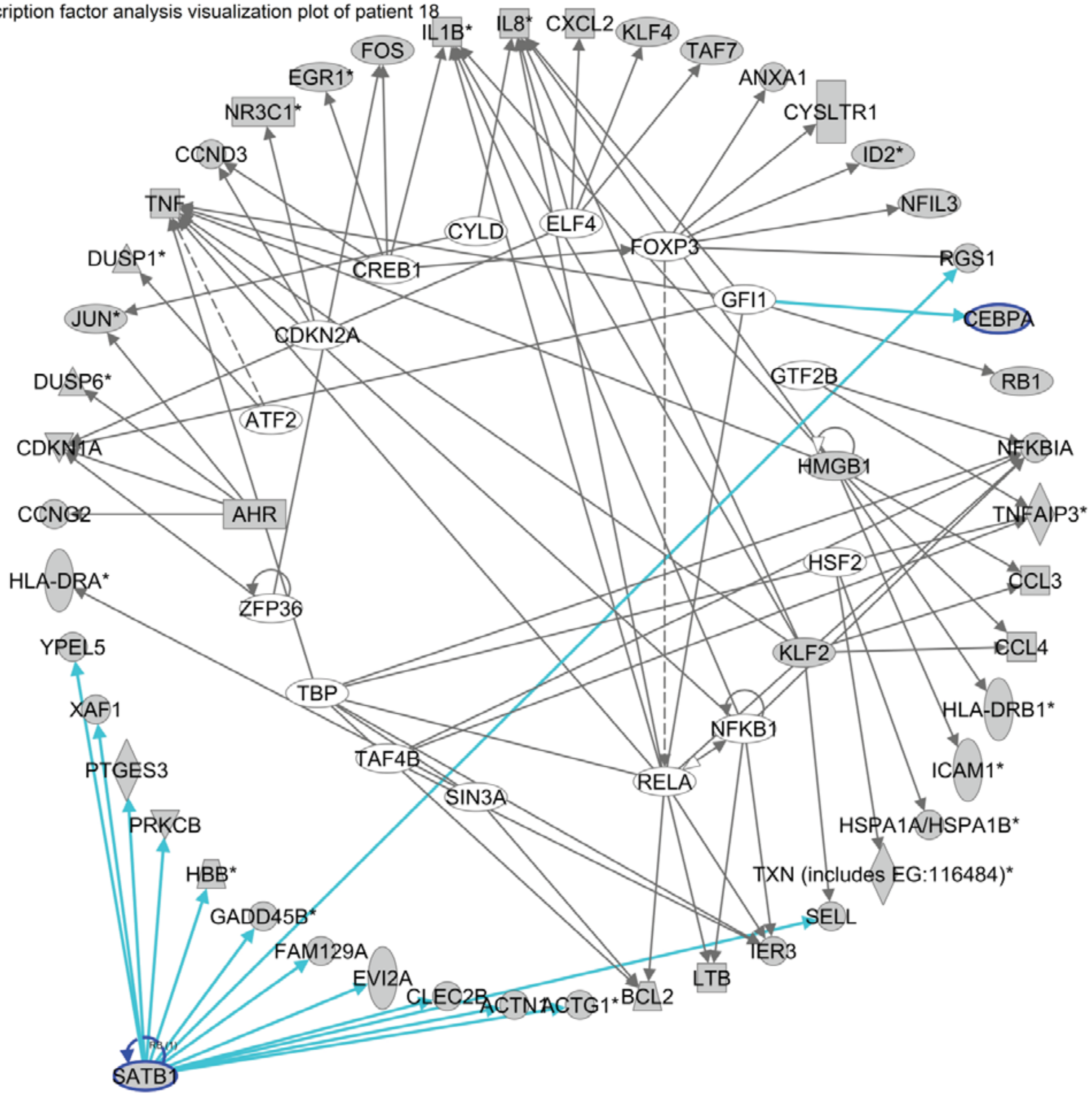

Transcription factor analysis visualization plot of patient 19

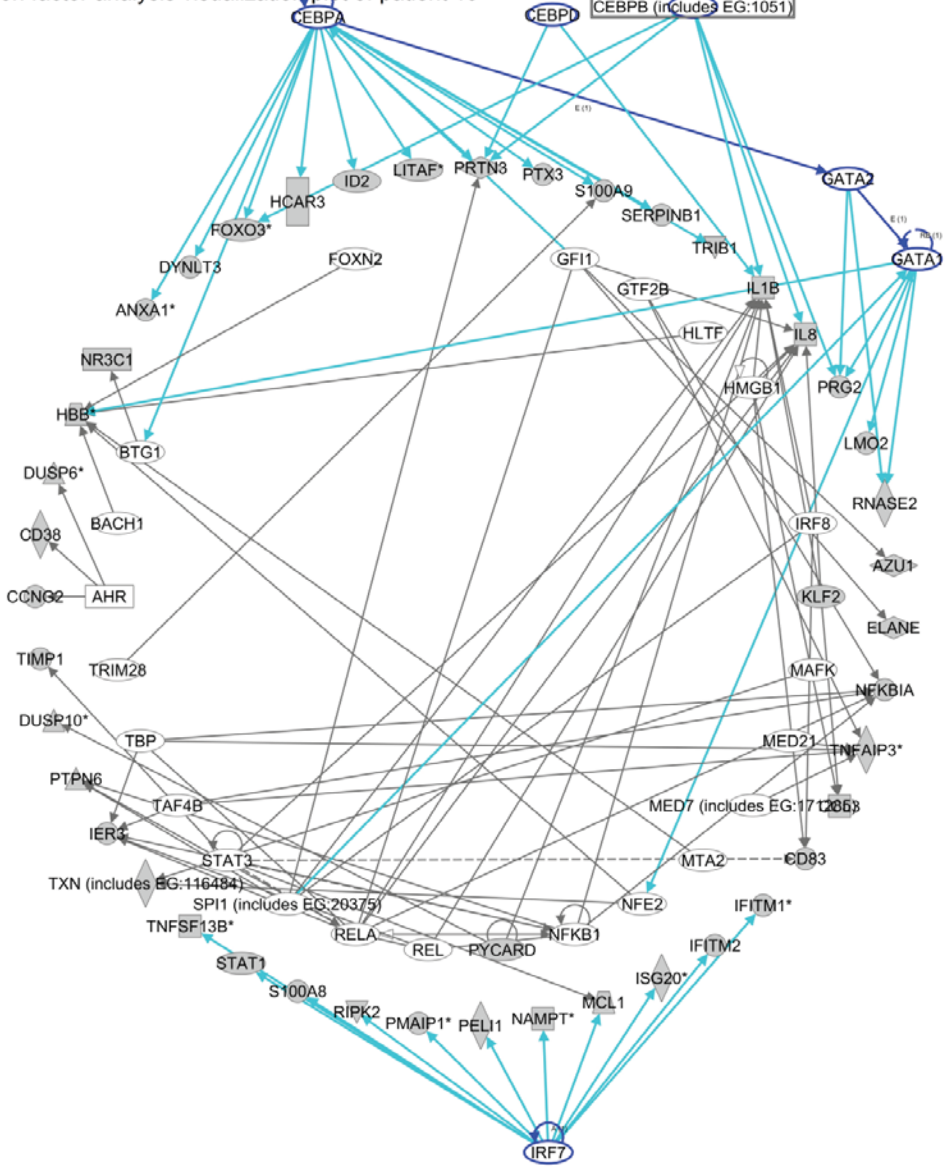

Transcription factor analysis visualization plot of patient 20

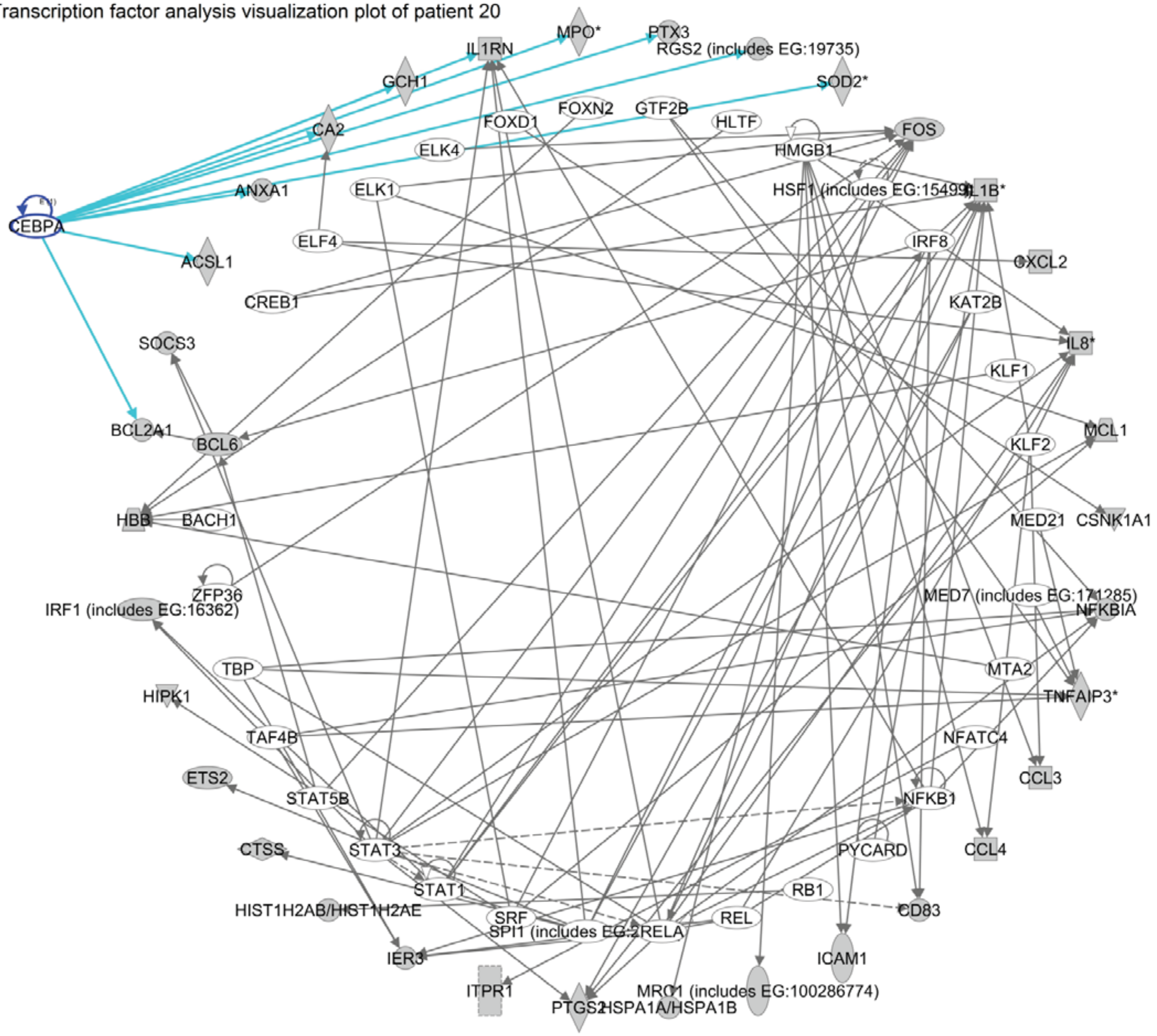

Transcription factor analysis visualization plot of patient 21

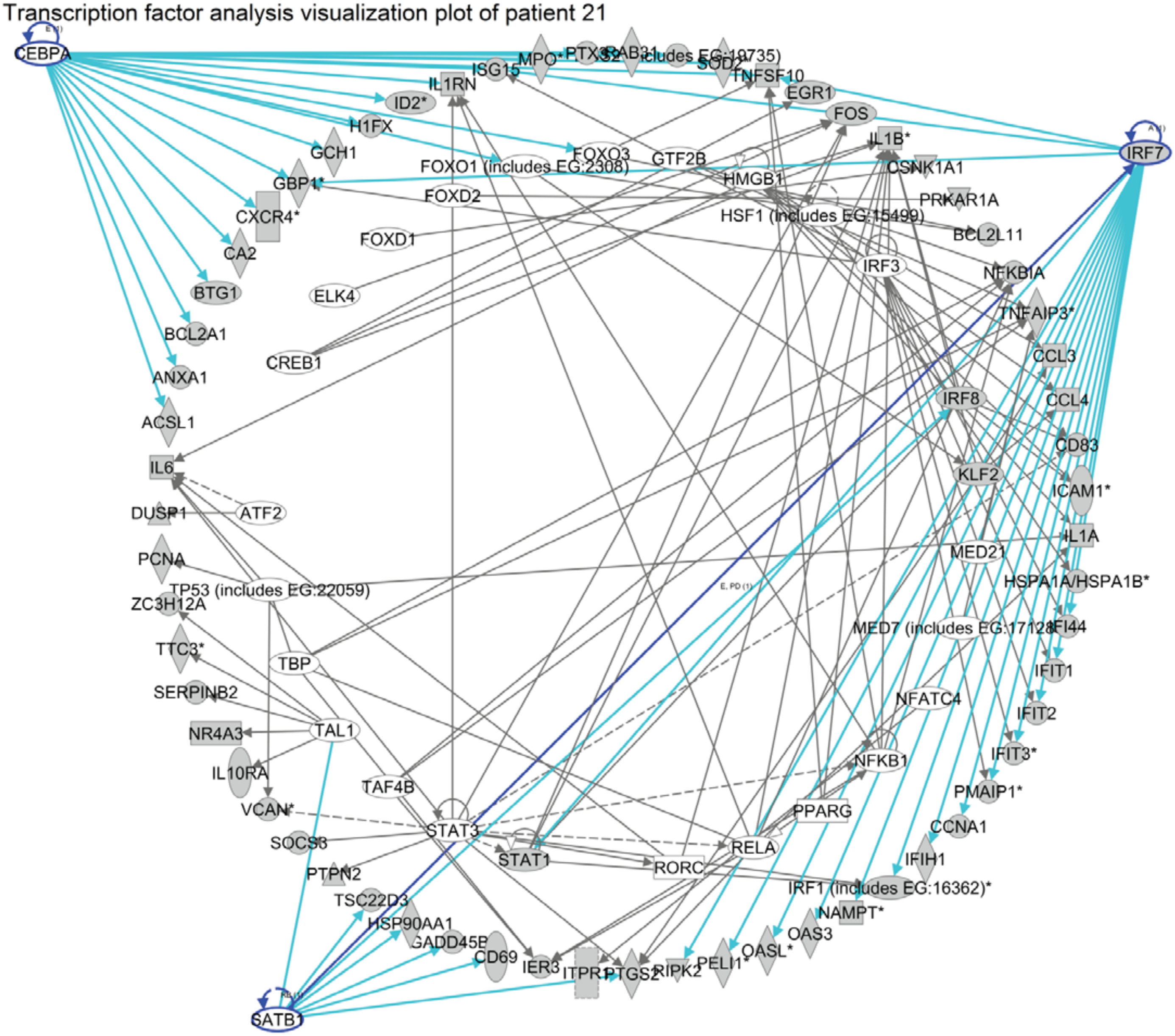

Transcription factor analysis visualization plot of patient 22

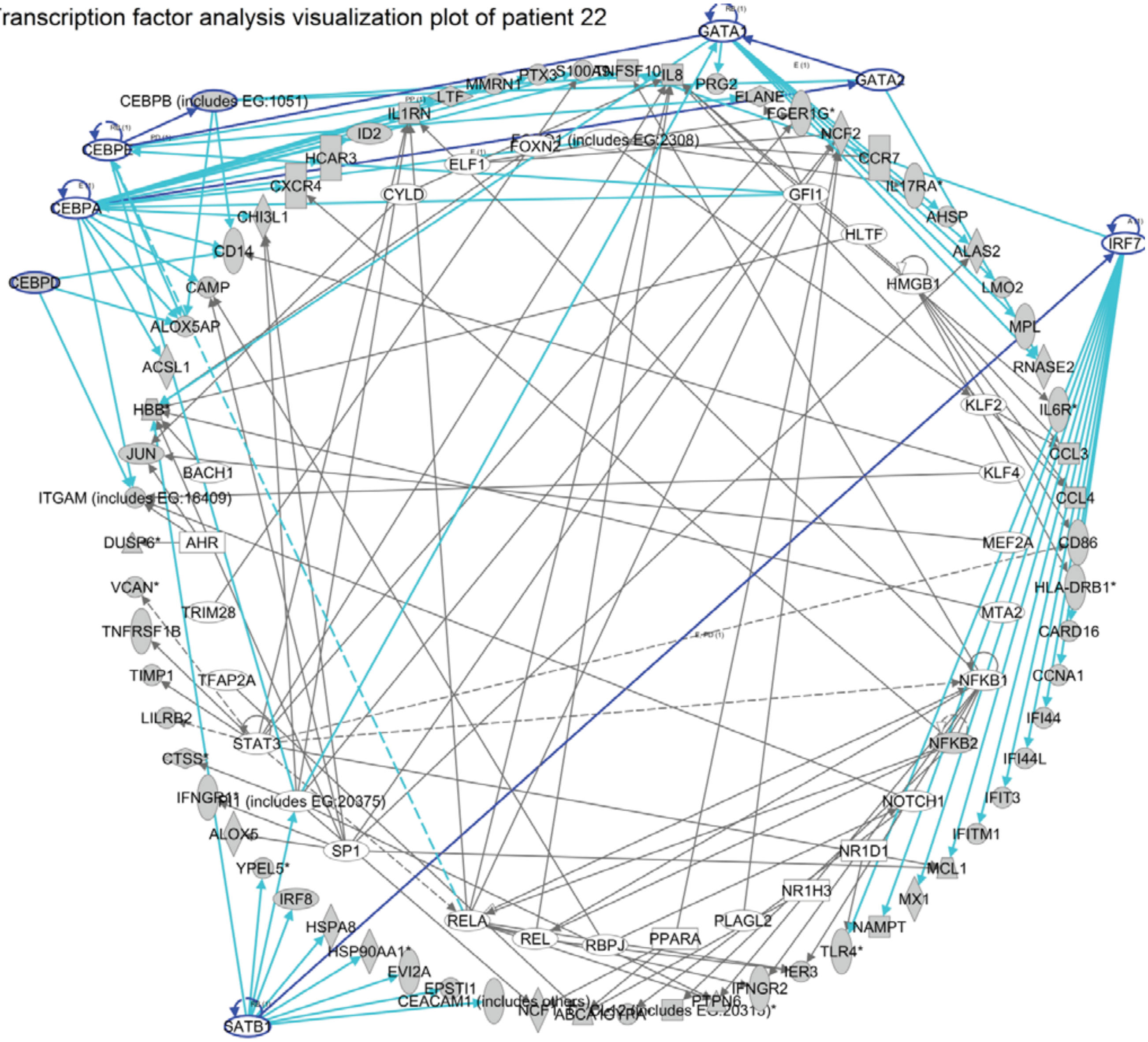

Transcription factor analysis visualization plot of patient 23

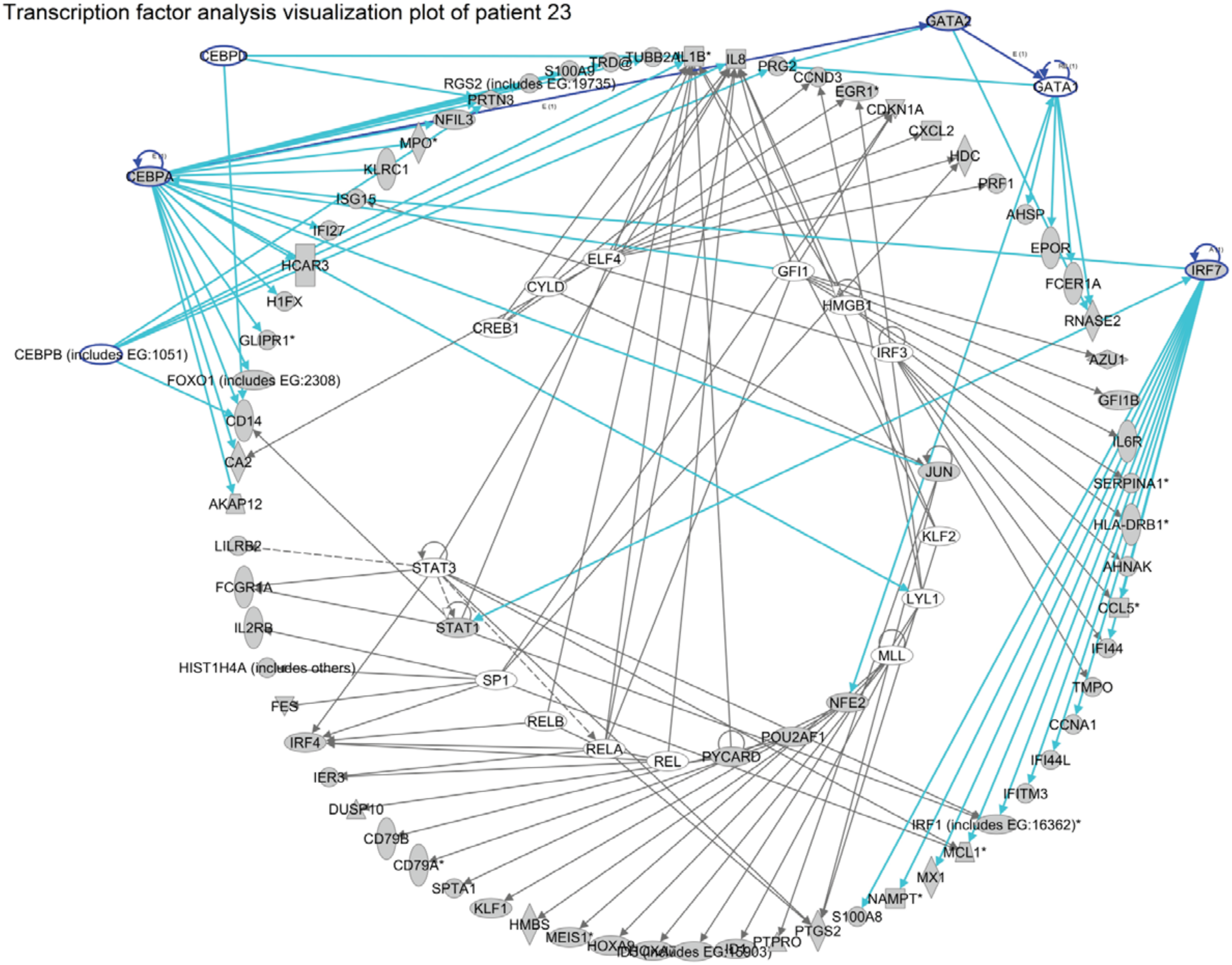

Supplement: S2 Fig — Transcription network plot showing transcription factors (outer ring/ inner ring) that are predicted responsible for differential expression of shown target molecules (middle ring) between diagnosis and relapse. A few transcription factors (CEBPA, GFI1, SATB1 and TBP) are responsible for the major changes in the differentially expressed target molecules. (PDF) [file pone.0121730.s002.pdf]
