# Supplementary material for: Gene Expression Profiles Associated with Pediatric Relapsed AML
Source: PLoS One. 2015 Apr 7;10(4):e0121730. doi: 10.1371/journal.pone.0121730 (PMC4388534; doi:10.1371/journal.pone.0121730)
Supplement: S1 Supplementary Information — (DOC) [file pone.0121730.s003.doc]

# Supporting information

## Bioinformatical analysis of paired diagnosis and relapse pediatric AML gene expression micro array data

### Data pre-processing

Micro array data was obtained as previously described (1). The raw microarray data are available as .CEL files from GEO database (GSE17855/GSE52891 http://www.ncbi.nlm.nih.gov/geo/). Quality of the data was assessed using image analysis and the Bioconductor packages ([http://www.bioconductor.org](http://www.bioconductor.org/)) ‘affy’ and ‘AffyPLM’.

Probe level normalization was performed using the variance stabilizing method in the R statistical environment (http://www.R-project.org) using the Bioconductor packages “affy” and “vsn”. Probe set data was summarized using the median polish method on the normalized data

The data were then imported into BRB-ArrayTools 4.2.1 (http://linus.nci.nih.gov/BRB-ArrayTools.html) developed by Simon and colleagues (2). Individual spots were excluded from the analysis if the intensity was ≤30.

### Analyses of differential gene expression between diagnosis and relapse samples

We scatter plotted the data of diagnosis and corresponding relapse samples of individual patients and checked if the data was linearly related. For initial class comparison analyses (either paired or unpaired), genes with low variation in expression levels were excluded if ≤ 3-fold deviation from the median was seen in ≤ 2% of samples. Accordingly, 3083 probe sets were used for class comparison analyses. First we performed paired SAM analysis to determine expression differences between samples that were obtained at diagnosis and the corresponding sample of the same patient that was obtain at relapse (2). Using false discovery rates between 0.05 and 0.3, confidence intervals between 80-95% and 1000 permutations per test, we were not able to assess significantly differentially expressed genes. When performing a SAM class comparison between the group of diagnosis and the group of relapse samples, low stringent settings (FDR=0.3, CI=0.80, 1000 permutations), yielded a list of only 22 probe sets (Supplementary Figure S1).

We restored pre-filter settings to the intensity filter and proceeded by looking for a gene expression signature (rather than individual genes) that was discriminative between diagnosis (class 1) and relapse samples (class 2) by applying prediction analysis. We applied compound covariate prediction for two classes, diagonal linear discriminant analysis, K-nearest neighbours prediction (1,3), nearest centroid and support vector machine prediction. Gene selection was optimized over a grid of alpha levels (0.01, 0.005, 0.001 and 0.0005). A leave-one-out cross validation was applied to validate the classifier and compute the misclassification rate. The predictor with the lowest cross-validation misclassification rate was selected. The best compound covariate classifier consisted of genes significantly different between the classes at the 5e-04 significance level. The best diagonal linear discriminant analysis classifier consisted of genes significantly different between the classes at the 5e-04 significance level. The best 1-nearest neighbour classifier consisted of genes significantly different between the classes at the 0.005 significance level. The best 3-nearest neighbours classifier consisted of genes significantly different between the classes at the 0.005 significance level. The best nearest centroid classifier consisted of genes significantly different between the classes at the 5e-04 significance level. The best support vector machines classifier consisted of genes significantly different between the classes at the 0.005 significance level. The probe-sets that built the classifier are shown in Supplementary Table S3 and Figure 2.

### Pathway analysis

Recognizing the importance of intra-patient differences between diagnosis and relapse, we focussed on the paired samples of individuals. For pathway analysis, genes were selected that were differentially expressed by at least 2 fold change between diagnosis and relapse samples of individual patients, without any other pre-filtering of probe sets.

**Ingenuity pathway analysis**

To assess pathways that may play a role in relapse development, we subjected the 23 lists of ≥2 FC differentially expressed probe sets of individual patients to Ingenuity Pathway analysis.

Ingenuity’s Upstream Regulator Analysis in IPA is a tool that predicts upstream regulators that may explain a user's list of differentially expressed genes by testing for target enrichment from gene expression data based on the literature and compiled in the Ingenuity® Knowledge Base (genes only). We preformed the analysis based on direct or indirect relationships between regulators and targets. Only human hematopoietic tissue data or data from human or mouse hematopoietic or blood cell disorder cell lines from the Ingenuity® Knowledge Base were included. A Fisher’s Exact Test p-value is calculated to assess the significance of enrichment of the gene expression data for the genes downstream of an upstream regulator. The obtained 23 lists of upstream regulators are shown in Supplementary Table S4 and visualized in the Network visualization plots of Supplementary Figure S2.

### References

1. Balgobind BV, Zwaan CM, Reinhardt D, Arentsen-Peters TJ, Hollink IH, de Haas V, Kaspers GJ, de Bont ES, Baruchel A, Stary J, Meyer C, Marschalek R, Creutzig U, den Boer ML, Pieters R, van den Heuvel-Eibrink MM. High BRE expression in pediatric MLL-rearranged AML is associated with favorable outcome. Leukemia, 2010;24(12):2048-55.
2. Simon R, Lam A, Li MC, Ngan M, Menenzes S, Zhao Y. Analysis of Gene Expression Data Using BRB-Array Tools. Cancer informatics 2007; 3:11-7.
3. Tusher VG, Tibshirani R, Chu G. Significance analysis of microarrays applied to the ionizing radiation response. Proc Natl Acad Sci U S A. 2001 Apr 24;98(9):5116-21
