# Supplementary material for: Gene Expression Profiles Associated with Pediatric Relapsed AML
Source: PLoS One. 2015 Apr 7;10(4):e0121730. doi: 10.1371/journal.pone.0121730 (PMC4388534; doi:10.1371/journal.pone.0121730)
Supplement: S1 Table — (PDF) [file pone.0121730.s004.pdf]

Supplementary Table 1. Mutation analysis of paired primary and relapse AML samples

Pair#	Stage	Response		*FLT3/ITD*	*RAS*	*NPM1*	*KIT*	*PTPN11*	*CEBPA*	*WT1*	Shift	Cytogenetic change
1	Diagnosis	CR	46,XY,del(10)(p12),der(11)t(10;11)(p12;q2?3) [20]									
	Relapse		ND									
2	Diagnosis	CR	46,XY[20]	1							yes	no
	Relapse		46,XY[20]	1						**1**		
3	Diagnosis	CR	45,X0							1		
	Relapse		ND							1		
4	Diagnosis	CR	46,XY,t(6;9)(p23;q34)[22]								yes	no
	Relapse		46,XY,t(6;9)(p23;q34)[22]	**1**						**1**		
5	Diagnosis	CR	46,XY [20]									yes
	Relapse		46~47,X,der(Y)t(Y;1)(q12;q12),der(8;12)(q10;q10),+1-2 mar [8]									
6	Diagnosis	CR	ND			1						
	Relapse		ND			1						
7	Diagnosis	NA	t(6;9)(p23;q34)	1						1		
	Relapse		ND	1						1		
8	Diagnosis	CR	46,XY,t(16;21)(p11;q22) [14] 46,XY,t(16;21)(p11;q22),i(22)(q10) [5]									yes
	Relapse		46,XY,del(3)(q21q27),dup(4)(q2),del(7)(q22q36), t(16;21)(p11;q22),der(18)t(1;18)(q23;q23)/46,idem,add(8)(p21)/46,XX[donor]									
9	Diagnosis	NA	t(9;11)									no
	Relapse		t(9;11)									
10	Diagnosis	PR	deviate from normal		**1**						yes	
	Relapse		ND	**1**								
11	Diagnosis	CR	46,XY,-Y,t(8;21)(q22;q22),+der(21)t(8;21)(q22;q22)	**1**							yes	
	Relapse		ND									
12	Diagnosis	CR	46,XY,t(8;21)(q22;q22) [24]				1					
	Relapse		ND				1					
13	Diagnosis	CR	45,X,-Y,t(8;21)(q22;q22)[7]/46,idem,+4[13]				1					yes
	Relapse		45,X,-Y,t(8;21)(q22;q22)[3]/46,idem,+4[3]/46,idem,?dup(1)(p1?3p3?3),+4[4]				1					
14	Diagnosis	PR	43~45,XY,add(6)(p2?2),del(8;?),add(8)(q2?3),?del(9)(q?),add(11)(p1?1),-16[cp20]							1		
	Relapse		ND							1		
15	Diagnosis	CR	46,XY,t(8;21)(q22;q22)[20]/46,XY[1]									yes
	Relapse		46,XY,t(8;21)(q22;q22),add(11)(?q23)[16]/46,XY,t(8;21)(q22;q22)[3]/46,XY,t(8;21)(q22;q22)[3]/46,idem,add(11)(?q23)[16]									
16	Diagnosis	CR	46,XX,?t(11;20)(p15;q1?2) [20] .ish t(11;20)						1	1	yes	yes
	Relapse		46,XX,t(11;20)(p15;q1?2)[8]/46,XX,del(9)(q22),t(11;20)(p15;q1?2)[6]/46,XX,add(1)(q?32),del(9)(q22),t(11;20)(p15;q1?2)[4]/46,XX,del(9)(q?22),add(9)(q?34),-11,der(20)t(11;20)(p15;q1?2),+mar[2]		**1**				1	1		
17	Diagnosis	NA	46,XY,add(11)(q23)[19]/46,XY[2]/FISH: fusie van FISH signalen van 5'MLL en AF10[0]								yes	
	Relapse		ND		**1**							
18	Diagnosis	CR	46,XX		1	1						
	Relapse		ND		1	1						
19	Diagnosis	NA	46,XY,t(8;21)(q22;q22)[3]/45,X,-Y,t(8;21)(q22;q22)[7]									yes
	Relapse		46,XY,t(8;21)(q22;q22),del(9)(q22)[6]/46,XY[4]									
20	Diagnosis	CR	46,XY,del(9)(q2?1q3?2)[7]/46,XY[13]									
	Relapse		ND									
21	Diagnosis	CR	46,XY[20]	1							yes	
	Relapse		ND	1						**1**		
22	Diagnosis	CR	46,XX,t(9;11)(p22;q23)[5]									yes
	Relapse		47,XX,t(9;11)(p22;q23),+21[12]/46,XX[8]									
23	Diagnosis	NA	45, XY, -7					**1**			yes	
	Relapse		ND									

1 = mutated for selected gene, bold font = mutational shift between diagnosis and relapse, CR=complete response, PR=partial response, NA=response data not aqcuired
