# Supplementary material for: Gene Expression Profiles Associated with Pediatric Relapsed AML
Source: PLoS One. 2015 Apr 7;10(4):e0121730. doi: 10.1371/journal.pone.0121730 (PMC4388534; doi:10.1371/journal.pone.0121730)
Supplement: S2 Table — (PDF) [file pone.0121730.s005.pdf]

Supplementary Table 2 List of probesets that are co-expressed with HIST1H1C expression

ProbeSet	Symbol	Correlation value
209398_at	HIST1H1C	1.00
218280_x_at	HIST2H2AA3	0.84
222067_x_at	HIST1H2BD	0.83
214522_x_at	HIST1H2AD	0.82
208583_x_at	HIST1H2AJ	0.82
214542_x_at	HIST1H2AI	0.81
214290_s_at	HIST2H2AA4	0.81
208527_x_at	HIST1H2BE	0.80
208490_x_at	HIST1H2BF	0.80
214455_at	HIST1H2BC	0.79
208523_x_at	HIST1H2BI	0.78
208546_x_at	HIST1H2BH	0.75
202708_s_at	HIST2H2BE	0.72
208579_x_at	H2BFS	0.71
214472_at	HIST1H3D	0.69
236193_at	HIST1H2BC	0.67
214481_at	HIST1H2AM	0.63
239669_at	HIST1H2AD	0.61
215071_s_at	HIST1H2AC	0.61
208553_at	HIST1H1E	0.61
214554_at	HIST1H2AL	0.60
214469_at	HIST1H2AE	0.59
214540_at	HIST1H2BO	0.58
208575_at	HIST1H3A	0.58
206110_at	HIST1H3H	0.58
206951_at	HIST1H4E	0.57
211999_at	H3F3B	0.56
209806_at	HIST1H2BK	0.55
1556814_a_at	---	0.55
209069_s_at	H3F3B	0.54
211997_x_at	H3F3B	0.53
209911_x_at	HIST1H2BD	0.53
215779_s_at	HIST1H2BG	0.52
230795_at	HIST2H4	0.51
204805_s_at	H1FX	0.51
