# Supplementary material for: Gene Expression Profiles Associated with Pediatric Relapsed AML
Source: PLoS One. 2015 Apr 7;10(4):e0121730. doi: 10.1371/journal.pone.0121730 (PMC4388534; doi:10.1371/journal.pone.0121730)
Supplement: S3 Table — (PDF) [file pone.0121730.s006.pdf]

Supplementary Table 3 List of 306 probe-sets of the classifier that discrminates between initial and relapse AML samples, ranked according to *P-value*

Geometric mean of intensities relapse/diagnosis	Parametric p-value	t-value	ProbeSet	Symbol	Name
0.93	0.0000015000	-6.521	202175_at	CHPF	chondroitin polymerizing factor
1.17	0.0000092000	5.727	230462_at	NUMB	numb homolog (Drosophila)
0.92	0.0000121000	-5.615	206815_at	SPAG8	sperm associated antigen 8
0.91	0.0000224000	-5.355	206408_at	LRRTM2	leucine rich repeat transmembrane neuronal 2
0.92	0.0000739000	-4.860	230228_at	SSC5D	scavenger receptor cysteine rich domain containing (5 domains)
0.91	0.0000765000	-4.846	1569904_x_at	NA	NA
1.06	0.0000794000	4.831	219943_s_at	USP46	ubiquitin specific peptidase 46
1.24	0.0000930000	4.766	225941_at	EIF4E3	eukaryotic translation initiation factor 4E family member 3
1.38	0.0001154000	4.677	224558_s_at	MALAT1	metastasis associated lung adenocarcinoma transcript 1 (non-protein coding)
0.94	0.0001276000	-4.636	232211_at	PPP1R3F	protein phosphatase 1, regulatory (inhibitor) subunit 3F
1.67	0.0001277000	4.636	224917_at	NA	NA
1.3	0.0001355000	4.612	241751_at	OFD1	oral-facial-digital syndrome 1
1.11	0.0001480000	4.575	221813_at	FBXO42	F-box protein 42
0.94	0.0001517000	-4.565	214432_at	ATP1A3	ATPase, Na+/K+ transporting, alpha 3 polypeptide
0.95	0.0001597000	-4.544	223975_at	SPRYD5	SPRY domain containing 5
1.06	0.0001867000	4.480	1570600_at	NA	NA
1.34	0.0001877000	4.478	204872_at	TLE4	transducin-like enhancer of split 4 (E(sp1) homolog, Drosophila)
0.94	0.0001977000	-4.457	233787_at	C6orf163	chromosome 6 open reading frame 163
1.08	0.0002091000	4.434	205094_at	PEX12	peroxisomal biogenesis factor 12
0.94	0.0002258000	-4.402	215370_at	NA	NA
0.93	0.0002332000	-4.389	240720_at	NA	NA
0.94	0.0002475000	-4.365	226051_at	SELM	selenoprotein M
1.24	0.0002629000	4.340	231310_at	NA	NA
1.23	0.0002823000	4.311	217730_at	TMBIM1	transmembrane BAX inhibitor motif containing 1
0.9	0.0002949000	-4.293	228376_at	GGTA1	glycoprotein, alpha-galactosyltransferase 1 pseudogene
0.93	0.0003195000	-4.260	227023_at	NA	NA
0.92	0.0003221000	-4.257	239799_at	C9orf130	chromosome 9 open reading frame 130
1.26	0.0003358000	4.240	225940_at	EIF4E3	eukaryotic translation initiation factor 4E family member 3
1.07	0.0003630000	4.208	1569201_a_at	NA	NA
0.9	0.0003670000	-4.204	222720_x_at	C1orf27	chromosome 1 open reading frame 27
0.94	0.0003915000	-4.177	204400_at	EFS	embryonal Fyn-associated substrate
1.18	0.0003984000	4.170	222163_s_at	SPATA5L1	spermatogenesis associated 5-like 1
0.95	0.0004119000	-4.156	234057_at	NA	NA
1.13	0.0004546000	4.116	212804_s_at	GAPVD1	GTPase activating protein and VPS9 domains 1
1.16	0.0005113000	4.068	213926_s_at	AGFG1	ArfGAP with FG repeats 1
1.14	0.0005208000	4.060	226175_at	TTC9C	tetratricopeptide repeat domain 9C
0.9	0.0005213000	-4.060	211909_x_at	PTGER3	prostaglandin E receptor 3 (subtype EP3)
0.93	0.0005317000	-4.052	210089_s_at	LAMA4	laminin, alpha 4
0.94	0.0005495000	-4.038	205272_s_at	NA	NA
0.94	0.0005739000	-4.020	216358_at	NA	NA
1.11	0.0005834000	4.014	202055_at	KPNA1	karyopherin alpha 1 (importin alpha 5)
0.9	0.0005971000	-4.004	238057_at	USP45	ubiquitin specific peptidase 45
0.94	0.0006086000	-3.996	237087_at	NA	NA
0.95	0.0006206000	-3.988	217156_at	NA	NA
1.19	0.0006671000	3.959	225378_at	VPS37A	vacuolar protein sorting 37 homolog A (S. cerevisiae)
0.92	0.0006853000	-3.948	204292_x_at	STK11	serine/threonine kinase 11
1.14	0.0006925000	3.943	223089_at	VEZT	vezatin, adherens junctions transmembrane protein
0.94	0.0006984000	-3.940	207896_s_at	DLEC1	deleted in lung and esophageal cancer 1
1.06	0.0007058000	3.935	1569597_at	NA	NA
0.94	0.0007063000	-3.935	239576_at	MTUS1	microtubule associated tumor suppressor 1
0.95	0.0007225000	-3.926	244572_at	KY	kyphoscoliosis peptidase
0.92	0.0007503000	-3.910	229561_at	LRRC16B	leucine rich repeat containing 16B
1.16	0.0007711000	3.899	203615_x_at	SULT1A1	sulfotransferase family, cytosolic, 1A, phenol-preferring, member 1
0.89	0.0007723000	-3.898	204718_at	EPHB6	EPH receptor B6
0.95	0.0008226000	-3.873	206913_at	BAAT	bile acid CoA: amino acid N-acyltransferase (glycine N-choloyltransferase)
1.14	0.0008631000	3.853	222839_s_at	PAPOLG	poly(A) polymerase gamma

Supplementary Table 3 List of 306 probe-sets of the classifier that discrminates between initial and relapse AML samples, ranked according to *P-value*					
**Geometric mean of intensities relapse/diagnosis**	**Parametric p-value**	**t-value**	**ProbeSet**	**Symbol**	**Name**
1.12	0.0008901000	3.840	219340_s_at	CLN8	ceroid-lipofuscinosis, neuronal 8 (epilepsy, progressive with mental retardation)
1.15	0.0009008000	3.835	225939_at	EIF4E3	eukaryotic translation initiation factor 4E family member 3
0.95	0.0009129000	-3.830	208432_s_at	CACNA1E	calcium channel, voltage-dependent, R type, alpha 1E subunit
1.09	0.0009449000	3.815	219124_at	C8orf41	chromosome 8 open reading frame 41
0.93	0.0009468000	-3.815	213650_at	NA	NA
1.05	0.0010180000	3.785	1557311_at	LOC100131354	hypothetical protein LOC100131354
0.95	0.0010491000	-3.772	228965_s_at	PANK2	pantothenate kinase 2
1.04	0.0010635000	3.767	1558645_at	MIB1	mindbomb homolog 1 (Drosophila)
0.94	0.0010677000	-3.765	229009_at	SIX5	SIX homeobox 5
0.94	0.0011042000	-3.751	204724_s_at	COL9A3	collagen, type IX, alpha 3
0.92	0.0011099000	-3.749	225428_s_at	DDX54	DEAD (Asp-Glu-Ala-Asp) box polypeptide 54
0.94	0.0011516000	-3.734	240672_at	NA	NA
1.05	0.0011611000	3.730	238374_at	H1FNT	H1 histone family, member N, testis-specific
0.94	0.0011808000	-3.724	241637_at	NA	NA
1.13	0.0011808000	3.724	230590_at	NA	NA
1.06	0.0011852000	3.722	224028_at	CPA6	carboxypeptidase A6
1.75	0.0011881000	3.721	227769_at	GPR27	G protein-coupled receptor 27
0.93	0.0012068000	-3.715	228405_at	RHPN1	rhophilin, Rho GTPase binding protein 1
0.94	0.0012219000	-3.709	1555748_x_at	CD79B	CD79b molecule, immunoglobulin-associated beta
0.91	0.0012706000	-3.693	217555_at	SMC1A	structural maintenance of chromosomes 1A
1.04	0.0012758000	3.692	1562918_at	NA	NA
0.83	0.0012796000	-3.690	221861_at	ARRB1	arrestin, beta 1
1.07	0.0012922000	3.686	223313_s_at	NA	NA
0.95	0.0012958000	-3.685	206623_at	PDE6A	phosphodiesterase 6A, cGMP-specific, rod, alpha
0.95	0.0013207000	-3.677	216827_at	NA	NA
1.15	0.0013556000	3.666	244075_at	NA	NA
0.95	0.0013737000	-3.661	216636_at	NA	NA
0.96	0.0014019000	-3.653	230818_at	NA	NA
0.93	0.0014343000	-3.643	1567575_at	NA	NA
1.1	0.0014665000	3.634	228037_at	NA	NA
1.07	0.0014732000	3.632	1562511_at	LYST	lysosomal trafficking regulator
0.91	0.0014767000	-3.631	205375_at	MDFI	MyoD family inhibitor
0.94	0.0014928000	-3.627	211577_s_at	IGF1	insulin-like growth factor 1 (somatomedin C)
1.04	0.0015095000	3.622	228085_at	LOC100507419	hypothetical protein LOC100507419
1.06	0.0015171000	3.620	238818_at	KIAA1429	KIAA1429
1.18	0.0015522000	3.610	213291_s_at	UBE3A	ubiquitin protein ligase E3A
1.37	0.0015638000	3.607	214472_at	NA	NA
1.07	0.0015645000	3.607	204863_s_at	IL6ST	interleukin 6 signal transducer (gp130, oncostatin M receptor)
0.94	0.0015744000	-3.604	229365_at	PPP1R3F	protein phosphatase 1, regulatory (inhibitor) subunit 3F
0.96	0.0015810000	-3.603	221977_at	TBX2	T-box 2
0.93	0.0015925000	-3.600	217277_at	NA	NA
1.06	0.0016159000	3.594	1554557_at	ATP11B	ATPase, class VI, type 11B
0.95	0.0016215000	-3.592	216402_at	SEC14L4	SEC14-like 4 (S. cerevisiae)
0.93	0.0016246000	-3.591	215384_s_at	MAP1A	microtubule-associated protein 1A
1.16	0.0016539000	3.584	201997_s_at	SPEN	spen homolog, transcriptional regulator (Drosophila)
1.07	0.0016572000	3.583	224276_at	ZNF33A	zinc finger protein 33A
0.94	0.0016629000	-3.582	205168_at	DDR2	discoidin domain receptor tyrosine kinase 2
0.96	0.0016655000	-3.581	234275_at	NA	NA
1.18	0.0016669000	3.581	201628_s_at	RRAGA	Ras-related GTP binding A
0.94	0.0016933000	-3.574	221198_at	SCT	secretin
0.9	0.0017242000	-3.567	1568754_at	NA	NA
0.92	0.0017295000	-3.565	1555475_x_at	TTLL3	tubulin tyrosine ligase-like family, member 3
0.95	0.0017313000	-3.565	1553467_at	FLJ32742	hypothetical locus FLJ32742
1.08	0.0017889000	3.551	217948_at	FAM127B	family with sequence similarity 127, member B
1.22	0.0018248000	3.543	223081_at	PHF23	PHD finger protein 23

Supplementary Table 3 List of 306 probe-sets of the classifier that discrminates between initial and relapse AML samples, ranked according to *P-value*					
**Geometric mean of intensities relapse/diagnosis**	**Parametric p-value**	**t-value**	**ProbeSet**	**Symbol**	**Name**
0.83	0.0018480000	-3.538	49111_at	ARRB1	arrestin, beta 1
0.96	0.0018784000	-3.531	213307_at	SHANK2	SH3 and multiple ankyrin repeat domains 2
1.15	0.0019189000	3.522	225402_at	TP53RK	TP53 regulating kinase
0.95	0.0019222000	-3.522	202482_x_at	RANBP1	RAN binding protein 1
0.95	0.0019465000	-3.516	239781_at	NA	NA
1.2	0.0019486000	3.516	201758_at	TSG101	tumor susceptibility gene 101
0.94	0.0019785000	-3.509	204078_at	LEPREL4	leprecan-like 4
0.92	0.0019982000	-3.505	206960_at	LPAR4	lysophosphatidic acid receptor 4
0.96	0.0019985000	-3.505	219902_at	BHMT2	betaine--homocysteine S-methyltransferase 2
1.23	0.0019988000	3.505	241036_at	NA	NA
0.94	0.0020083000	-3.503	224633_s_at	GPATCH4	G patch domain containing 4
1.18	0.0020218000	3.500	244482_at	NA	NA
1.08	0.0020469000	3.495	228899_at	LOC100132884	hypothetical protein LOC100132884
1.07	0.0020473000	3.495	210189_at	HSPA1L	heat shock 70kDa protein 1-like
0.94	0.0020475000	-3.495	234429_at	LOC100506667	hypothetical protein LOC100506667
0.93	0.0020503000	-3.495	229324_x_at	ISYNA1	inositol-3-phosphate synthase 1
0.93	0.0020541000	-3.494	238823_at	FMNL3	formin-like 3
0.95	0.0020770000	-3.489	240171_at	NA	NA
1.05	0.0020773000	3.489	205410_s_at	ATP2B4	ATPase, Ca++ transporting, plasma membrane 4
0.95	0.0020799000	-3.489	227218_at	RLTPR	RGD motif, leucine rich repeats, tropomodulin domain and proline-rich containing
0.94	0.0020827000	-3.488	235845_at	SP5	Sp5 transcription factor
1.22	0.0021115000	3.482	223090_x_at	VEZT	vezatin, adherens junctions transmembrane protein
0.96	0.0021149000	-3.482	1555580_at	NA	NA
1.07	0.0021783000	3.469	202618_s_at	MECP2	methyl CpG binding protein 2 (Rett syndrome)
0.83	0.0022301000	-3.460	224870_at	KIAA0114	KIAA0114
1.07	0.0022315000	3.459	203950_s_at	CLCN6	chloride channel 6
0.95	0.0022337000	-3.459	1555027_at	P4HA2	prolyl 4-hydroxylase, alpha polypeptide II
0.94	0.0022512000	-3.456	211532_x_at	KIR2DS2	killer cell immunoglobulin-like receptor, two domains, short cytoplasmic tail, 2
0.95	0.0023512000	-3.437	243392_at	USP49	ubiquitin specific peptidase 49
1.07	0.0023610000	3.436	48612_at	N4BP1	NEDD4 binding protein 1
1.04	0.0023912000	3.430	1556261_a_at	NA	NA
1.3	0.0023980000	3.429	201651_s_at	PACSIN2	protein kinase C and casein kinase substrate in neurons 2
0.94	0.0024603000	-3.418	229227_at	FLJ45244	hypothetical locus FLJ45244
0.94	0.0024713000	-3.417	240797_at	PERP	PERP, TP53 apoptosis effector
0.94	0.0024844000	-3.414	220554_at	SLC22A7	solute carrier family 22 (organic anion transporter), member 7
0.93	0.0024931000	-3.413	203027_s_at	MVD	mevalonate (diphospho) decarboxylase
0.96	0.0025019000	-3.411	230744_at	FSTL1	follistatin-like 1
0.94	0.0025129000	-3.410	216661_x_at	CYP2C9	cytochrome P450, family 2, subfamily C, polypeptide 9
0.92	0.0025212000	-3.408	239522_at	IL12RB1	interleukin 12 receptor, beta 1
0.94	0.0025215000	-3.408	206598_at	INS	insulin
1.11	0.0025233000	3.408	203160_s_at	RNF8	ring finger protein 8
0.94	0.0025314000	-3.407	217361_at	NA	NA
1.04	0.0025575000	3.402	236563_at	RD3	retinal degeneration 3
1.14	0.0025696000	3.400	202128_at	KIAA0317	KIAA0317
1.34	0.0025869000	3.397	218092_s_at	AGFG1	ArfGAP with FG repeats 1
0.94	0.0025890000	-3.397	215594_at	NA	NA
1.06	0.0026391000	3.389	234346_x_at	NA	NA
0.93	0.0026620000	-3.385	202366_at	ACADS	acyl-CoA dehydrogenase, C-2 to C-3 short chain
1.05	0.0027098000	3.378	233844_at	CD99L2	CD99 molecule-like 2
0.93	0.0027371000	-3.374	1564066_at	TMEM151B	transmembrane protein 151B
0.94	0.0027548000	-3.371	217135_x_at	NA	NA
0.95	0.0027639000	-3.370	223992_x_at	ZCWPW1	zinc finger, CW type with PWWP domain 1
0.93	0.0028235000	-3.361	230297_x_at	SYNGAP1	synaptic Ras GTPase activating protein 1
0.95	0.0028319000	-3.359	1562004_x_at	MYOZ3	myozenin 3
1.09	0.0028390000	3.358	221980_at	EMILIN2	elastin microfibril interfacer 2

Supplementary Table 3 List of 306 probe-sets of the classifier that discrminates between initial and relapse AML samples, ranked according to *P-value*					
**Geometric mean of intensities relapse/diagnosis**	**Parametric p-value**	**t-value**	**ProbeSet**	**Symbol**	**Name**
1.08	0.0028641000	3.355	203644_s_at	MON1B	MON1 homolog B (yeast)
1.07	0.0028650000	3.355	244578_at	LCP2	lymphocyte cytosolic protein 2 (SH2 domain containing leukocyte protein of 76kDa)
1.15	0.0028723000	3.354	229773_at	SNAP23	synaptosomal-associated protein, 23kDa
1.25	0.0028852000	3.352	208901_s_at	TOP1	topoisomerase (DNA) I
0.96	0.0028886000	-3.351	1554405_a_at	NCRNA00161	non-protein coding RNA 161
1.33	0.0029007000	3.349	201089_at	ATP6V1B2	ATPase, H+ transporting, lysosomal 56/58kDa, V1 subunit B2
1.06	0.0029060000	3.349	210990_s_at	LAMA4	laminin, alpha 4
0.88	0.0029108000	-3.348	218051_s_at	NT5DC2	5'-nucleotidase domain containing 2
0.94	0.0029123000	-3.348	229880_at	NA	NA
1.15	0.0029697000	3.340	215017_s_at	FNBP1L	formin binding protein 1-like
1.06	0.0029845000	3.337	226122_at	PLEKHG1	pleckstrin homology domain containing, family G (with RhoGef domain) member 1
1.12	0.0029895000	3.337	1559490_at	LRCH3	leucine-rich repeats and calponin homology (CH) domain containing 3
0.94	0.0029993000	-3.335	232869_at	SRGAP3	SLIT-ROBO Rho GTPase activating protein 3
0.9	0.0030077000	-3.334	211052_s_at	TBCD	tubulin folding cofactor D
1.1	0.0030322000	3.331	239842_x_at	NA	NA
1.13	0.0030334000	3.331	229795_at	NA	NA
1.18	0.0030526000	3.328	205664_at	KIN	KIN, antigenic determinant of recA protein homolog (mouse)
1.13	0.0031164000	3.319	238589_s_at	NA	NA
0.95	0.0031198000	-3.319	236457_at	GTDC1	glycosyltransferase-like domain containing 1
1.38	0.0031287000	3.318	231735_s_at	MALAT1	metastasis associated lung adenocarcinoma transcript 1 (non-protein coding)
1.35	0.0031321000	3.317	207785_s_at	RBPJ	recombination signal binding protein for immunoglobulin kappa J region
1.7	0.0031428000	3.316	210387_at	NA	NA
1.09	0.0031639000	3.313	1557477_at	NA	NA
1.13	0.0031706000	3.312	241936_x_at	NA	NA
0.81	0.0031767000	-3.311	43511_s_at	ARRB1	arrestin, beta 1
0.94	0.0031809000	-3.311	217438_at	NA	NA
0.97	0.0031951000	-3.309	1553846_at	SPERT	spermatid associated
0.69	0.0032024000	-3.308	238365_s_at	C1orf228	chromosome 1 open reading frame 228
0.95	0.0032345000	-3.304	231196_x_at	NCRNA00202	non-protein coding RNA 202
1.1	0.0032413000	3.303	223118_s_at	USP47	ubiquitin specific peptidase 47
1.08	0.0032527000	3.301	217928_s_at	PPP6R3	protein phosphatase 6, regulatory subunit 3
1.12	0.0032721000	3.299	218532_s_at	FAM134B	family with sequence similarity 134, member B
1.14	0.0032892000	3.297	232094_at	C15orf29	chromosome 15 open reading frame 29
0.96	0.0032903000	-3.296	236020_s_at	TRUB1	TruB pseudouridine (psi) synthase homolog 1 (E. coli)
1.07	0.0032912000	3.296	241827_at	ZNF615	zinc finger protein 615
1.09	0.0033460000	3.289	207292_s_at	MAPK7	mitogen-activated protein kinase 7
0.94	0.0033555000	-3.288	218550_s_at	LRRC20	leucine rich repeat containing 20
1.04	0.0033592000	3.288	228229_at	ZNF526	zinc finger protein 526
1.07	0.0033685000	3.286	1570135_at	ZNF230	zinc finger protein 230
0.96	0.0033771000	-3.285	232191_at	C21orf105	chromosome 21 open reading frame 105
0.93	0.0033781000	-3.285	215695_s_at	GYG2	glycogenin 2
1.22	0.0033835000	3.285	222763_s_at	WDR33	WD repeat domain 33
1.05	0.0034195000	3.280	1553466_at	CXorf59	chromosome X open reading frame 59
1.18	0.0034238000	3.280	226840_at	H2AFY	H2A histone family, member Y
0.89	0.0034502000	-3.276	205339_at	STIL	SCL/TAL1 interrupting locus
1.1	0.0034564000	3.276	213405_at	RAB22A	RAB22A, member RAS oncogene family
1.07	0.0034618000	3.275	216000_at	NA	NA
1.12	0.0034662000	3.274	221502_at	KPNA3	karyopherin alpha 3 (importin alpha 4)
0.94	0.0034675000	-3.274	211457_at	GABARAPL3	GABA(A) receptors associated protein like 3, pseudogene
1.06	0.0035007000	3.270	215053_at	SRCAP	Snf2-related CREBBP activator protein
1.33	0.0035071000	3.269	223577_x_at	MALAT1	metastasis associated lung adenocarcinoma transcript 1 (non-protein coding)
0.95	0.0035254000	-3.267	227097_at	ZNF800	zinc finger protein 800
1.21	0.0035546000	3.264	208865_at	CSNK1A1	casein kinase 1, alpha 1
0.93	0.0035552000	-3.264	214903_at	SYT2	synaptotagmin II

Geometric mean of intensities relapse/diagnosis	Parametric p-value	t-value	ProbeSet	Symbol	Name
0.93	0.0035686000	-3.262	220838_at	EXD3	exonuclease 3'-5' domain containing 3
0.94	0.0035748000	-3.261	215425_at	BTG3	BTG family, member 3
1.15	0.0035902000	3.260	225121_at	TBC1D23	TBC1 domain family, member 23
1.23	0.0035950000	3.259	208943_s_at	SEC62	SEC62 homolog (S. cerevisiae)
0.86	0.0035966000	-3.259	230481_at	ACY3	aspartoacylase (aminocyclase) 3
0.92	0.0035971000	-3.259	214797_s_at	CDK18	cyclin-dependent kinase 18
0.94	0.0036034000	-3.258	208401_s_at	GLP1R	glucagon-like peptide 1 receptor
1.62	0.0036165000	3.256	209398_at	HIST1H1C	histone cluster 1, H1c
1.14	0.0036331000	3.255	211385_x_at	SULT1A2	sulfotransferase family, cytosolic, 1A, phenol-preferring, member 2
0.93	0.0036469000	-3.253	238229_at	TMEM67	transmembrane protein 67
1.1	0.0036576000	3.252	207122_x_at	SULT1A2	sulfotransferase family, cytosolic, 1A, phenol-preferring, member 2
1.05	0.0036588000	3.252	238819_at	ZNF347	zinc finger protein 347
1.2	0.0036720000	3.250	205474_at	CRLF3	cytokine receptor-like factor 3
1.05	0.0036965000	3.247	1555348_at	TFAP2E	transcription factor AP-2 epsilon (activating enhancer binding protein 2 epsilon)
0.95	0.0037171000	-3.245	1557357_at	LOC440944	hypothetical LOC440944
1.08	0.0037332000	3.243	220610_s_at	LRRFIP2	leucine rich repeat (in FLII) interacting protein 2
0.92	0.0037391000	-3.242	214174_s_at	PDLIM4	PDZ and LIM domain 4
1.47	0.0037577000	3.240	201743_at	CD14	CD14 molecule
0.95	0.0037786000	-3.238	244231_at	NA	NA
0.9	0.0037794000	-3.238	201709_s_at	NIPSNAP1	nipsnap homolog 1 (C. elegans)
0.95	0.0038120000	-3.234	220820_at	NA	NA
1.17	0.0038171000	3.234	201567_s_at	GOLGA4	golgin A4
1.19	0.0038278000	3.232	223398_at	C9orf89	chromosome 9 open reading frame 89
1.05	0.0038482000	3.230	213580_at	CREBZF	CREB/ATF bZIP transcription factor
1.13	0.0038488000	3.230	242990_at	NA	NA
1.13	0.0038596000	3.229	221497_x_at	EGLN1	egl nine homolog 1 (C. elegans)
0.94	0.0038601000	-3.229	219986_s_at	ACAD10	acyl-CoA dehydrogenase family, member 10
0.96	0.0039270000	-3.222	1570529_at	KIAA0020	KIAA0020
0.94	0.0039343000	-3.221	239257_at	MOV10L1	Mov10l1, Moloney leukemia virus 10-like 1, homolog (mouse)
0.94	0.0039403000	-3.220	203695_s_at	DFNA5	deafness, autosomal dominant 5
1.04	0.0039565000	3.218	215564_at	AREG	amphiregulin
1.13	0.0039779000	3.216	1568780_at	LOC649305	hypothetical LOC649305
1.14	0.0039901000	3.215	204507_s_at	PPP3R1	protein phosphatase 3, regulatory subunit B, alpha
0.95	0.0039918000	-3.215	204685_s_at	ATP2B2	ATPase, Ca++ transporting, plasma membrane 2
1.14	0.0040012000	3.214	232344_at	NA	NA
0.96	0.0040018000	-3.214	207825_s_at	GHRHR	growth hormone releasing hormone receptor
1.07	0.0040258000	3.211	243206_at	NA	NA
1.51	0.0040535000	3.208	232629_at	PROK2	prokineticin 2
0.97	0.0040686000	-3.207	1552995_at	IL27	interleukin 27
0.96	0.0040774000	-3.206	231986_at	RIMS1	regulating synaptic membrane exocytosis 1
1.18	0.0040817000	3.205	212112_s_at	STX12	syntaxin 12
1.08	0.0040915000	3.204	1562848_at	NA	NA
0.95	0.0040956000	-3.204	1567036_at	C20orf181	chromosome 20 open reading frame 181
0.97	0.0040998000	-3.203	1556554_at	TRIM50	tripartite motif containing 50
0.95	0.0041361000	-3.200	1554203_at	GRIK1-AS1	GRIK1 antisense RNA 1 (non-protein coding)
0.96	0.0041515000	-3.198	215697_at	NA	NA
1.06	0.0041522000	3.198	214928_at	OBSL1	obscurin-like 1
1.04	0.0041620000	3.197	216512_s_at	DCT	dopachrome tautomerase (dopachrome delta-isomerase, tyrosine-related protein 2)
0.94	0.0041796000	-3.195	237652_at	NA	NA
1.07	0.0042093000	3.192	1569940_at	SLC6A16	solute carrier family 6, member 16
1.15	0.0042305000	3.190	217552_x_at	CR1	complement component (3b/4b) receptor 1 (Knops blood group)
0.88	0.0042343000	-3.190	227859_at	DNAJC27	DnaJ (Hsp40) homolog, subfamily C, member 27
0.92	0.0042437000	-3.189	230449_x_at	NA	NA
1.36	0.0042869000	3.184	200958_s_at	SDCBP	syndecan binding protein (syntenin)
0.95	0.0044171000	-3.172	223549_s_at	ESPN	espin

Geometric mean of intensities relapse/diagnosis	Parametric p-value	t-value	ProbeSet	Symbol	Name
0.95	0.0044683000	-3.167	225587_at	TMEM129	transmembrane protein 129
1.07	0.0045035000	3.163	222887_s_at	TMEM127	transmembrane protein 127
1.48	0.0045083000	3.163	205633_s_at	ALAS1	aminolevulinate, delta-, synthase 1
1.05	0.0045097000	3.163	221631_at	CACNA1I	calcium channel, voltage-dependent, T type, alpha 1I subunit
1.05	0.0045188000	3.162	235896_s_at	SMCR7	Smith-Magenis syndrome chromosome region, candidate 7
1.11	0.0045202000	3.162	1555789_s_at	PHF23	PHD finger protein 23
0.96	0.0045433000	-3.160	230317_x_at	TMCO3	transmembrane and coiled-coil domains 3
0.94	0.0045727000	-3.157	212559_at	PRKAR1B	protein kinase, cAMP-dependent, regulatory, type I, beta
0.95	0.0045990000	-3.154	223801_s_at	APOL4	apolipoprotein L, 4
0.89	0.0046388000	-3.151	221951_at	TMEM80	transmembrane protein 80
1.06	0.0046455000	3.150	223255_at	G2E3	G2/M-phase specific E3 ubiquitin protein ligase
1.17	0.0046853000	3.147	210423_s_at	SLC11A1	solute carrier family 11 (proton-coupled divalent metal ion transporters), member 1
1.21	0.0046885000	3.146	203037_s_at	MTSS1	metastasis suppressor 1
0.95	0.0046981000	-3.145	237227_at	NEK10	NIMA (never in mitosis gene a)- related kinase 10
0.97	0.0046995000	-3.145	234962_at	NA	NA
1.13	0.0047098000	3.144	218364_at	LRRFIP2	leucine rich repeat (in FLII) interacting protein 2
0.96	0.0047219000	-3.143	207669_at	KRT83	keratin 83
1.04	0.0047418000	3.141	203019_x_at	SSX2IP	synovial sarcoma, X breakpoint 2 interacting protein
1.08	0.0047571000	3.140	239034_at	CXorf24	chromosome X open reading frame 24
0.95	0.0047617000	-3.140	234222_at	NA	NA
0.94	0.0047672000	-3.139	209767_s_at	NA	NA
1.09	0.0047716000	3.139	230209_at	ZXDC	ZXD family zinc finger C
0.92	0.0047739000	-3.139	241633_x_at	NA	NA
0.94	0.0047860000	-3.137	235689_at	MTFMT	mitochondrial methionyl-tRNA formyltransferase
1.09	0.0048285000	3.134	229970_at	NA	NA
1.03	0.0048365000	3.133	234589_at	TMEM106A	transmembrane protein 106A
1.08	0.0048917000	3.128	206613_s_at	TAF1A	TATA box binding protein (TBP)-associated factor, RNA polymerase I, A, 48kDa
1.06	0.0049048000	3.127	238548_at	NA	NA
1.09	0.0049205000	3.126	1564064_a_at	ATP11B	ATPase, class VI, type 11B
1.07	0.0049364000	3.124	1562244_at	ZNF578	zinc finger protein 578
1.3	0.0049636000	3.122	216942_s_at	CD58	CD58 molecule
