# Supplementary material for: Gene Expression Profiles Associated with Pediatric Relapsed AML
Source: PLoS One. 2015 Apr 7;10(4):e0121730. doi: 10.1371/journal.pone.0121730 (PMC4388534; doi:10.1371/journal.pone.0121730)
Supplement: S4 Table — (PDF) [file pone.0121730.s007.pdf]

Supplementary Table S4. Ingenuity transcription factor analysis output

Pair 1

Transcription Regulator	p-value of overlap	Target molecules in dataset
CEBPA	2.50E-06	BTG1,CA2,CCND2,CXCR4,GATA2,ID2,KLRC1,LITAF,MPO,NFIL3,RAB31,SERPINB1,TUBB2A
SATB1	3.18E-05	ACTG1,CD69,HBB,HSP90AA1,HSPA8,NR4A2,PIK3IP1,PRKCB,SELL,SLC27A2,TSC22D3
ELF4	1.46E-04	CA2,HDC,IL8,KIT,RAP1B
GATA2	7.15E-04	PECAM1,PRG2,RNASE2
GATA1	1.11E-03	HBB,LMO2,PRG2,RNASE2
CYLD	2.33E-03	IL8,JUN
GTF2B	2.33E-03	NFKBIA,TNFAIP3
TAF4B	4.58E-03	NFKBIA,TNFAIP3
KLF2	7.86E-03	CCL3,IL8,SELL
GFI1	1.32E-02	IL6R,IL8,RB1
SP1	1.41E-02	CAT,HBB,HDC,KIT,PIM1
RELA	1.48E-02	IL8,KIT,NFKBIA,PTPN6
NFE2	1.98E-02	HBB,TXN (includes EG:116484)
FOXN2	2.82E-02	HBB
NR1H3	2.82E-02	ABCA1
TFAP2A	2.82E-02	ABCA1
HLTF	2.82E-02	HBB
MED7 (includes EG:171285)	2.82E-02	TNFAIP3
MTA2	2.82E-02	HBB
MAFK	2.82E-02	TXN (includes EG:116484)
GTF3A	2.82E-02	PIM1
BTG1	2.82E-02	NR3C1
BACH1	2.82E-02	HBB
MED21	2.82E-02	TNFAIP3
KLF13	2.82E-02	CCL5
MYC	2.85E-02	CCND2,HNRNPA1,HSP90AA1,NAP1L1,PMAIP1
TBP	3.07E-02	NFKBIA,TNFAIP3

Pair 2

Transcription Regulator	p-value of overlap	Target molecules in dataset
CEBPA	8.96E-07	ACSL1,ALOX5AP,BCL2A1,BTG1,BTG2,CCND2,CXCR4,EXTL2,GCH1,GLIPR1,H1FX,HLA-B,ID2,ISG15,ITGAM (includes EG:16409), LITAF,MPO,NFIL3,PRTN3,PTPRC,RGS2 (includes EG:19735),S100A9,SERPINB1,SOD2,TANK,TUBB2A
IRF7	1.12E-06	BCL2L13,CXCL10,DNAJA1,IFI44,IFI44L,IFIH1,IFIT1,IFIT2,IFIT3,IFNB1 (includes EG:15977),IRF1 (includes EG:16362),IRF9,ISG15,ISG20,MCL1,MX1,NAMPT,NMI,OASL,PELI1,PMAIP1,RIPK2,RSAD2,S100A8,TNFSF13B,ZC3HAV1
IRF3	1.21E-06	ANXA4,CD58,IFI44,IFIT1,IFIT2,IFIT3,IFNB1 (includes EG:15977),ISG15,NR3C1,PMAIP1,RASA1,RSAD2,SORL1,TMPO,TNF
HMGB1	4.94E-06	CCL3,CCL4,CD58,CD83,HLA-DRB1,ICAM1,IL8,MRC1 (includes EG:100286774),TNF
TBP	7.91E-06	BCL2,EIF4E,HLA-A,IER3,NFKBIA,TNF,TNFAIP3
MYC	5.30E-05	CCND2,CDK4,CDKN1A,CSDE1,DLEU2,EXOSC8,FTH1 (includes EG:14319),GADD45A,HNRNPA1,HSP90AA1,HSPH1,MTHFD1, PHB,PMAIP1,SKP2 (includes EG:27401),TFRC,TYMS
SATB1	6.45E-04	ACTG1,ACTN1,CEACAM1 (includes others),DNMT3A,ENOSF1,FAM129A,GADD45B,HBB,HSP90AA1,HSPA8,IRF7,NR4A2, PTGS2,RGS1,SELL,SGK1,TSC22D3,UHRF1,VPS37B
TLX1	8.10E-04	MCL1,MYB,MYCBP,NMI,PCNA
STAT3	8.11E-04	CD83,CXCL10,FCER1G,FOS,IRF1 (includes EG:16362),MCL1,PTGS2,SOCS3,STAT3,TIMP1,TNF,VCAN
GFI1	1.03E-03	AZU1,CDKN1A,ELANE,ETS2,IL8,RB1,TNF
TP53 (includes EG:22059)	1.16E-03	BCL2,BIRC5,CDKN1A,DHFR,EGFR,HSP90AB1,PCNA,SLC19A1,UNC5B,VCAN
KLF2	2.02E-03	CCL3,CCL4,IL8,PTGS2,SELL,TNF
JUN	2.97E-03	APP,CDK1,FTH1 (includes EG:14319),PTGS2,TNF,VEGFA
TAF4B	3.49E-03	IER3,NFKBIA,TNFAIP3
AHR	4.21E-03	CCNG2,CD38,CDKN1A,GADD45A,ITGAM (includes EG:16409),PPP1R15A
RELA	4.46E-03	BCL2,IER3,IFNB1 (includes EG:15977),IL8,NFKB1,NFKBIA,PTGS2,TGFB1 (includes EG:21803),TNF
BRCA1	4.67E-03	CCNB2,H2AFX,H3F3A/H3F3B,SFPQ
RARA	7.75E-03	CD38,CDKN1A,JUP,RARA
MAX	8.47E-03	CCND2,CSDE1,DLEU2,GADD45A,MTHFD1
SRF	9.62E-03	FOS,MCL1
ZFP36	9.62E-03	CDKN1A,FOS
HIF1A	9.62E-03	TGFB1 (includes EG:21803),VEGFA
KAT2B	9.62E-03	PTGS2,TNF
ELK1	9.62E-03	FOS,MCL1
CEBPB	1.31E-02	ALOX5AP,IL8,MBP,PRG2,PRTN3,TNF

(includes EG:1051)		
E2F1	1.50E-02	CDK1,CDK4,TGFB1 (includes EG:21803)
GATA1	2.04E-02	FCER1A,HBB,MBP,PRG2,RNASE2
IRF8	2.40E-02	BCL6,CCR7,CD83,ICAM1
GTF2B	2.70E-02	NFKBIA,TNFAIP3
ELF4	3.26E-02	CBX1,CDKN1A,CREM,CXCL2,IL8
CEBPE	3.62E-02	ALOX5AP,ELANE,MBP
EP300	3.62E-02	HSP90AB1,NR4A1,TNF

Pair 3

Transcription Regulator	p-value of overlap	Target molecules in dataset
MYC	2.48E-05	APEX1,CDK4,CSDE1,HNRNPA1,HSP90AA1,HSPB1,NAP1L1,NBN,PHB,TFRC,TYMS,UXT
CEBPA	1.52E-04	ANXA1,BTG1,CEBPA,CSF3R,GLIPR1,H1FX,HLA-B,ITGAM (includes EG:16409),MPO,NFIL3,PRTN3,PTPRC,SOD2,TANK
SATB1	8.18E-04	ACTN1,HBB,HSP90AA1,HSPA8,IRF8,PTGES3,RGS1,RPLP0,RPLP1,TRIM22,TSC22D3,TUBA4A
BRCA1	5.02E-03	H2AFX,H3F3A/H3F3B,SFPQ
RB1	6.57E-03	HIST1H2AB/HIST1H2AE,TGFB1 (includes EG:21803)
REL	7.26E-03	IER3,IL8,SOD2
GFI1	9.12E-03	AZU1,CEBPA,ELANE,IL8
TBP	1.00E-02	HLA-A,IER3,TNFAIP3
TAF4B	1.27E-02	IER3,TNFAIP3
CEBPD	1.33E-02	CSF3R,ITGAM (includes EG:16409),PRTN3
SPI1 (includes EG:20375)	2.53E-02	CTSS,NCF1C,NCF2,PRTN3
E2F1	2.99E-02	CDK4,TGFB1 (includes EG:21803)
SP1	3.32E-02	ALOX5,CAT,GNAI2,HBB,MCL1,NCF2
PPARA	4.05E-02	NCF1C,NCF2
AHR	4.43E-02	CASP8,CCNG2,ITGAM (includes EG:16409)
FOXN2	4.76E-02	HBB
HLTF	4.76E-02	HBB
PLAGL2	4.76E-02	NCF2
MED7 (includes EG:171285)	4.76E-02	TNFAIP3
MTA2	4.76E-02	HBB
KEAP1	4.76E-02	GNAI2
BACH1	4.76E-02	HBB
MED21	4.76E-02	TNFAIP3

Pair 4

Transcription Regulator	p-value of overlap	Target molecules in dataset
CEBPA	2.16E-07	ACSL1,ANXA1,BCL2A1,BTG1,C3AR1,CD14,GCH1,GLIPR1,H1FX,HCAR3,HLA-B,ID2,IL1RN,LITAF,MPO,PTX3,RGS2 (includes EG:19735),S100A9,SERPINB1,SERPINF1,SOD2,TANK,TRIB1,TUBB2A,UBE2I
HMGB1	1.82E-05	CCL3,CCL4,CD83,ICAM1,IL1B,IL6,IL8,TNF
STAT3	2.15E-04	CD83,FCER1G,FOS,IL6,IRF1 (includes EG:16362),MCL1,PTGS2,SOCS3,TIMP1,TNF,TNFRSF1B,VCAN
RELA	3.69E-04	IER3,IFNGR2,IL1B,IL1RN,IL8,NFKB1,NFKBIA,PTGS2,TLR2,TNF
TLX1	4.12E-04	MCL1,MYB,MYCBP,NMI,PCNA
TBP	7.66E-04	HLA-A,IER3,NFKBIA,TNF,TNFAIP3
KLF2	9.45E-04	CCL3,CCL4,IL1B,IL8,PTGS2,TNF
MYC	1.55E-03	CDKN1A,CYFIP2,EXOSC8,FTH1 (includes EG:14319),HIST1H4A (includes others),HNRNPA1,HSP90AA1,MTHFD1,NAP1L1, NCL,SERINC3,TMSB10/TMSB4X,TYMS
TP53 (includes EG:22059)	1.66E-03	CDKN1A,DHFR,EGFR,FAS,IL6,PCNA,RUNX1,TRIM22,VCAN
IRF8	2.08E-03	BCL6,CCR7,CD83,ICAM1,IL1B
TAF4B	2.29E-03	IER3,NFKBIA,TNFAIP3
ELF4	3.81E-03	CBX1,CDKN1A,CXCL2,IL8,KLF4,RAP1B
REL	4.60E-03	BCL2L11,IER3,IL8,SOD2
STAT1	4.87E-03	CD14,FOS,IL1B,IRF1 (includes EG:16362),ITGAX,PIM1,TNF
NR3C2 (includes EG:110784)	5.35E-03	ATP1B1,BCL2A1,BID
ATF2	5.35E-03	DUSP1,IL6,TNF
SRF	7.20E-03	FOS,MCL1
ZFP36	7.20E-03	CDKN1A,FOS
KAT2B	7.20E-03	PTGS2,TNF
ELK1	7.20E-03	FOS,MCL1
JUN	8.63E-03	APP,CDK1,FTH1 (includes EG:14319),PTGS2,TNF
NFKB1	9.23E-03	IER3,IFNGR2,IL1B,IL1RN,NFKB1,NFKBIA,TNF
SP1	9.62E-03	CAT,CDKN1A,HIST1H4A (includes others),ITGAX,MAT2A,MCL1,PIM1,SOAT1,TLR2,TNF
CREBBP	1.00E-02	PTGS2,TLR2,TNF
SPI1 (includes EG:20375)	1.31E-02	CSF2RB,CTSS,FLI1,IL1B,IL1RN,NCF1C
SATB1	1.44E-02	CLEC2B,ENOSF1,EVI2A,GADD45B,HSP90AA1,HSPA8,IKZF1,PTGS2,RGS1,RPLP1,SGK1,TAOK1,TRIM22,UHRF1
GFI1	1.47E-02	CDKN1A,IL8,RB1,SERPINA1,TNF
GTF2B	2.04E-02	NFKBIA,TNFAIP3
MZF1	2.04E-02	CD34,MYB

RORC	2.47E-02	IL1B,IL6,TNF
BRCA1	2.47E-02	CCNB2,H3F3A/H3F3B,SFPQ
NR3C1	3.85E-02	ANXA1,ATP1B1
PPARG	4.66E-02	ABCA1,CTSL1,IL1B

Pair 5

Transcription Regulator	p-value of overlap	Target molecules in dataset
CEBPA	8.67E-08	ACSL1,BTG1,CAMP,CEBPA,CXCR4,GCH1,GGH,HLA-B,ID2,IFI27,ISG15,ITGAL,ITGAM (includes EG:16409),MPO,RAB31, S100A9,SOD2,TANK,THBD
SATB1	1.16E-05	CD69,CEACAM1 (includes others),CLEC2B,CTSD,FAM129A,GATA3,HBB,HSP90AA1,IRF7,IRF8,RGS1,SELL,TRIM22,TUBA4A,XAF1
MYC	2.48E-05	APEX1,CCND3,CSDE1,FTH1 (includes EG:14319),HIST1H4A (includes others),HNRNPA1,HSP90AA1,HSPB1,ITGAL,SCPEP1, TMSB10/TMSB4X,TYMS
GATA1	8.90E-05	FCER1A,GATA3,HBB,LMO2,PRG2,RNASE2
GATA2	1.58E-04	GATA3,PECAM1,PRG2,RNASE2
SP1	5.95E-04	ALOX5,CAMP,HBB,HIST1H4A (includes others),IFNGR1,ITGAX,MAT2A,NCF2,SOAT1
STAT3	9.71E-04	FCER1G,FCGR1A,FOS,LILRB2,LILRB4,TIMP1,TNFRSF1B,VCAN
ELF1	9.95E-04	FCER1A,FCER1G,NCF2
IRF3	1.85E-03	AHNAK,B4GALT5,CCL5,CD58,ISG15,RASA1,SORL1
HIF1A	2.26E-03	TGFB1 (includes EG:21803),VEGFA
TP53 (includes EG:22059)	4.86E-03	BIRC5,EGFR,HSP90AB1,PTEN,TRIM22,VCAN
JUND	6.55E-03	FTH1 (includes EG:14319),NCF2
STAT1	6.84E-03	FCGR1A,FOS,IRF7,IRF8,ITGAX
KLF10	1.27E-02	GATA3,TGFB1 (includes EG:21803)
IRF7	1.57E-02	CCL5,DNAJA1,ISG15,MX1,NAMPT,PSME2,S100A8,TNFSF13B,TRIM22,XAF1
USF2	2.05E-02	B2M,GATA3
FOS	2.05E-02	FTH1 (includes EG:14319),NCF2
SPI1 (includes EG:20375)	2.52E-02	CTSS,NCF1C,NCF2,PTPN6
USF1	2.98E-02	B2M,GATA3
JUNB	2.98E-02	FTH1 (includes EG:14319),NCF2
STAT5B	2.98E-02	BCL6,FOS
JUN	3.77E-02	FTH1 (includes EG:14319),NCF2,VEGFA
HMGB1	3.77E-02	CCL3,CD58,HLA-DRB1
PPARA	4.04E-02	NCF1C,NCF2
AHR	4.42E-02	CCNG2,DUSP6,ITGAM (includes EG:16409)
FOXN2	4.75E-02	HBB
FOXD2	4.75E-02	PRKAR1A

SPIC	4.75E-02	FCGR2B
HLTF	4.75E-02	HBB
PLAGL2	4.75E-02	NCF2
HINFP	4.75E-02	HIST1H4A (includes others)
TRIM28	4.75E-02	S100A9
MTA2	4.75E-02	HBB
RBPJ	4.75E-02	CAMP
PIAS3	4.75E-02	FTH1 (includes EG:14319)
FOSB	4.75E-02	FTH1 (includes EG:14319)
BACH1	4.75E-02	HBB
KLF5	4.75E-02	BIRC5
KLF13	4.75E-02	CCL5

Pair 6

Transcription Regulator	p-value of overlap	Target molecules in dataset
GATA1	3.29E-05	AHSP,ALAS2,GATA3,HBB,MBP,MPL,RNASE2
CEBPA	1.70E-04	ANXA1,BTG1,CEBPA,CSF3R,H1FX,HLA-B,ID2,ISG15,LST1,MPO,PTX3,S100A9,SERPINB1,SOD2,TANK,TUBB2A
SATB1	2.06E-04	CD69,CTNNA1,DSTYK,EVI2A,GATA3,HBB,HSPA8,NR4A2,PRKCB,RASGRP2,SELL,SGK1,TAOK1,TUBA4A,UBE2L6
GFI1	3.54E-03	AZU1,CEBPA,ELANE,ETS2,RB1
CEBPE	9.82E-03	CSF3R,ELANE,MBP
EGR2	1.05E-02	CBLB,MBP
GTF2B	1.05E-02	NFKBIA,TNFAIP3
IRF7	1.28E-02	CCL5,IFITM1,ISG15,NAMPT,PELI1,PSMB10,PSME1,PSME2,S100A8,TNFAIP8,TNFSF13B,UBE2L6
TBP	1.92E-02	HLA-A,NFKBIA,TNFAIP3
NOTCH3	2.02E-02	ID1,PTX3
KLF10	2.02E-02	GATA3,TGFB1 (includes EG:21803)
TAF4B	2.02E-02	NFKBIA,TNFAIP3
NR3C1	2.02E-02	ANXA1,ATP1B1
RUNX1T1	3.23E-02	CD34,ELANE

Pair 7

Transcription Regulator	p-value of overlap	Target molecules in dataset
HMGB1	7.29E-14	CCL3,CCL4,CD58,CD83,CD86,HLA-DRB1,ICAM1,IL1B,IL6,IL8,MRC1 (includes EG:100286774),TNF

CEBPA	1.86E-12	ACSL1,ANXA1,BCL2A1,BTG1,C3AR1,CD14,CXCR4,GLIPR1,H1FX,HCAR3,HLA-B,HMOX1,IL1RN,ISG15,ITGAM (includes EG:16409), NFIL3,PTPRC,RGS2 (includes EG:19735),S100A9,SERPINB1,SOD2,TANK,THBD,TNFSF10
IRF7	1.77E-11	CARD16,CCL5,CXCL10,HERC5,IFI44,IFIT1,IFIT2,IFIT3,IFNB1 (includes EG:15977),IRF1 (includes EG:16362),ISG15,MCL1,MX1,NAMPT,OASL,PELI1,PMAIP1,RIPK2,S100A8,STAT1,TLR4,TNFAIP8,TNFSF10
STAT3	2.64E-10	CD83,CD86,CXCL10,FCER1G,FOS,IL6,IRF1 (includes EG:16362),LILRB2,MCL1,PTGS2,SOCS3,TIMP1,TNF,TNFRSF1B,VCAN
IRF3	3.97E-09	ADAM9,B4GALT5,CCL5,CD58,IFI44,IFIT1,IFIT2,IFIT3,IFNB1 (includes EG:15977),ISG15,PMAIP1,SORL1,TNF
KLF2	1.45E-06	CCL3,CCL4,IL1B,IL8,PTGS2,SELL,TNF
SP1	1.54E-06	ALOX5,CAT,CDKN1A,HBB,IFNGR1,ITGAX,MCL1,NCF2,PIM1,SOAT1,TLR2,TNF
IRF8	5.17E-06	BCL6,CCR7,CD83,CD86,ICAM1,IL1B
STAT1	1.03E-05	CD14,FOS,IL1B,IRF1 (includes EG:16362),IRF8,ITGAX,PIM1,TNF
SPI1 (includes EG:20375)	5.50E-05	CTSS,FLI1,IL1B,IL1RN,MBP,NCF1C,NCF2
AHR	5.74E-05	CCNG2,CDKN1A,DUSP6,FAS,ITGAM (includes EG:16409),PPP1R15A
RELA	8.75E-05	IER3,IFNB1 (includes EG:15977),IL1B,IL1RN,IL8,PTGS2,TLR2,TNF
MYC	2.97E-04	CDKN1A,CYFIP2,FTH1 (includes EG:14319),HSPB1,MDM2,NBN,NCL,PMAIP1,SCPEP1,TFRC
REL	3.89E-04	BCL2L11,IER3,IL8,SOD2
TP53 (includes EG:22059)	5.83E-04	CASP1,CDKN1A,FAS,IL6,MDM2,RUNX1,VCAN
TBP	6.26E-04	EIF4E,IER3,TNF,TNFAIP3
ATF2	7.89E-04	DUSP1,IL6,TNF
GFI1	8.56E-04	CDKN1A,IL8,RB1,SERPINA1,TNF
CEBPD	9.50E-04	CD14,IL1B,ITGAM (includes EG:16409),ITGAX
NFKB1	1.40E-03	IER3,IFNB1 (includes EG:15977),IL1B,IL1RN,TNF,TNFSF10
CREBBP	1.53E-03	PTGS2,TLR2,TNF
CEBPB (includes EG:1051)	1.82E-03	CD14,IL1B,IL8,MBP,TNF
SRF	1.93E-03	FOS,MCL1
ZFP36	1.93E-03	CDKN1A,FOS
XBP1 (includes EG:140614)	1.93E-03	HLA-DRA,IL6
KAT2B	1.93E-03	PTGS2,TNF
KLF4	1.93E-03	CD14,ITGAM (includes EG:16409)
ELK1	1.93E-03	FOS,MCL1
PPARA	2.59E-03	ABCA1,NCF1C,NCF2

RORC	4.00E-03	IL1B,IL6,TNF
SATB1	4.37E-03	GADD45B,HBB,HLA-DMB,IRF8,PTGS2,RGS1,SELL,SGK1,TAOK1,TSC22D3
JUN	4.40E-03	FTH1 (includes EG:14319),NCF2,PTGS2,TNF
JUND	5.61E-03	FTH1 (includes EG:14319),NCF2
MZF1	5.61E-03	CD34,MYB
FOXO3	5.61E-03	BCL2L11,TNFSF10
PPARG	8.03E-03	ABCA1,IL1B,TNFSF10
ELF4	8.44E-03	CDKN1A,CXCL2,IL8,KLF4
CREB1	1.07E-02	FOS,IL1B,TNF
TAF4B	1.09E-02	IER3,TNFAIP3
TP73	1.09E-02	CDKN1A,PMAIP1
ELF1	1.76E-02	FCER1G,NCF2
EGR1	1.76E-02	EGR1,TNF
FOS	1.76E-02	FTH1 (includes EG:14319),NCF2
IRF5	1.76E-02	IL6,TNF
BCL6	2.14E-02	BCL2A1,PRDM1,SOCS3
JUNB	2.57E-02	FTH1 (includes EG:14319),NCF2
STAT5B	2.57E-02	BCL6,FOS
VDR	3.49E-02	CD14,THBD
FOXN2	4.39E-02	HBB
HSF1 (includes EG:15499)	4.39E-02	HSPA1A/HSPA1B
NR1H3	4.39E-02	ABCA1
TFAP2A	4.39E-02	ABCA1
LITAF	4.39E-02	TNF
NFAT5	4.39E-02	TNF
HLTF	4.39E-02	HBB
PLAGL2	4.39E-02	NCF2
MED7 (includes EG:171285)	4.39E-02	TNFAIP3
TRIM28	4.39E-02	S100A9
MTA2	4.39E-02	HBB
PIAS3	4.39E-02	FTH1 (includes EG:14319)
BCL3	4.39E-02	TNF
GTF3A	4.39E-02	PIM1
FOSB	4.39E-02	FTH1 (includes EG:14319)
BACH1	4.39E-02	HBB
MED21	4.39E-02	TNFAIP3
NR1D1	4.39E-02	TLR4
SREBF2	4.39E-02	GTF2I
KLF13	4.39E-02	CCL5

CEBPE	4.52E-02	CEBPB (includes EG:1051),MBP
EP300	4.52E-02	TLR2,TNF

Pair 8

Transcription Regulator	p-value of overlap	Target molecules in dataset
CEBPA	3.65E-08	ANXA1,CSF3R,CXCR4,GLIPR1,H1FX,ICAM2,ID2,IFI27,MPO,MYC,RGS2 (includes EG:19735),S100A9,SERPINB1,SOD2,TANK, TRIB1,TUBB2A,VCL
IRF7	6.26E-06	CARD16,CCL5,IFI44,IFI44L,IFITM1,IFITM2,IFITM3,NMI,OAS3,PELI1,PMAIP1,PSMB9,S100A8,TNFSF13B,UBE2L6
SATB1	1.61E-04	CD69,CLEC2B,EPSTI1,EVI2A,FAM129A,HBB,HSP90AA1,HSPA8,RGS1,SELL,TSC22D3,UBE2L6
FOXP3	1.23E-03	ANXA1,ID2,MAF,NT5E,RGS1
CEBPB (includes EG:1051)	1.23E-03	CCND1,CSF3R,IL8,MYC,PRG2
MYC	2.93E-03	CSDE1,HSP90AA1,HSPB1,MFAP1,NAP1L1,PMAIP1,SERINC3,TMSB10/TMSB4X
IRF3	3.79E-03	AHNAK,ANXA4,CCL5,IFI44,PMAIP1,SORL1
AHR	4.05E-03	CCNG2,DUSP6,JUN,MYC
GATA1	4.05E-03	FCER1A,HBB,MPL,PRG2
CYLD	4.71E-03	IL8,JUN
NR3C1	9.17E-03	ANXA1,ATP1B1
ELF1	1.49E-02	FCER1A,FCER1G
STAT5B	2.17E-02	CCND1,MAF
GATA2	2.96E-02	PECAM1,PRG2
GFI1	3.36E-02	IL8,MYC,RB1
CEBPE	3.84E-02	CSF3R,MYC
CTNNB1	3.84E-02	CCND1,MYC
FOXN2	4.02E-02	HBB
FOXD2	4.02E-02	PRKAR1A
HSF1 (includes EG:15499)	4.02E-02	HSPA1A/HSPA1B
HLTF	4.02E-02	HBB
TRIM28	4.02E-02	S100A9
MTA2	4.02E-02	HBB
ENO1	4.02E-02	MYC
BACH1	4.02E-02	HBB
KLF13	4.02E-02	CCL5
REL	4.81E-02	IL8,SOD2

Pair 9

Transcription Regulator	p-value of overlap	Target molecules in dataset
CEBPA	8.94E-11	ACSL1,ANXA1,BCL2A1,BTG1,CXCR4,FOXO3,GCH1,GLIPR1,H1FX,HCAR3,ID2,IFI27,IL1RN,ISG15,LST1,MYC,NFIL3,PTX3, S100A9,SOD2,THBD,TNFSF10,TRD@,TUBB2A,UBE2I,VCL
HMGB1	1.90E-06	CCL3,CCL4,CD83,HLA-DRB1,ICAM1,IL1B,IL6,IL8
IRF3	2.03E-06	ADAM9,AHNAK,ANXA4,B4GALT5,CCL5,IFIT3,ISG15,PLCG2,PMAIP1,RASA1,SORL1,TMPO
TP53 (includes EG:22059)	4.17E-06	BCL2,CASP1,CDKN1A,FAS,HSP90AB1,IL6,PCNA,PTEN,RUNX1,SLC19A1,VCAN
GFI1	5.75E-06	AZU1,CDKN1A,ELANE,ETS2,IL6R,IL8,MYC,RB1
STAT3	1.12E-05	CD83,CXCL10,FCER1G,IL6,IRF1 (includes EG:16362),MCL1,MYC,SLAMF1,SOCS3,TIMP1,TNFRSF1B,VCAN
IRF7	2.48E-05	CCL5,CCNA1,CXCL10,IFIT3,IL4I1,IRF1 (includes EG:16362),ISG15,MCL1,MX1,NAMPT,OASL,PELI1,PMAIP1,PSMB8,RIPK2, S100A8,TNFAIP8,TNFSF10
TBP	1.85E-04	BCL2,IER3,MYC,NFKBIA,TNFAIP3
NFKB1	3.04E-04	IER3,IFNGR2,IL1B,IL1RN,NFKB1,NFKBIA,RELB,TNFSF10
MYC	3.44E-04	APEX1,CAD,CDKN1A,FTH1 (includes EG:14319),HSP90AA1,MINA,MTHFD1,NBN,PMAIP1,SCPEP1,SERINC3,TFRC
IRF8	5.23E-04	BCL6,CCR7,CD83,ICAM1,IL1B
ELF4	7.98E-04	CBX1,CDKN1A,CXCL2,IL8,KLF4,MT1G
PML	9.36E-04	CCNA1,RARA,SPI1 (includes EG:20375)
TAF4B	9.36E-04	IER3,NFKBIA,TNFAIP3
RELA	9.73E-04	BCL2,IER3,IFNGR2,IL1B,IL1RN,IL8,NFKB1,NFKBIA
REL	1.50E-03	BCL2L11,IER3,IL8,SOD2
RARA	1.50E-03	CCNA1,CDKN1A,RARA,THBD
KLF2	1.69E-03	CCL3,CCL4,IL1B,IL8,SELL
NR3C2 (includes EG:110784)	2.23E-03	BCL2,BCL2A1,BID
SPI1 (includes EG:20375)	3.02E-03	ETS2,FLI1,IL1B,IL1RN,NCF1C,NCF2
XBP1 (includes EG:140614)	3.92E-03	HLA-DRA,IL6
CTCF (includes EG:10664)	3.92E-03	HLA-DRB1,MYC
SP1	4.04E-03	CAT,CDKN1A,HBB,IFNGR1,MAT2B,MCL1,NCF2,PIM1,SLC19A1
PPARA	7.10E-03	ABCA1,NCF1C,NCF2
SATB1	7.19E-03	CDK19,GADD45B,HBB,HSP90AA1,IKZF1,RGS1,SELL,SLC27A2,SPI1 (includes EG:20375),TAOK1,TSC22D3,UHRF1
CEBPE	1.08E-02	CEBPB (includes EG:1051),ELANE,MYC

JUND	1.13E-02	FTH1 (includes EG:14319),NCF2
GTF2B	1.13E-02	NFKBIA,TNFAIP3
FOXO3	1.13E-02	BCL2L11,TNFSF10
TLX1	1.55E-02	MCL1,PCNA,RAF1
AHR	1.89E-02	CDKN1A,FAS,MYC,PPP1R15A
PPARG	2.11E-02	ABCA1,IL1B,TNFSF10
TP73	2.16E-02	CDKN1A,PMAIP1
SIN3A	2.16E-02	BCL2,HLA-DRA
RUNX1T1	3.46E-02	ELANE,SPI1 (includes EG:20375)
ELF1	3.46E-02	FCER1G,NCF2
FOXO1 (includes EG:2308)	3.46E-02	BCL2L11,KLF2
FOS	3.46E-02	FTH1 (includes EG:14319),NCF2
FOXP3	3.90E-02	ANXA1,ID2,NFIL3,RGS1
CEBPB (includes EG:1051)	3.90E-02	FOXO3,IL1B,IL8,MYC
JUNB	4.97E-02	FTH1 (includes EG:14319),NCF2

Pair 10

Transcription Regulator	p-value of overlap	Target molecules in dataset
CEBPA	3.51E-10	ACSL1,ANXA1,BTG1,CD14,CXCR4,H1FX,ITGAM (includes EG:16409),LITAF,MPO,MYC,RAB31,RGS2 (includes EG:19735), S100A9,SOD2,TNFSF10,TRIB1
STAT3	3.76E-08	CD83,FCER1G,FOS,LILRB2,MCL1,MYC,PTGS2,TIMP1,TNFRSF1B,VCAN
AHR	1.26E-06	CASP8,CCNG2,ITGAM (includes EG:16409),JUN,MYC,PPP1R15A
HMGB1	3.73E-04	CCL3,CD83,IL8,MRC1 (includes EG:100286774)
SRF	5.07E-04	FOS,MCL1
KLF4	5.07E-04	CD14,ITGAM (includes EG:16409)
ELK1	5.07E-04	FOS,MCL1
SATB1	7.09E-04	ACTN1,CD69,CLEC2B,FAM129A,HBB,NR4A2,PTGS2,RGS1
CYLD	1.50E-03	IL8,JUN
CEBPD	1.64E-03	CD14,ITGAM (includes EG:16409),MYC
KLF2	4.22E-03	CCL3,IL8,PTGS2
GFI1	7.21E-03	IL8,MYC,SERPINA1
CEBPB (includes EG:1051)	1.12E-02	CD14,IL8,MYC
NOTCH1	1.30E-02	ITGAM (includes EG:16409),MYC
REL	1.64E-02	IL8,SOD2
STAT1	2.01E-02	CD14,FOS,PIM1
TBP	2.02E-02	MYC,TNFAIP3
IRF7	2.14E-02	IFI16,MCL1,PMAIP1,S100A8,TNFSF10,TNFSF13B
FOXN2	2.25E-02	HBB
FOXD2	2.25E-02	PRKAR1A
HLTF	2.25E-02	HBB
MED7 (includes EG:171285)	2.25E-02	TNFAIP3
TRIM28	2.25E-02	S100A9
MTA2	2.25E-02	HBB
GTF3A	2.25E-02	PIM1
ENO1	2.25E-02	MYC
BACH1	2.25E-02	HBB
MED21	2.25E-02	TNFAIP3
SP1	2.76E-02	ALOX5,HBB,MCL1,PIM1
NCOR2	4.46E-02	CD69
ZFP36	4.46E-02	FOS
PYCARD	4.46E-02	DUSP10
CDKN2A	4.46E-02	CCND3

KAT2B	4.46E-02	PTGS2
FOXP1	4.46E-02	ITGAM (includes EG:16409)
KLF1	4.46E-02	HBB
CTCF (includes EG:10664)	4.46E-02	MYC
MYC	4.86E-02	CCND3,CDK4,MINA,PMAIP1
JUN	4.94E-02	JUN,PTGS2

Pair 11

Transcription Regulator	p-value of overlap	Target molecules in dataset
CEBPA	9.95E-06	BCL2A1,CA2,CXCR4,HLA-B,ID2,PRTN3,PTX3,RAB31,S100A9,SOD2
IRF7	6.98E-05	IFI44,IFI44L,IFIT3,IRF1 (includes EG:16362),MCL1,NAMPT,S100A8,STAT1,TNFAIP8
SRF	3.39E-04	FOS,MCL1
ELK1	3.39E-04	FOS,MCL1
REL	4.81E-04	IER3,IL8,SOD2
SATB1	1.05E-03	CLEC2B,GADD45B,NR4A2,P2RY8,PTGS2,RGS1,SELL
STAT3	1.24E-03	FOS,IRF1 (includes EG:16362),MCL1,PTGS2,SOCS3
KLF2	2.40E-03	IL8,PTGS2,SELL
GFI1	4.13E-03	AZU1,ELANE,IL8
STAT5B	4.84E-03	BCL6,FOS
HSF1 (includes EG:15499)	1.84E-02	HSPA1A/HSPA1B
TRIM28	1.84E-02	S100A9
RELA	2.44E-02	IER3,IL8,PTGS2
BCL6	2.67E-02	BCL2A1,SOCS3
RFX5	3.66E-02	HLA-DRA
ZFP36	3.66E-02	FOS
XBP1 (includes EG:140614)	3.66E-02	HLA-DRA
KAT2B	3.66E-02	PTGS2
ELF4	4.72E-02	CA2,IL8

Pair 12

Transcription Regulator	p-value of overlap	Target molecules in dataset
ELF4	1.49E-06	HDC,IL8,KIT,KLF4
GATA2	4.28E-04	PRG2,RNASE2
KLF2	2.09E-03	IL8,SELL
HMGB1	2.38E-03	IL8,MRC1 (includes EG:100286774)
GATA1	2.69E-03	PRG2,RNASE2

CEBPB (includes EG:1051)	4.11E-03	IL8,PRG2
ZBTB16	9.14E-03	KIT
PRDM1	9.14E-03	LGALS1
CEBPA	9.45E-03	GCH1,GLIPR1,RAB31
RELA	1.06E-02	IL8,KIT
CYLD	1.37E-02	IL8
SP1	2.28E-02	HDC,KIT
REL	4.05E-02	IL8

Pair 13

Transcription Regulator	p-value of overlap	Target molecules in dataset
SATB1	2.24E-04	EVI2A,FAM129A,GADD45B,HBB,HSP90AA1,IKZF1,IRF8,P2RY8,PRKCB,RGS1,TAOK1,TSC22D3,WARS
TBP	8.82E-04	BCL2,HLA-A,MYC,TNFAIP3
E2F1	1.98E-03	APAF1,CDK1,TGFB1 (includes EG:21803)
HIF1A	2.31E-03	TGFB1 (includes EG:21803),VEGFA
SP2	6.70E-03	GNAI2,MAT2A
IRF3	8.96E-03	CCL5,CD58,NR3C1,PMAIP1,SORL1,TMPO
JUN	3.88E-02	CDK1,JUN,VEGFA
IRF7	4.12E-02	CCL5,IFI16,IFI44L,IFITM1,NAMPT,PMAIP1,STAT1,TNFAIP8,TNFSF13B
AHR	4.55E-02	DUSP6,JUN,MYC
FOXN2	4.81E-02	HBB
FOXD2	4.81E-02	PRKAR1A
HSF1 (includes EG:15499)	4.81E-02	HSPA1A/HSPA1B
HLTF	4.81E-02	HBB
MED7 (includes EG:171285)	4.81E-02	TNFAIP3
HINFP	4.81E-02	HIST1H4A (includes others)
MTA2	4.81E-02	HBB
KEAP1	4.81E-02	GNAI2
ENO1	4.81E-02	MYC
BTG1	4.81E-02	NR3C1
BACH1	4.81E-02	HBB
MED21	4.81E-02	TNFAIP3
KLF13	4.81E-02	CCL5

Pair 14

Transcription Regulator	p-value of overlap	Target molecules in dataset
SATB1	1.68E-04	ACTN1,CEACAM1 (includes others),CLEC2B,EVI2A,FAM129A,GADD45B,HBB,HSP90AA1,HSPA8,NR4A2,VPS37B, VTA1 (includes EG:292640)
TLX1	2.82E-04	MCL1,MYB,MYCBP,PCNA
GATA1	4.36E-04	AHSP,HBB,LMO2,MBP,NFE2
SRF	1.63E-03	FOS,MCL1
ELK1	1.63E-03	FOS,MCL1
TP53 (includes EG:22059)	2.17E-03	APAF1,BCL2,CASP1,HSP90AB1,PCNA,RUNX1
IRF3	3.88E-03	ADAM9,CCL5,CD58,IFIT2,NR3C1,TMPO
MZF1	4.75E-03	CD34,MYB
CEBPA	4.96E-03	BTG1,BTG2,CXCR4,GJA1,H1FX,HLA-B,ICAM2,ID2,PTPRC,SOD2
IRF7	5.34E-03	CCL5,CCNA1,DNAJA1,GBP3,IFI16,IFIT2,MCL1,PELI1,TNFAIP8,TNFSF13B
PML	9.25E-03	CCNA1,JUP
E2F1	2.19E-02	APAF1,TGFB1 (includes EG:21803)
STAT5B	2.19E-02	BCL6,FOS
RUNX1	2.92E-02	JUP,NFE2,RUNX1
FOXN2	4.04E-02	HBB
FOXD2	4.04E-02	PRKAR1A
HSF1 (includes EG:15499)	4.04E-02	HSPA1A/HSPA1B
HLTF	4.04E-02	HBB
HINFP	4.04E-02	HIST1H4A (includes others)
MTA2	4.04E-02	HBB
PIAS3	4.04E-02	FTH1 (includes EG:14319)
FOSB	4.04E-02	FTH1 (includes EG:14319)
BTG1	4.04E-02	NR3C1
BACH1	4.04E-02	HBB
KLF13	4.04E-02	CCL5
RARA	4.85E-02	CCNA1,JUP

Pair 15

Transcription Regulator	p-value of overlap	Target molecules in dataset
SATB1	1.32E-06	ACTN1,CLEC2B,HBB,HSPA8,NR4A2,PIK3IP1,RGS1,TRIM22,TSC22D3
ELF4	3.03E-06	ABCB1,CA2,CXCL2,HDC,KLF4
SRF	1.58E-04	FOS,MCL1

ELK1	1.58E-04	FOS,MCL1
CEBPA	1.95E-04	ANXA1,CA2,GCH1,HCAR3,MPO,PTX3,RGS2 (includes EG:19735)
NOTCH3	9.30E-04	ID1,PTX3
JUN	9.74E-04	ABCB1,APP,JUN
TLX1	5.35E-03	MCL1,MYB
FOXN2	1.26E-02	HBB
HLTF	1.26E-02	HBB
MED7 (includes EG:171285)	1.26E-02	TNFAIP3
MTA2	1.26E-02	HBB
MBD1	1.26E-02	ABCB1
BACH1	1.26E-02	HBB
MED21	1.26E-02	TNFAIP3
AHR	1.89E-02	CD38,JUN
GATA1	1.89E-02	FCER1A,HBB
GFI1	2.11E-02	AZU1,ELANE
NFATC4	2.50E-02	APP
ZFP36	2.50E-02	FOS
MECP2	2.50E-02	ABCB1
KLF1	2.50E-02	HBB
KLF6	2.50E-02	COL1A1
SP1	2.63E-02	HBB,HDC,MCL1
FOXP3	2.83E-02	ANXA1,RGS1
IRF7	3.30E-02	IFI44,IFIT3,MCL1,TRIM22
CYLD	3.73E-02	JUN
GTF2B	3.73E-02	TNFAIP3
MZF1	3.73E-02	MYB
STAT5A	3.73E-02	FOS
SMARCA4	3.73E-02	HBB
TAF4B	4.94E-02	TNFAIP3
NR3C1	4.94E-02	ANXA1

Pair 16

Transcription Regulator	p-value of overlap	Target molecules in dataset
CEBPA	1.02E-05	BTG1,BTG2,CCND2,CEBPA,CXCR4,FOXO3,GATA2,H1FX,ICAM2,ID2,LITAF,LYL1,MPO,MYC,PRTN3,PTPRC, RGS2 (includes EG:19735),S100A9,SERPINB1,SOD2,TANK,TNFSF10,TRIB1,VCL
GFI1	1.46E-04	CEBPA,ELANE,GFI1B,IL6R,IL8,MYC,RB1,SERPINA1
SATB1	1.45E-03	ACTN1,CDK19,CLEC2B,DNMT3A,FAM129A,FAM65B,GADD45B,HSP90AA1,HSPA8,IKZF1,ILF3,NR4A2,P2RY8,PTGS2,RGS1, TAOK1,VTA1 (includes EG:292640),XAF1

SPI1 (includes EG:20375)	6.56E-03	CSF2RB,CTSS,FLI1,MBP,NCF4,PRTN3,PTPN6
ELF4	7.68E-03	CXCL2,IL8,KIT,PIK3C3,RAP1B,TAF7
IRF3	9.54E-03	AHNAK,B4GALT5,CCL5,NR3C1,PIK3C3,PMAIP1,RASA1,SORL1,TMPO
SRF	9.56E-03	FOS,MCL1
CDKN2A	9.56E-03	CCND3,NR3C1
ELK1	9.56E-03	FOS,MCL1
TBP	1.18E-02	BCL2,MYC,NFKBIA,TNFAIP3
MYC	1.39E-02	CCND2,CCND3,DLEU2,GADD45A,HIST1H4A (includes others),HSP90AA1,HSPH1,LAMP2,MFAP1,PMAIP1,SERINC3, SKP2 (includes EG:27401)
IRF7	2.35E-02	ADAR,CCL5,GBP3,IFI16,MCL1,NAMPT,PELI1,PMAIP1,S100A8,STAT1,STAT2,TNFAIP8,TNFSF10,TNFSF13B,TRIM5,XAF1
CYLD	2.68E-02	IL8,JUN
SP2	2.68E-02	MAT2A,MAT2B
GTF2B	2.68E-02	NFKBIA,TNFAIP3
SMAD4	2.68E-02	INPP5D,IRAK3
MZF1	2.68E-02	MS4A2,MYB
CEBPE	3.59E-02	ELANE,MBP,MYC
CEBPB (includes EG:1051)	4.80E-02	FOXO3,IL8,MBP,MYC,PRTN3

Pair 17

Transcription Regulator	p-value of overlap	Target molecules in dataset
CEBPA	2.64E-12	ANXA1,ARG1,BTG2,CAMP,CD14,CHI3L1,GBP1,HCAR3,HLA-B,ID2,ITGAL,LTF,PRTN3,PTX3,RGS2 (includes EG:19735), TRIB1,TUBB2A
MYC	1.99E-05	DDIT3,FTH1 (includes EG:14319),HSP90AA1,HSPB1,HSPH1,ITGAL,MIR17HG,PMAIP1
RELA	3.99E-05	IL1B,IL8,MMP9,NFKBIA,PTPN6,TGFB1 (includes EG:21803)
HMGB1	1.99E-04	CCL3,CD83,IL1B,IL8
GFI1	3.24E-04	AZU1,ELANE,IL8,SERPINA1
ELF4	4.05E-04	IL8,KLF4,MANF,PRF1
IRF7	5.04E-04	DNAJA1,GBP1,IFITM1,MX1,NAMPT,PELI1,PMAIP1,STAT1
CEBPB (includes EG:1051)	6.06E-04	CD14,IL1B,IL8,PRTN3
CEBPD	1.02E-03	CD14,IL1B,PRTN3
SPI1 (includes EG:20375)	1.03E-03	CHI3L1,IL1B,PRTN3,PTPN6
CYLD	1.08E-03	IL8,JUN
GTF2B	1.08E-03	NFKBIA,TNFAIP3
RB1	1.08E-03	HIST1H2AB/HIST1H2AE,TGFB1 (includes EG:21803)

SATB1	1.29E-03	CD69,GADD45B,HSP90AA1,HSPA8,RGS1,SGK1,SPI1 (includes EG:20375)
IRF8	1.34E-03	BCL6,CD83,IL1B
NOTCH3	2.13E-03	ID1,PTX3
TAF4B	2.13E-03	NFKBIA,TNFAIP3
NFKB1	2.13E-03	IL1B,MMP9,NFKBIA,PTPN6
KLF2	2.66E-03	CCL3,IL1B,IL8
RUNX1T1	3.51E-03	ELANE,SPI1 (includes EG:20375)
RUNX1	3.86E-03	CHI3L1,ITGAL,SPI1 (includes EG:20375)
FOXP3	7.13E-03	ANXA1,ID2,RGS1
CEBPE	9.45E-03	ELANE,LTF
PPARG	1.48E-02	IL1B,MMP9
TBP	1.48E-02	NFKBIA,TNFAIP3
SP1	1.62E-02	CAMP,CHI3L1,GNAI2,MMP9
NFE2L2	1.79E-02	FTH1 (includes EG:14319),GNAI2
HSF1 (includes EG:15499)	1.91E-02	HSPA1A/HSPA1B
MED7 (includes EG:171285)	1.91E-02	TNFAIP3
RBPJ	1.91E-02	CAMP
PIAS3	1.91E-02	FTH1 (includes EG:14319)
NKX3-1	1.91E-02	MIR17HG
BCLAF1	1.91E-02	TP53 (includes EG:22059)
KEAP1	1.91E-02	GNAI2
NOTCH2	1.91E-02	FCER2
FOSB	1.91E-02	FTH1 (includes EG:14319)
TCF7L2	1.91E-02	CD4
MED21	1.91E-02	TNFAIP3
SP3	2.85E-02	CHI3L1,GNAI2
JUN	3.66E-02	FTH1 (includes EG:14319),JUN
NCOR2	3.79E-02	CD69
RUNX3	3.79E-02	ITGAL
HIF1A	3.79E-02	TGFB1 (includes EG:21803)
HES1 (includes EG:15205)	3.79E-02	CD4
PYCARD	3.79E-02	IL1B
KLF4	3.79E-02	CD14
NFKBIB	3.79E-02	GNAI2
HLF	3.79E-02	LMO2
AHR	4.10E-02	DUSP6,JUN

**Pair 18**		
Transcription Regulator	p-value of overlap	Target molecules in dataset
HMGB1	7.81E-06	CCL3,CCL4,HLA-DRB1,ICAM1,IL1B,IL8,TNF
KLF2	6.64E-05	CCL3,CCL4,IL1B,IL8,SELL,TNF
TBP	7.76E-05	BCL2,IER3,NFKBIA,TNF,TNFAIP3
TAF4B	5.46E-04	IER3,NFKBIA,TNFAIP3
RELA	1.61E-03	BCL2,IER3,IL1B,IL8,LTB,NFKBIA,TNF
SATB1	1.65E-03	ACTG1,ACTN1,CLEC2B,EVI2A,FAM129A,GADD45B,HBB,PRKCB,PTGES3,RGS1,SELL,XAF1
GFI1	1.86E-03	CDKN1A,CEBPA,IL8,RB1,TNF
ELF4	2.42E-03	CDKN1A,CXCL2,IL8,KLF4,TAF7
ZFP36	2.72E-03	CDKN1A,FOS
CDKN2A	2.72E-03	CCND3,NR3C1
NFKB1	3.37E-03	IER3,IL1B,ITPR1,LTB,NFKBIA,TNF
FOXP3	3.88E-03	ANXA1,CYSLTR1,ID2,NFIL3,RGS1
CYLD	7.89E-03	IL8,JUN
GTF2B	7.89E-03	NFKBIA,TNFAIP3
HSF2	7.89E-03	HSPA1A/HSPA1B,TXN (includes EG:116484)
REL	9.41E-03	IER3,IL8,SOD2
AHR	1.02E-02	CCNG2,CDKN1A,DUSP6,JUN
SIN3A	1.52E-02	BCL2,HLA-DRA
CREB1	1.71E-02	FOS,IL1B,TNF
EGR1	2.45E-02	EGR1,TNF
ATF2	2.45E-02	DUSP1,TNF

**Pair 19**		
Transcription Regulator	p-value of overlap	Target molecules in dataset
CEBPA	6.59E-07	ANXA1,DYNLT3,FOXO3,HCAR3,ID2,LITAF,PRTN3,PTX3,S100A9,SERPINB1,TRIB1
IRF7	7.33E-07	IFITM1,IFITM2,ISG20,MCL1,NAMPT,PELI1,PMAIP1,RIPK2,S100A8,STAT1,TNFSF13B
TAF4B	1.95E-05	IER3,NFKBIA,TNFAIP3
CEBPB (includes EG:1051)	2.29E-05	FOXO3,IL1B,IL8,PRG2,PRTN3
HMGB1	1.30E-04	CCL3,CD83,IL1B,IL8
GATA1	1.67E-04	HBB,LMO2,PRG2,RNASE2
RELA	2.62E-04	IER3,IL1B,IL8,NFKBIA,PTPN6
PYCARD	2.91E-04	DUSP10,IL1B
TBP	5.43E-04	IER3,NFKBIA,TNFAIP3

GTF2B	8.64E-04	NFKBIA,TNFAIP3
NFKB1	1.42E-03	IER3,IL1B,NFKBIA,PTPN6
KLF2	1.93E-03	CCL3,IL1B,IL8
AHR	2.82E-03	CCNG2,CD38,DUSP6
GFI1	3.34E-03	AZU1,ELANE,IL8
GATA2	5.78E-03	PRG2,RNASE2
NFE2	7.62E-03	HBB,TXN (includes EG:116484)
SPI1 (includes EG:20375)	7.68E-03	IL1B,PRTN3,PTPN6
REL	9.69E-03	IER3,IL8
CEBPD	1.45E-02	IL1B,PRTN3
FOXN2	1.71E-02	HBB
HLTF	1.71E-02	HBB
MED7 (includes EG:171285)	1.71E-02	TNFAIP3
TRIM28	1.71E-02	S100A9
MTA2	1.71E-02	HBB
MAFK	1.71E-02	TXN (includes EG:116484)
BTG1	1.71E-02	NR3C1
BACH1	1.71E-02	HBB
MED21	1.71E-02	TNFAIP3
IRF8	1.72E-02	CD83,IL1B
SRF	3.39E-02	MCL1
NFATC4	3.39E-02	APP
CDKN2A	3.39E-02	NR3C1
KLF1	3.39E-02	HBB
KLF6	3.39E-02	COL1A1
PRDM1	3.39E-02	LGALS1
HLF	3.39E-02	LMO2
ELK1	3.39E-02	MCL1
STAT3	3.92E-02	CD83,MCL1,TIMP1
ELF4	4.12E-02	CXCL2,IL8
FOXP3	4.94E-02	ANXA1,ID2

Pair 20

Transcription Regulator	p-value of overlap	Target molecules in dataset
HMGB1	9.24E-10	CCL3,CCL4,CD83,ICAM1,IL1B,IL8,MRC1 (includes EG:100286774)
CEBPA	7.78E-07	ACSL1,ANXA1,BCL2A1,CA2,GCH1,IL1RN,MPO,PTX3,RGS2 (includes EG:19735),SOD2
KLF2	1.31E-06	CCL3,CCL4,IL1B,IL8,PTGS2
RELA	6.46E-06	IER3,IL1B,IL1RN,IL8,NFKBIA,PTGS2

TAF4B	1.04E-05	IER3,NFKBIA,TNFAIP3
IRF8	1.63E-05	BCL6,CD83,ICAM1,IL1B
STAT3	3.02E-05	CD83,FOS,IRF1 (includes EG:16362),MCL1,PTGS2,SOCS3
NFKB1	4.44E-05	IER3,IL1B,IL1RN,ITPR1,NFKBIA
SRF	1.91E-04	FOS,MCL1
ELK1	1.91E-04	FOS,MCL1
REL	2.08E-04	IER3,IL8,SOD2
TBP	2.94E-04	IER3,NFKBIA,TNFAIP3
SPI1 (includes EG:20375)	3.08E-04	CTSS,ETS2,IL1B,IL1RN
GTF2B	5.69E-04	NFKBIA,TNFAIP3
ELF4	2.16E-03	CA2,CXCL2,IL8
STAT5B	2.77E-03	BCL6,FOS
STAT1	5.40E-03	FOS,IL1B,IRF1 (includes EG:16362)
CREB1	9.69E-03	FOS,IL1B
IRF7	1.08E-02	IRF1 (includes EG:16362),MCL1,NAMPT,PELI1,RIPK2
FOXN2	1.39E-02	HBB
HSF1 (includes EG:15499)	1.39E-02	HSPA1A/HSPA1B
HLTF	1.39E-02	HBB
MED7 (includes EG:171285)	1.39E-02	TNFAIP3
MTA2	1.39E-02	HBB
FOXD1	1.39E-02	CSNK1A1
BACH1	1.39E-02	HBB
MED21	1.39E-02	TNFAIP3
BCL6	1.56E-02	BCL2A1,SOCS3
GATA1	2.27E-02	HBB,RNASE2
GFI1	2.53E-02	ETS2,IL8
NFATC4	2.75E-02	ITPR1
ZFP36	2.75E-02	FOS
PYCARD	2.75E-02	IL1B
KAT2B	2.75E-02	PTGS2
KLF1	2.75E-02	HBB
KLF6	2.75E-02	COL1A1
SATB1	3.16E-02	GADD45B,HBB,HSP90AA1,PTGS2
SP1	3.37E-02	HBB,IFNGR1,MCL1
CEBPB (includes EG:1051)	3.38E-02	IL1B,IL8
CYLD	4.10E-02	IL8

RB1	4.10E-02	HIST1H2AB/HIST1H2AE
STAT5A	4.10E-02	FOS
HSF2	4.10E-02	HSPA1A/HSPA1B
SMARCA4	4.10E-02	HBB

Pair 21

Transcription Regulator	p-value of overlap	Target molecules in dataset
CEBPA	9.49E-13	ACSL1,ANXA1,BCL2A1,BTG1,CA2,CXCR4,GBP1,GCH1,H1FX,ID2,IL1RN,ISG15,MPO,PTX3,RAB31,RGS2 (includes EG:19735), SOD2,TNFSF10
IRF7	1.29E-11	CCNA1,GBP1,IFI44,IFIH1,IFIT1,IFIT2,IFIT3,IRF1 (includes EG:16362),ISG15,NAMPT,OAS3,OASL,PELI1,PMAIP1,RIPK2,STAT1, TNFSF10
HMGB1	1.59E-08	CCL3,CCL4,CD83,ICAM1,IL1A,IL1B,IL6
STAT3	3.07E-06	CD83,FOS,IL6,IRF1 (includes EG:16362),PTGS2,PTPN2,SOCS3,VCAN
IRF3	1.18E-05	GBP1,IFI44,IFIT1,IFIT2,IFIT3,ISG15,PMAIP1
NFKB1	2.52E-05	IER3,IL1B,IL1RN,ITPR1,NFKBIA,TNFSF10
TAF4B	3.56E-05	IER3,NFKBIA,TNFAIP3
RELA	6.52E-05	IER3,IL1A,IL1B,IL1RN,NFKBIA,PTGS2
STAT1	1.77E-04	FOS,IL1B,IRF1 (includes EG:16362),IRF7,IRF8
KLF2	2.13E-04	CCL3,CCL4,IL1B,PTGS2
REL	6.91E-04	BCL2L11,IER3,SOD2
TBP	9.72E-04	IER3,NFKBIA,TNFAIP3
GTF2B	1.29E-03	NFKBIA,TNFAIP3
FOXO3	1.29E-03	BCL2L11,TNFSF10
IRF8	1.73E-03	CD83,ICAM1,IL1B
SATB1	2.14E-03	CD69,GADD45B,HSP90AA1,IRF7,IRF8,PTGS2,TSC22D3
FOXO1 (includes EG:2308)	4.17E-03	BCL2L11,KLF2
ATF2	4.17E-03	DUSP1,IL6
TP53 (includes EG:22059)	5.29E-03	IL1A,IL6,PCNA,VCAN
TAL1	5.72E-03	IL10RA,NR4A3,SERPINB2,TTC3,ZC3H12A
RORC	1.12E-02	IL1B,IL6
PPARG	1.75E-02	IL1B,TNFSF10
FOXD2	2.09E-02	PRKAR1A
HSF1 (includes EG:15499)	2.09E-02	HSPA1A/HSPA1B
MED7 (includes EG:171285)	2.09E-02	TNFAIP3
FOXD1	2.09E-02	CSNK1A1
MED21	2.09E-02	TNFAIP3

CREB1	2.11E-02	FOS,IL1B
TCF12	2.42E-02	IL10RA,NR4A3,ZC3H12A
BCL6	3.35E-02	BCL2A1,SOCS3
SRF	4.13E-02	FOS
NCOR2	4.13E-02	CD69
NFATC4	4.13E-02	ITPR1
ZFP36	4.13E-02	FOS
PYCARD	4.13E-02	IL1B
XBP1 (includes EG:140614)	4.13E-02	IL6
KAT2B	4.13E-02	PTGS2
NFKBIZ	4.13E-02	IL6
ELK1	4.13E-02	FOS

Pair 22

Transcription Regulator	p-value of overlap	Target molecules in dataset
CEBPA	1.70E-08	ACSL1,ALOX5AP,CAMP,CD14,CHI3L1,CXCR4,HCAR3,ID2,IL1RN,ITGAM (includes EG:16409),LTF,MMRN1,PTX3,S100A9,TNFSF10
GATA1	1.05E-07	AHSP,ALAS2,HBB,LMO2,MPL,PRG2,RNASE2
SPI1 (includes EG:20375)	2.43E-05	CHI3L1,CTSS,IL1RN,NCF1C,NCF2,PTPN6
CEBPE	2.70E-05	ALOX5AP,CEBPB (includes EG:1051),ELANE,LTF
IRF7	3.31E-05	CARD16,CCNA1,IFI44,IFI44L,IFIT3,IFITM1,MCL1,MX1,NAMPT,TLR4,TNFSF10
HMGB1	3.67E-05	CCL3,CCL4,CD86,HLA-DRB1,IL8
SP1	4.11E-05	ALAS2,ALOX5,CAMP,CHI3L1,HBB,IFNGR1,MCL1,NCF2
STAT3	1.14E-04	CD86,FCER1G,LILRB2,MCL1,TIMP1,TNFRSF1B,VCAN
PPARA	5.36E-04	ABCA1,NCF1C,NCF2
KLF4	6.50E-04	CD14,ITGAM (includes EG:16409)
NFKB1	7.58E-04	IER3,IFNGR2,IL1RN,PTPN6,TNFSF10
RELA	1.60E-03	IER3,IFNGR2,IL1RN,IL8,PTPN6
CEBPB (includes EG:1051)	1.78E-03	ALOX5AP,CD14,IL8,PRG2
CYLD	1.92E-03	IL8,JUN
CEBPD	2.34E-03	ALOX5AP,CD14,ITGAM (includes EG:16409)
KLF2	5.98E-03	CCL3,CCL4,IL8
ELF1	6.18E-03	FCER1G,NCF2
NFKB2	6.18E-03	CXCL12 (includes EG:20315),CXCR4
SATB1	6.40E-03	CEACAM1 (includes others),EPSTI1,EVI2A,HBB,HSP90AA1,HSPA8,IRF8
JUN	7.22E-03	JUN,NCF2,VEGFA
AHR	8.61E-03	DUSP6,ITGAM (includes EG:16409),JUN
GFI1	1.01E-02	ELANE,IL6R,IL8
GATA2	1.25E-02	PRG2,RNASE2
IRF3	1.56E-02	ADAM9,AHNAK,IFI44,IFIT3
NOTCH1	1.64E-02	GYPA,ITGAM (includes EG:16409)
REL	2.08E-02	IER3,IL8
FOXN2	2.55E-02	HBB
NR1H3	2.55E-02	ABCA1
TFAP2A	2.55E-02	ABCA1
HLTF	2.55E-02	HBB
PLAGL2	2.55E-02	NCF2
TRIM28	2.55E-02	S100A9
MTA2	2.55E-02	HBB
RBPJ	2.55E-02	CAMP

BACH1	2.55E-02	HBB
NR1D1	2.55E-02	TLR4
PPARG	2.56E-02	ABCA1,TNFSF10
STAT1	2.78E-02	ALAS2,CD14,IRF8
IRF8	3.62E-02	CCR7,CD86
SP3	4.83E-02	CHI3L1,NCF2

Pair 23

Transcription Regulator	p-value of overlap	Target molecules in dataset
CEBPA	9.92E-07	AKAP12,CA2,CD14,CDKN1A,CEBPA,FOXO1 (includes EG:2308),GATA2,GLIPR1,H1FX,HCAR3,IFI27,ISG15,KLRC1,LGALS1, MPO,NFIL3,PRTN3,PTGS2,RGS2 (includes EG:19735),S100A9,STAR,TRD@,TUBB2A
GATA1	4.25E-06	AHSP,BCL2L11,EPOR,FCER1A,GYPA,GYPB,NFE2,PF4,PRG2,RNASE2
HNF4A	1.43E-05	AK2,AKR1C1/AKR1C2,AKR1C3,APOC2,CAMK2D,CDC42EP3,CDKN1A,CEBPA,CTSA,CXCL3,EGR1,ERLIN1,FAM107B,FAM46A, FOXO1 (includes EG:2308),G0S2,GAB1,GLA,GPR160,GSPT1,GSTK1,GUSB,HIF1A,HIST1H4A (includes others),HMGB2, HNRNPA0,HSP90B1,IL2RB,ITGB3BP,JUN,KLRF1,LDLR,MDH2 (includes EG:17448),MFSD1,MGST1,MRPL33 (includes EG:66845),NAMPT,NDFIP1,PLSCR1,RBKS,REXO2,RNASE2,RNASE3,RPS6KA5,RRM1,S100A9,SLC38A1,STAT1, TAX1BP1,TCF7L2,TMEM30A
EBF1	2.71E-05	CD79A,CD79B,IGLL1/IGLL5,PAX5
GFI1	2.82E-05	AZU1,CDKN1A,CEBPA,GFI1B,IL6R,IL8,SERPINA1
LYL1	5.60E-05	CCND3,EGR1,ID1,ID3 (includes EG:15903),RAPGEF2
ATF3	5.70E-05	ATF3,CCL5,CDKN1A,ID1,JUN,LDLR,PENK
ELK1	5.70E-05	CDKN1A,EGR1,FUT4,JUN,MCL1,PTGS2,TNFRSF10B
EPAS1	1.04E-04	ADM,AKAP12,CXCL2,ENO1,FHL1 (includes EG:14199),HIST1H1C,HIST1H2AC,HOXA5,MAFF,NFIL3,PLIN2,SLC2A1,VEGFA
STAT5a/b	1.14E-04	BCL2L11,CDKN1A,FCGR1A,GFI1B,GYPA,GYPB,IRF1 (includes EG:16362),PTGS2
SPI1 (includes EG:20375)	1.74E-04	FCGR2B,FES,HMBS,IGL@,IL1B,IRF4,ISG15,MME,PRG2,PRTN3
IRF9	2.53E-04	IFI27,IL8,IRF7,ISG15,STAT1
ATF4	3.07E-04	ATF3,DDIT4,JUN,PENK,PTGS2,TNFRSF10B,VEGFA
DACH1	3.78E-04	CDKN1A,CXCL3,EGR1,JUN,MEF2C
RARB	4.08E-04	CDKN1A,CXCL2,ID1,ID3 (includes EG:15903),IL1B,IL8,KRT1,PTGS2
KLF2	4.10E-04	ADM,CCND3,HIF1A,ID1,ID3 (includes EG:15903),IL1B,IL8,LTC4S,MYH10,PTGS2,RALA,RNASE2,RUNX2,SLC2A1,STAT1
ISGF3	4.48E-04	HIF1A,IRF7,ISG15
PYCARD	4.48E-04	DUSP10,IL1B,IL8
HIF3A	4.48E-04	HIF1A,SLC2A1,VEGFA

NFkB (complex)	6.08E-04	ATF3,BCL2L11,CCL5,CDKN1A,CXCL12 (includes EG:20315),CXCL2,CXCL3,EPCAM,ERAP2,FAM46A,FCGR1A,G0S2,HDGF, HIF1A,IER3,IGFBP2,IL1B,IL8,IRF1 (includes EG:16362),IRF4,IRF7,JUN,LTC4S,MYLK,PTGS2,TNFRSF10B,TSLP,VEGFA
NFKB1	6.09E-04	APP,CCL5,CDKN1A,CXCL2,IER3,IL1B,IL8,IRF1 (includes EG:16362),IRF4,LTC4S,PTGS2,TSLP,VEGFA
E2F1	6.14E-04	BCL2L11,CA2,CCND3,CD9,CDKN1A,DUSP10,DUT,E2F2,EIF2AK2,FHL1 (includes EG:14199),HIST1H2AC,HSP90B1,ID3 (includes EG:15903),KIAA0101,KRT1,MAD2L1,MCL1,RRM1,RRM2,RYBP,TCF3,TOP2A
CREBBP	7.12E-04	CCL5,CDKN1A,EGR1,IRF1 (includes EG:16362),ISG15,JUN,LDLR,PTGS2,STAR
MEOX2	7.36E-04	CDKN1A,CDKN1C,CXCL2,CXCL3,ID1,ID3 (includes EG:15903),IL8
SMAD5	9.35E-04	GATA2,ID1,KLF1,NFE2
STAT1	1.00E-03	BCL2L11,CD14,CDKN1A,FCGR1A,IFI27,IL1B,IL8,IRF1 (includes EG:16362),IRF7,ISG15,S100A10,TNFRSF10B
MYC	1.06E-03	ADM,BCAT1,CCND3,CD44 (includes EG:100330801),CDKN1A,CEBPA,CYFIP2,DNTT,E2F2,ENO1,FABP5,HIF1A,HIST1H4A (includes others),HSPB1,ID1,IER3,IGLL1/IGLL5,IL8,IRF7,LGALS1,TFRC,TNFRSF10B,VEGFA
Notch	1.08E-03	ADM,CDKN1A,ID1,MCL1,NET1 (includes EG:10276),SMAD1,VEGFA
STAT3	1.38E-03	BCL2L11,CCL5,CDKN1A,FCGR1A,HGF,HIF1A,IL2RB,IL8,IRF1 (includes EG:16362),IRF4,LILRB2,MCL1,NAMPT,PTGS2,STAT1,VEGFA
IFI16	1.43E-03	CCL5,CDKN1A,E2F2,IL1B,IL2RB,IL8,LDLR,MGST1,MPO
EGR1	1.43E-03	BCL2L11,CD44 (includes EG:100330801),CDKN1A,EGR1,IL8,JUN,LDLR,PTGS2,VEGFA
RELA	1.81E-03	CCL5,CDKN1A,CXCL2,CXCL3,EGR1,ERAP2,HIF1A,IER3,IL1B,IL8,IRF1 (includes EG:16362),IRF4,LTC4S,NAMPT,PTGS2, TNFRSF10B,TSLP,VEGFA
ELF4	1.98E-03	CA2,CDKN1A,CXCL2,HDC,IL8,PRF1
ATF6	2.04E-03	APP,HSP90B1,SERPINA1,VEGFA
TAF4B	2.08E-03	IER3,IGJ,IRF1 (includes EG:16362)
CALR	2.08E-03	CDKN1A,CEBPA,PTGS2
STAT2	2.30E-03	IFI27,IL8,IRF1 (includes EG:16362),ISG15,MX1
FOXG1	2.39E-03	BCL2L11,CDKN1A
HDAC9	2.39E-03	CEBPA,JUN
HOXA9	2.56E-03	BCL2L11,FLT3,GATA2,GLRX,HNRNPU,HOXA9,IRX3,JUN,MAFB,MBNL1,NFIA,S100A8,TCN1,USP1,YWHAG
TP53 (includes EG:22059)	2.84E-03	ADA,ATF3,BCL2L11,CCND3,CD44 (includes EG:100330801),CDKN1A,CEBPA,DDIT4,DUSP2,DUT,EGR1, FOXO1 (includes EG:2308),GLIPR1,HDC,HIF1A,ID1,IER3,IGFBP2,IGFBP7,IL8,IRF7,ISG15,MAD2L1,MCL1,MX1, PTGS2,PYCARD,SLC2A1,TCF7L2,TNFRSF10B,TOP2A,VEGFA
FLI1	2.89E-03	APOC2,CCND3,EGR1,PF4,SRGN
IRF1 (includes	3.35E-03	CCL5,CDKN1A,IFITM3,IL1B,IL8,IRF1 (includes EG:16362),IRF4,IRF7,ISG15,MX1

EG:16362)		
REL	3.37E-03	BCL2L11,CDKN1A,IER3,IL8,IRF4,JUN,TNFRSF10B
Stat1-Stat2	3.51E-03	EIF2AK2,IRF1 (includes EG:16362),IRF7
CTCF (includes EG:10664)	3.51E-03	APP,CDKN1C,HLA-DRB1
NfkB-RelA	3.60E-03	CCL5,CXCL3,IL1B,IL8,PTGS2
ARNT	3.60E-03	AHR,ENO1,HIF1A,TFRC,VEGFA
FOXO4	3.60E-03	CDC42EP3,CDKN1A,HIF1A,IER3,VEGFA
HIF1A	3.63E-03	ADM,CDKN1A,ENO1,FHL1 (includes EG:14199),HIF1A,HIST1H1C,HIST1H2AC,IL8,LGALS1,MAFF,MCL1,SLC2A1, SLC40A1,TFRC,VEGFA
ZBTB17	3.81E-03	CDKN1A,EGR1,IER3,SPTBN1
HDAC1	4.92E-03	CCL5,CCNA1,CD44 (includes EG:100330801),CDKN1A,IL8,PTGS2,RRM2,TAL1
RELB	5.00E-03	CXCL12 (includes EG:20315),IL1B,IL8,IRF4
CEBPB (includes EG:1051)	5.01E-03	CD14,CDC42EP3,CEBPA,ID1,IL1B,IL8,LDLR,PRG2,PRTN3,PTGS2,SERPINA1,STAR
Cyclin E	5.41E-03	CDKN1A,EGLN1,HIF1A
ZFP36	5.41E-03	CDKN1A,HIF1A,PTGS2
ELF1	5.41E-03	DNTT,FCER1A,PF4
NFE2	5.41E-03	HMBS,KLF1,SPTA1
MLL	5.41E-03	HOXA7,HOXA9,MEIS1
IRF7	5.41E-03	CCL5,CCNA1,IFI44,IFI44L,IFITM3,IRF1 (includes EG:16362),ISG15,MCL1,MX1,NAMPT,S100A8,STAT1
Betacatenin/TCF	6.93E-03	JUN,LEF1
TSC22D1	6.93E-03	IL1B,IL8
TAF12 (includes EG:386637)	6.93E-03	IGJ,IRF1 (includes EG:16362)
Ap1	7.57E-03	ATF3,CXCL12 (includes EG:20315),GZMB,HSP90B1,IL1B,IL8,JUN,PTGS2,TSLP,VEGFA
Cbp/p300	7.82E-03	CDKN1A,IRF1 (includes EG:16362),PTGS2
IKZF1	7.82E-03	CCND3,DNTT,FLT3
HDAC4	8.07E-03	MEF2C,PTGS2,SLC2A1,VEGFA
SRF	8.91E-03	AKAP12,CDKN1A,EGR1,GLIPR1,JUN,MCL1,MEIS1,MGST1,MYLK,PTGS2
SREBF2	9.98E-03	CDKN1A,LDLR,PLIN2,STAR
PAX5	1.08E-02	BLNK,CD79A,IGLL1/IGLL5
KLF1	1.08E-02	BCL11A,CD44 (includes EG:100330801),CDKN1A
ID2	1.08E-02	BCL2L11,CD79A,CDKN1A
SP1	1.19E-02	CDKN1A,CXCL12 (includes EG:20315),EIF2AK2,FES,HDC,HGF,HIST1H4A (includes others),IFITM3,IL1B,IL2RB,IL8, IRF1 (includes EG:16362),IRF4,LDLR,MCL1,PTGS2,SPARC,STAR,TNFRSF10B,VEGFA
ESR1	1.24E-02	CDKN1A,CXCL3,EGR1,ENO1,IER3,IFI27,IL8,IRF4,LDLR,PTGS2,SOCS2,VEGFA

PPARG	1.30E-02	APP,CDKN1A,CEBPA,IL1B,IL8,LDLR,MPO,PLIN2,PTGS2,VEGFA
TAF9	1.34E-02	IL8,IRF1 (includes EG:16362)
COPS5	1.34E-02	CDKN1A,HIF1A
TBX1	1.34E-02	CDKN1A,LEF1
CEBPD	1.41E-02	CD14,CDKN1C,IL1B,PRTN3,PTGS2
ATF2	1.41E-02	ATF3,DUSP10,JUN,PENK,PTGS2
PBX1	1.43E-02	CDKN1A,HOXA3,TCF7L2
Creb	1.46E-02	EGR1,JUN,KRT1,LDLR,PENK,POU2AF1,PTGS2,VEGFA
PPARD	1.46E-02	IGFBP2,PTGS2,VEGFA,YWHAE
TFDP1	1.46E-02	CCNA1,CCND3,CDKN1A,TCF3
FOXO1 (includes EG:2308)	1.62E-02	BCL2L11,CDC42EP3,CDKN1A,CDKN1C,DDIT4,FOXO1 (includes EG:2308),IER3,IL17RA,JUN,NUSAP1,RUNX2
TWIST1	1.74E-02	CDKN1A,RUNX2,TCF3,ZEB2
SUPT16H	1.74E-02	CDKN1A,EGR1,HIST1H1C,HIST1H2AC
MYOD1	1.74E-02	BCL2L11,CDKN1C,MAFB,RBM38
HLX	1.84E-02	CDKN1C,EGR1,JUN
TP63	2.12E-02	AHR,BCL2L11,CDKN1A,CSTA,ID1,IL8,ITGA4,MAFF,PPBP,S100A8,TNFRSF10B,VEGFA
Foxo	2.16E-02	BCL2L11,CDKN1A
Stat1 dimer	2.16E-02	FCGR1A,IRF1 (includes EG:16362)
TEAD4	2.16E-02	JUN,VEGFA
MEIS1	2.16E-02	CDKN1A,PF4
PAWR	2.16E-02	APP,TNFRSF10B
ENO1	2.16E-02	ENO1,PTGS2
SP3	2.25E-02	CDKN1A,CEBPA,EIF2AK2,HGF,IL2RB,LDLR,PTGS2,SPARC,VEGFA
Smad	2.30E-02	CDKN1A,EGLN1,VEGFA
MECP2	2.30E-02	CCNA1,PLA2G16,VEGFA
NFKB2	2.30E-02	CDKN1A,CXCL12 (includes EG:20315),IL8
RORC	2.30E-02	HIF1A,IL1B,VEGFA
NR4A1	2.35E-02	ATF3,BRE,CXCL12 (includes EG:20315),HIF1A,LDLR,STAR
EZH2	2.37E-02	BCL2L11,CCND3,CDK6,CDKN1A,CXCL2,IL8,MNDA,PRG2,PTGS2,SERPINA1,SPIB,WASF1
RUNX1	2.64E-02	BCL2L11,CDKN1A,CEBPA,NFE2,VEGFA
FOS	2.74E-02	CSTA,EGR1,IL8,JUN,PENK,PTGS2,PTPRO,VEGFA
TFEB	2.75E-02	CTSA,GLA,NEU1,TPP1 (includes EG:1200)
NCOR2	2.83E-02	CDKN1A,PTGS2,STAR
PRDM1	2.83E-02	ID3 (includes EG:15903),LGALS1,SPIB
IRF3 dimer	3.14E-02	CCL5,ISG15
CYLD	3.14E-02	IL8,JUN
POU2AF1	3.14E-02	CD79A,CD79B
STAT6 (includes EG:20852)	3.28E-02	CCL23,CCL5,FCGR1A,IRF1 (includes EG:16362),IRF4

MYBL2	3.41E-02	CCNA1,CDC25B,TOP2A
THRA	3.41E-02	APP,CDKN1A,JUN
HOXA10	3.85E-02	BCL2L11,CDKN1A,CSTA,HLA-DQA1,HMBS,IRX3,P4HB,S100A12
E2F4	3.86E-02	CCNA1,CDKN1A,DUT,E2F2,HIST1H2AC,HSP90B1,ID3 (includes EG:15903),KIAA0101,MAD2L1,MCL1,PLSCR1,RRM1,RRM2, TMPO,TOP2A
N-cor	4.25E-02	CDKN1A,PTGS2
ZNF148	4.25E-02	CDKN1A,STAT1
PITX2	4.25E-02	FOXO1 (includes EG:2308),LEF1
FHL2	4.25E-02	BCL2L11,IL8
DAXX	4.25E-02	CDKN1A,HSPB1
RARA	4.40E-02	CCNA1,CDKN1A,PENK,PTGS2,TNFRSF10B
KDM5B	4.46E-02	ARL6IP5,FABP5,FHL1 (includes EG:14199),IGFBP2,ISG15,LGALS3BP,PLS3,TOP2A,TUBB2A
STAT	4.56E-02	CDKN1A,EIF2AK2,IRF1 (includes EG:16362),SOCS2
ETS1	4.64E-02	APOC2,CD14,CD79A,CDKN1A,EGR1,HGF,SRGN,VEGFA
AHR	4.70E-02	CDKN1A,IL1B,IRF1 (includes EG:16362),JUN,PTGS2,VEGFA
E2F5	4.74E-02	CCNA1,CDKN1A,DUSP10
BCL6	4.81E-02	BACH2,CDKN1A,IL2RB,IRF4,PTPRO
HMGB1	4.81E-02	CDKN1A,CXCL3,HLA-DRB1,IL1B,IL8
ASB2	4.89E-02	HOXA9
ZNF384	4.89E-02	RUNX2
SPIC	4.89E-02	FCGR2B
NKRF	4.89E-02	IL8
KDM4A	4.89E-02	FHL1 (includes EG:14199)
FANK1	4.89E-02	JUN
KLF16	4.89E-02	CDKN1A
SMAD1/5	4.89E-02	ID1
JDP2	4.89E-02	JUN
Atf	4.89E-02	JUN
ELL	4.89E-02	CDKN1A
CITED4	4.89E-02	HIF1A
SALL2	4.89E-02	CDKN1A
HBP1	4.89E-02	CDKN1A
MEF2BNB-MEF2B	4.89E-02	JUN
AATF	4.89E-02	APP
ING4	4.89E-02	CDKN1A
ZNF91	4.89E-02	FCGR2B
SRCAP	4.89E-02	CDKN1A
ZNF197	4.89E-02	HIF1A
TEF	4.89E-02	MYLK

ZHX2	4.89E-02	HDC
NKX3-2	4.89E-02	RUNX2
SNW1	4.89E-02	CDKN1A
ATF5	4.89E-02	ID1
ZNF140	4.89E-02	FCGR2B
IRF3	4.92E-02	AHNAK,CCL5,IFI44,IL8,IRF7,ISG15,TMPO,TSLP
